# Genome-Wide Assessment of a Korean Composite Pig Breed, Woori-Heukdon

**DOI:** 10.3389/fgene.2022.779152

**Published:** 2022-02-02

**Authors:** Yong-Min Kim, Ha-Seung Seong, Young-Sin Kim, Joon-Ki Hong, Soo-Jin Sa, Jungjae Lee, Jun-Hee Lee, Kyu-Ho Cho, Won-Hyong Chung, Jung-Woo Choi, Eun-Seok Cho

**Affiliations:** ^1^ Swine Science Division, National Institute of Animal Science, Rural Development Administration, Cheonan, South Korea; ^2^ Department of Animal Science, College of Animal Life Sciences, Kangwon National University, Chuncheon, South Korea; ^3^ Department of Animal Science and Technology, College of Biotechnology and Natural Resources, Chung-Ang University, Anseong, South Korea; ^4^ Institute of Agriculture and Life Science, College of Agriculture and Life Sciences, Gyeongsang National University, Jinju, South Korea; ^5^ Research Group of Healthcare, Korea Food Research Institute, Wanju, South Korea

**Keywords:** Woori-Heukdon, Korean native pig, genetic diversity, runs of homozygosity, selection signature, synthetic breed

## Abstract

A Korean synthetic pig breed, Woori-Heukdon (WRH; F3), was developed by crossing parental breeds (Korean native pig [KNP] and Korean Duroc [DUC]) with their crossbred populations (F1 and F2). This study in genome-wide assessed a total of 2,074 pigs which include the crossbred and the parental populations using the Illumina PorcineSNP60 BeadChip. After quality control of the initial datasets, we performed population structure, genetic diversity, and runs of homozygosity (ROH) analyses. Population structure analyses showed that crossbred populations were genetically influenced by the parental breeds according to their generation stage in the crossbreeding scheme. Moreover, principal component analysis showed the dispersed cluster of WRH, which might reflect introducing a new breeding group into the previous one. Expected heterozygosity values, which were used to assess genetic diversity, were .365, .349, .336, .330, and .211 for WRH, F2, F1, DUC, and KNP, respectively. The inbreeding coefficient based on ROH was the highest in KNP (.409), followed by WRH (.186), DUC (.178), F2 (.107), and F1 (.035). Moreover, the frequency of short ROH decreased according to the crossing stage (from F1 to WRH). Alternatively, the frequency of medium and long ROH increased, which indicated recent inbreeding in F2 and WRH. Furthermore, gene annotation of the ROH islands in WRH that might be inherited from their parental breeds revealed several interesting candidate genes that may be associated with adaptation, meat quality, production, and reproduction traits in pigs.

## Introduction

In the swine industry, crossbreeding has been widely used to exploit the phenomenon of heterosis, or hybrid vigor. The main benefit of the heterosis is increased performance of the resulting crossbred offspring over the average performance of its purebred parent pigs in traits of interest (Johnson, 1981; Falconer and Mackay, 1996). Although not all pig traits that were targeted for hybrid vigor show the same degree of heterosis, there has been significant success in harnessing heterosis to improve productivity of several economically important traits (Baas et al., 1992; Cassady et al., 2002a; Cassady et al., 2002b). Since 1970, most commercial pig producers have been using a classical terminal crossbreeding system with three breeds (Landrace × Yorkshire dam × Duroc sire, or LYD) to produce market pork in South Korea. The LYD system resulted in higher productivities, including increased growth rate, by taking advantage of crossbreeding (Jin et al., 2006; Choi et al., 2016).

**FIGURE 1:**
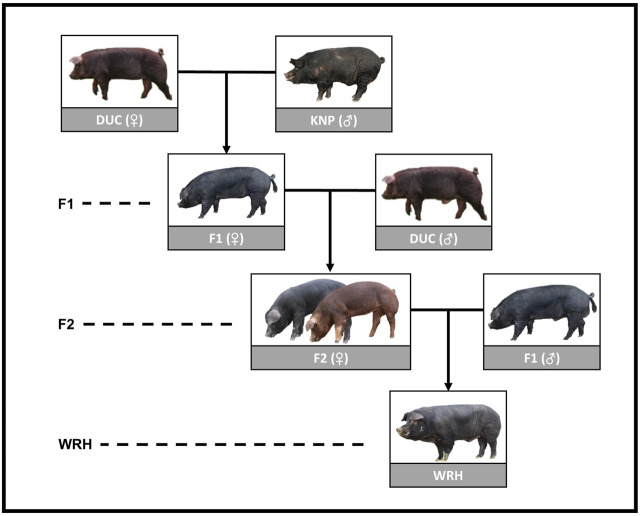
Crossbreeding scheme of WRH using parental breeds (DUC and KNP) and their crossbred populations (F1 and F2). DUC, Korean Duroc; KNP, Korean native pig; F1, DUC × KNP; F2, F1 × DUC; WRH, Woori-Heukdon (F1 × F2).

In recent years, the South Korea swine industry has been partly changing from emphasis on efficiently producing more pork, mostly by focusing on growth and reproduction, to addressing taste-oriented consumption. There has been consistent consumer demand in Korea for diversified and great-tasting pork, despite its higher price than the typical market pork that is mostly derived from the LYD system (Kim S. G. et al., 2020). In response to such demand, the National Institute of Animal Science in Korea developed Woori-Heukdon (WRH), which is a synthetic breed derived from crossbreeding Duroc (DUC) and the Korean native pig breed (KNP) (Kim Y.-M. et al., 2020). KNP is known to have great meat qualities, such as high glucose content and a high unsaturated/saturated fatty acid ratio. However, because it also has economically unfavorable characteristics, such as slow growth rate, late maturity, and light carcass weight, it has low productivity compared with the commercial breeds; therefore, the population has decreased (Park et al., 2007; Hur et al., 2013). To take advantage of genetic merits of both the Duroc and KNP populations, WRH were generated by the crossing scheme shown in Figure 1. F2 was shown to have a slightly better growth rate compared with WRH; however, meat qualities, such as meat color and shear force, were clearly better in WRH than F2. Therefore, WRH preserved the characteristics of KNP, including superior meat quality, and had an improved growth rate compared with KNP (Kim et al., 2016).

Runs of homozygosity (ROH) are contiguous homozygous stretches that are inherited from each parent. Although ROHs are known to arise from several genetic factors, including genetic drift and population bottlenecks, it has been used as an indicator of selection signatures throughout the genome. In fact, ROHs have been widely used to quantify autozygosity in pigs (Schachler et al., 2020; Wu et al., 2020); because ROH are widely but not randomly distributed across the genome, some ROH overlap with genomic regions associated with economically important traits in pigs. Furthermore, ROH can be used to distinguish between recent and ancient inbreeding (Keller et al., 2011). In particular, shared ROH within a population can be used to identify genomic regions potentially under selection, which could be associated with the environment or production systems. Several studies have assessed autozygosity in livestock species using ROH approaches (Peripolli et al., 2018; Xu Z. et al., 2019; Dzomba et al., 2021). Offspring inherit chromosomal segments from the same ancestor by descent (Broman and Weber, 1999). Consequently, the extent of ROH can be used to estimate the inbreeding coefficient (Bosse et al., 2012; Marras et al., 2015).

Currently, there is still a lack of studies to elucidate genomic characteristics of the composite breeds that were generated by using the indigenous pigs. The main objective of this study were: 1) to estimate various parameters to clarify genomic population structure of the crossbreds with DUC and KNP populations; and 2) to detect and investigate ROH that could be an indicative of selection signature in WRH population.

## Materials and Methods

### Animals and Genotyping

A total of 2,074 pigs (male and female) were used in this study, including DUC, KNP, and their crossbred F1 (DUC × KNP), F2 (F1 × DUC), and F3 (WRH; F1 × F2) populations. The dataset includes previously published genotype data (Kim Y.-M. et al., 2020) that contained 61,565 SNPs for DUC (N = 50), KNP (N = 50), and the composite breed WRH (F3; N = 100). We also generated whole blood samples from 1,874 individuals of DUC (N = 1,029), KNP (N = 158), F1 (N = 11), F2 (N = 144), and WRH (F3; N = 532) at the National Institute of Animal Science, Rural Development Administration, Korea. The genomic DNA was extracted from the blood samples using the phenol–chloroform method and genotyped using the Illumina PorcineSNP60 BeadChip v2 (Illumina, Inc., San Diego, CA, United States), which contains 61,565 SNPs. Genotype data were called using the genotype module in GenomeStudio v2.0 (Illumina, Inc.). The SNP coordinates were updated from the genome assembly of *Sus scrofa* (Sscrofa10.2 to Sscrofa11.1) according to manifest file of Illumina PorcineSNP60 v2 using PLINK v1.9 (Chang et al., 2015).

### Quality Control of Genotype Data

The quality control (QC) process for the genotype dataset was conducted separately for SNPs and animals using PLINK v1.9. We applied three different QC procedures for the initial raw SNP dataset to further analyze genetic diversity, population structure, and ROH. All three genotype subsets used the following process: 1) SNPs in sex chromosomes or unmapped in Sscrofa11.1; 2) SNPs with a call rate less than 90%. Individuals with a call rate less than 90% were removed. The ROH analysis was conducted using a subset of data without additional QC process. To assess genetic diversity, we also removed SNPs with a minor allele frequency (MAF) less than .05. Furthermore, SNP filtering based on pairwise linkage disequilibrium (LD) was conducted to minimize the reduction of informativeness of the dataset (Lopes et al., 2013) using the indep-pairwise 50 5 0.5 command for population structure analyses.

### Genetic Diversity

To assess genetic diversity within populations, we calculated observed heterozygosity (H_O_), expected heterozygosity (H_E_), and individual inbreeding coefficient (F_HOM_) using PLINK v1.9. The inbreeding coefficient was calculated based on homozygous genotypes as follows:

$$F_{HOM}=\frac{Observed\;\mathrm{hom}ozygous\;loci-\;Expected\;\mathrm{hom}ozygous\;loci}{Total\;number\;of\;nonmissing\;loci-\;Expected\;\mathrm{hom}ozygous\;loci}$$

### Population Structure

To explore the pattern of genetic differentiation of samples, we conducted principal component analysis (PCA) using PLINK v1.9, and the first two principal components (PCs) were visualized using R v4.1.0 (www.r-project.org, accessed July 4, 2021). The PCA plot was also considered a QC process because it reveals potential misclassification.

To better understand the relationship among parental breeds and their crossbred populations, an admixture analysis for K = 2 that is based on the number of ancestral populations was performed using ADMIXTURE v1.3 (Alexander and Lange, 2011).

### ROH Detection

To detect ROH, we used a dataset of 56,498 SNPs for 2,040 individuals that resulted from QC without filtering based on MAF and LD because of problematic factors in ROH discovery (Marras et al., 2015; Meyermans et al., 2020). To avoid short and common ROH caused by LD, we set the minimum length of ROH to 1 Mb for ROH discovery, as described by several studies (Purfield et al., 2012; Ferencakovic et al., 2013; Marras et al., 2015; Meyermans et al., 2020). ROHs were identified using a sliding window method with the homozyg command in PLINK v1.9. The parameters used in ROH detection were applied as follows: 1) the minimum length of ROH was 1 Mb (--homozyg-kb); 2) an ROH had at least one SNP per 50 kb on average (--homozyg-density); 3) the distance of consecutive SNPs in the same ROH was less than 1,000 kb (--homozyg-gap); 4) no heterozygous SNP (--homozyg-window-het and--homozyg-het) and one SNP with a missing genotype were allowed (--homozyg-window-missing). In addition, the thresholds for the minimal SNP numbers per window (--homozyg-window-snp) and in the ROH (--homozyg-snp) were calculated by the L-parameter for the populations (Lencz et al., 2007; Purfield et al., 2012; Meyermans et al., 2020). In this study, we classified ROH into three categories based on their physical length: short (1 to <3 Mb), medium (3 to <5 Mb) and long (>10 Mb).

ROH islands, which were defined as regions where SNPs in ROHs had *p*-values higher than a specific threshold for each population, were marked as a potential selection signature, and ROH islands were determined using R-script at the Open Science Framework (https://doi.org/10.17605/OSF.IO/XJTKV) provided by Gorssen et al. (2021). The population-specific threshold was determined based on z-scores obtained from the distribution of ROH incidences. Additionally, the top 0.1% of SNPs (*p*-value >.999) were used to form ROH islands (Purfield et al., 2017; Gorssen et al., 2020). Furthermore, a threshold that a ROH should be included in at least 30% of individuals within each population was set for ROH islands. For highly inbred populations such as KNP (F_ROH_ = 41%), the threshold was set to 80% because SNPs with *p*-values higher than .999 were not found in this population, as described by Gorssen et al. (2021).

Using discovered ROHs, we calculated the inbreeding coefficient based on ROH using the following calculation proposed by McQuillan et al. (2008):

$$F_{ROH}=\frac{L.ROH}{L.Autosomes}$$

Where L. ROH is the sum of all ROH of an individual and L. Autosomes is the total length of autosomal genome covered by SNPs (in this study, 2,262.6 Mb). Furthermore, we investigated patterns of inbreeding estimates based on homozygosity and ROH by calculating Pearson’s correlations.

### Annotation of Genes and Quantitative Trait Loci

Gene annotation for ROH islands was conducted according to NCBI Sus scrofa Release 106 (https://ftp.ncbi.nlm.nih.gov/genomes/all/GCF/000/003/025/GCF_000003025.6_Sscrofa11.1/GCF_000003025.6_Sscrofa11.1_genomic.gff.gz, accessed on 15 June 2021). Furthermore, we also used pig quantitative trait locus information for ROH islands to annotate potential traits associated with selected regions (https://www.animalgenome.org/, accessed on 20 July 2021).

## Results

### SNP Characteristics

The SNP coordinates for each chromosome (SSC) were updated from Sscrofa10.2 to Sscrofa11.1 according to manifest file provided from Illumina (Supplementary Table 1). After initial QC steps, three additional individuals (DUC = 3) were removed because of breed misclassification according to PCA (Supplementary Figure 1). As a result of the QC procedure, we retrieved three SNP subsets for population structure (12,801 SNPs for 2,040 individuals), genetic diversity (43,809 SNPs for 2,040 individuals), and ROH analyses (56,498 SNPs for 2,040 individuals).

### Population Structure

**FIGURE 2:**
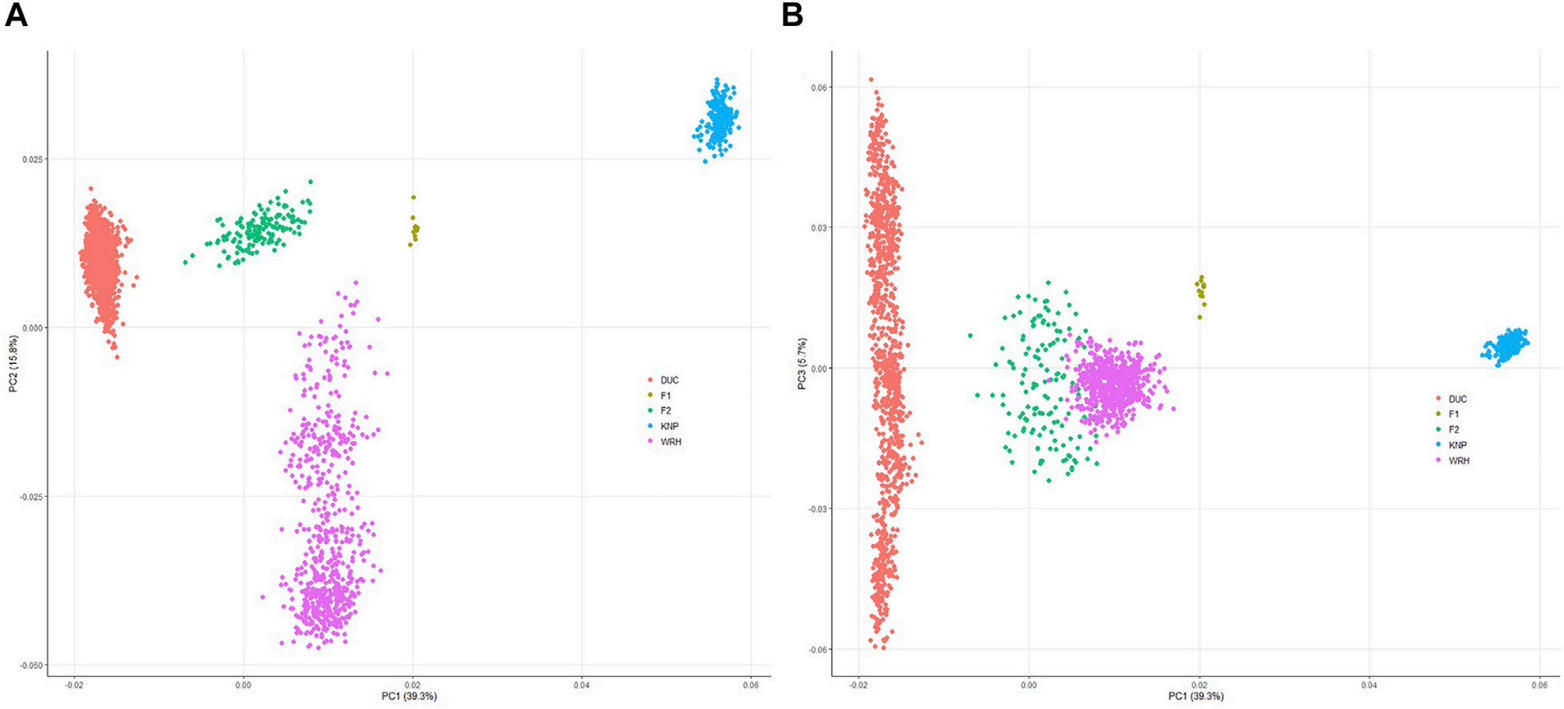
Principal component analysis illustrating stratification of the five pig populations. **(A)** Visualization of PC1 vs PC2, which explains 55.1% of total variation together; **(B)** visualization of PC1 vs PC3, which explains 45%. DUC, Korean Duroc; KNP, Korean native pig; F1, DUC × KNP; F2, F1 × DUC; WRH, Woori-Heukdon (F1 × F2).

**FIGURE 3:**
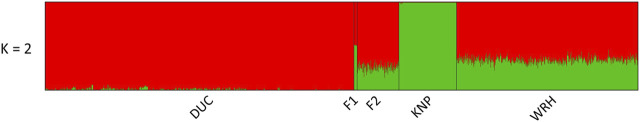
Population structure analysis based on ADMIXTURE at K = 2. Each column represents an individual. DUC, Korean Duroc; KNP, Korean native pig; F1, DUC × KNP; F2, F1 × DUC; WRH, Woori-Heukdon (F1 × F2).

The top three PCs (PC1, PC2, and PC3) explained approximately 39.3, 15.8, and 5.7% of total variation, respectively. As shown in Figure 2A, DUC and KNP were clearly separated, and the cluster of F1 was located in the middle of DUC and KNP populations based on PC1. F2 and WRH were also located between DUC and KNP; however, both populations were closer to DUC. Furthermore, WRH showed a dispersed cluster based on PC2. Clustering patterns for (F1, F2, and WRH) were clear based on PC1 and PC3, which explained approximately 39.3 and 5.7% of the total variation, respectively (Figure 2B). A similar clustering pattern was observed in ADMIXTURE analysis, showing distinct ancestries of 99.3 and 100.0% in DUC and KNP, respectively (Figure 3; Supplementary Table 2). For their crossbreds, F1, F2, and WRH had 51.1, 25.9, and 34.1% KNP ancestry, respectively, at K = 2.

### Genetic Diversity and Inbreeding

Genetic diversity estimates using a subset of 43,809 SNPs for 2,040 individuals are summarized in Table 1. All crossbred populations (F1, F2, and WRH) had higher heterozygosity rates than the initial parental breeds (DUC and KNP). F1 had the highest average H_O_ value (.477 ± .297), followed by WRH (.407 ± .164), F2 (.380 ± .163), DUC (.331 ± .160), and KNP (.223 ± .216). The average H_E_ value was highest in WRH (.365 ± .133) and lowest in KNP (.211 ± .202). The inbreeding coefficient values (F_HOM_) were shown to have negative values in all five populations, exhibiting the highest value in DUC (−.002 ± .053), followed by WRH (−.024 ± .083), KNP (−.055 ± .104), F2 (−.115 ± .048), and F1 (−.417 ± .019).

**TABLE 1:** Estimates of genetic diversity and inbreeding for five pig populations.

Pop	No. before QC^a^	H_O_ ± SD	H_E_ ± SD	F_HOM_ ± SD
DUC	1,079	0.331 ± 0.160	0.330 ± 0.159	−0.002 ± 0.053
KNP	208	0.223 ± 0.216	0.211 ± 0.202	−0.055 ± 0.104
F1	11	0.477 ± 0.297	0.336 ± 0.163	−0.417 ± 0.019
F2	144	0.380 ± 0.163	0.349 ± 0.138	−0.115 ± 0.048
WRH	632	0.407 ± 0.164	0.365 ± 0.133	−0.024 ± 0.083

a Number of samples before quality control (missing genotype rate <90%). Pop, population; H_O_, observed heterozygosity; H_E_, expected heterozygosity; F_HOM_, inbreeding coefficient based on excess of homozygosity; SD, standard deviation; DUC, Korean Duroc; KNP, Korean native pig; F1, DUC × KNP; F2, F1 × DUC; WRH, Woori-Heukdon (F1 × F2).

### ROH Patterns

**FIGURE 4:**
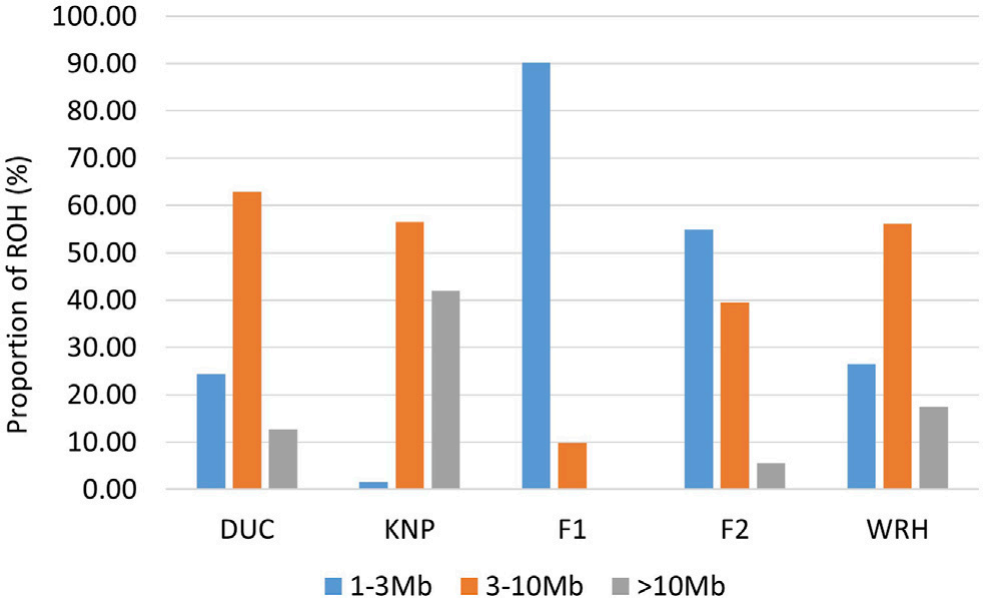
ROH distribution according to size classification (1–3 Mb, 3–10 Mb, or >10 Mb). DUC, Korean Duroc; KNP, Korean native pig; F1, DUC × KNP; F2, F1 × DUC; WRH, Woori-Heukdon (F1 × F2).

The mean number and size of discovered ROH per pig within each population are described in Table 2. Among the five pig populations, the largest mean size of ROH was observed in KNP (14,205.2 ± 17,912.2 kb; N = 65.1), and followed by WRH (7,169.8 ± 8,695.3 kb; N = 58.7), DUC (6,117.5 ± 6,418.3 kb; 65.9), F2 (3,973.9 ± 3,836.9 kb; N = 61.2) and F1 (2,003.5 ± 1,014.0 kb; N = 39.8). Both parental breeds (DUC and KNP) showed to have larger mean size and number of ROH than F1 and F2 crossbred populations, whereas mean length and ROH number were higher in WRH than that of DUC. The ROH length for parental breeds ranged from 1,266 kb (SSC12) to 186,522 kb (SSC1) in DUC and from 2,060 kb (SSC2) to 262,078 kb (SSC1) in KNP. For crossbred populations, the ROH length ranged from 1,014 kb (SSC1) to 9,527 kb (SSC6) in F1, 1,041 kb (SSC12) to 58,419 kb (SSC1) in F2, and 1,247 kb (SSC2) to 152,760 kb (SSC1) in WRH. As shown in Figure 4 and Supplementary Table 3, the proportion of short ROH (1–3 Mb) within each population was the highest in F1 (90.2%; N = 395), followed by F2 (54.9%; N = 4,839), WRH (26.5%; N = 9,691), DUC (24.4%; N = 17,075), and KNP (1.6%; N = 203). The proportion of medium ROH (3–10 Mb) was highest in DUC (62.9%; N = 44,038) and lowest in F1 (9.8%; N = 43), long ROH (>10 Mb) were most frequently observed in KNP (41.9%; N = 5,436), and no long ROH were observed in F1. We also observed parental ROH regions in crossbred populations using the coordinates of concatenated ROH regions that were generated by joining overlapped (at least 1bp) ROH segments within each of the populations (DUC, KNP, F1, F2, and WRH) (Supplementary Table 4). Most of ROH regions (>98.9%) in crossbreds were derived from the shared ROH region (approximately 2,218 Mb) between DUC and KNP.

**TABLE 2:** Summary of discovered ROH in five pig populations.

Pop	ROH length (kb)			No. of ROH per individual			F_ROH_ ± SD
	Mean	Min^a^	Max^b^	Mean	Min^c^	Max^d^	
DUC	6,117.5 ± 6,418.3	1,266.3 (SSC12)	186,521.5 (SSC1)	65.9 ± 8.0	4	90	0.178 ± 0.035
KNP	14,205.2 ± 17,912.2	2,060.1 (SSC2)	262,078.0 (SSC1)	65.1 ± 5.5	47	80	0.409 ± 0.046
F1	2,003.5 ± 969.0	1,014.0 (SSC1)	9,527.0 (SSC6)	39.8 ± 3.3	35	44	0.035 ± 0.003
F2	3,973.9 ± 3,836.9	1,040.8 (SSC12)	58,419.8 (SSC1)	61.2 ± 9.9	31	87	0.107 ± 0.025
WRH	7,169.8 ± 8,695.3	1,247.0 (SSC2)	152,760.6 (SSC1)	58.7 ± 9.3	7	89	0.186 ± 0.055

a Minimum length (kb) of ROH, within each population.

b Maximum length (kb) of ROH, within each population.

c Minimum number of ROHs, per individual.

d Maximum number of ROHs, per individual. Pop, population; No., number; F_ROH_, inbreeding coefficient based on runs of homozygosity; SD, standard deviation; DUC, Korean Duroc; KNP, Korean native pig; F1, DUC × KNP; F2, F1 × DUC; WRH, Woori-Heukdon (F1 × F2).

The inbreeding coefficient among all populations was further calculated based on ROH (F_ROH_), which was defined by McQuillan et al. (2008) (Table 2). This result showed that the highest F_ROH_ value was observed in KNP (0.409) followed by WRH (0.186), DUC (0.178), F2 (0.107), and F1 (0.035), which is the same order as mean ROH length. The correlation between F_HOM_ and F_ROH_ for each population was high in most populations, including DUC (0.84), KNP (0.83), F2 (0.86), and WRH (0.88); however, F1 had a correlation of 0.10 (Supplementary Figure 2). Because F_HOM_ is influenced by allele frequency and sampling (Zhang et al., 2015) but the level of homozygosity in F_ROH_ is independent of allele frequencies, the low correlation between F_HOM_ and F_ROH_ in F1 might be caused by sampling bias derived from the low sample size of F1 (N = 11) (Dixit et al., 2020). Therefore, we focused on F_ROH_ to assess the degree of inbreeding, which is also better for detecting both common and rare variants than F_HOM_ (Wang et al., 2019).

### ROH Islands and Gene Annotation

**FIGURE 5:**
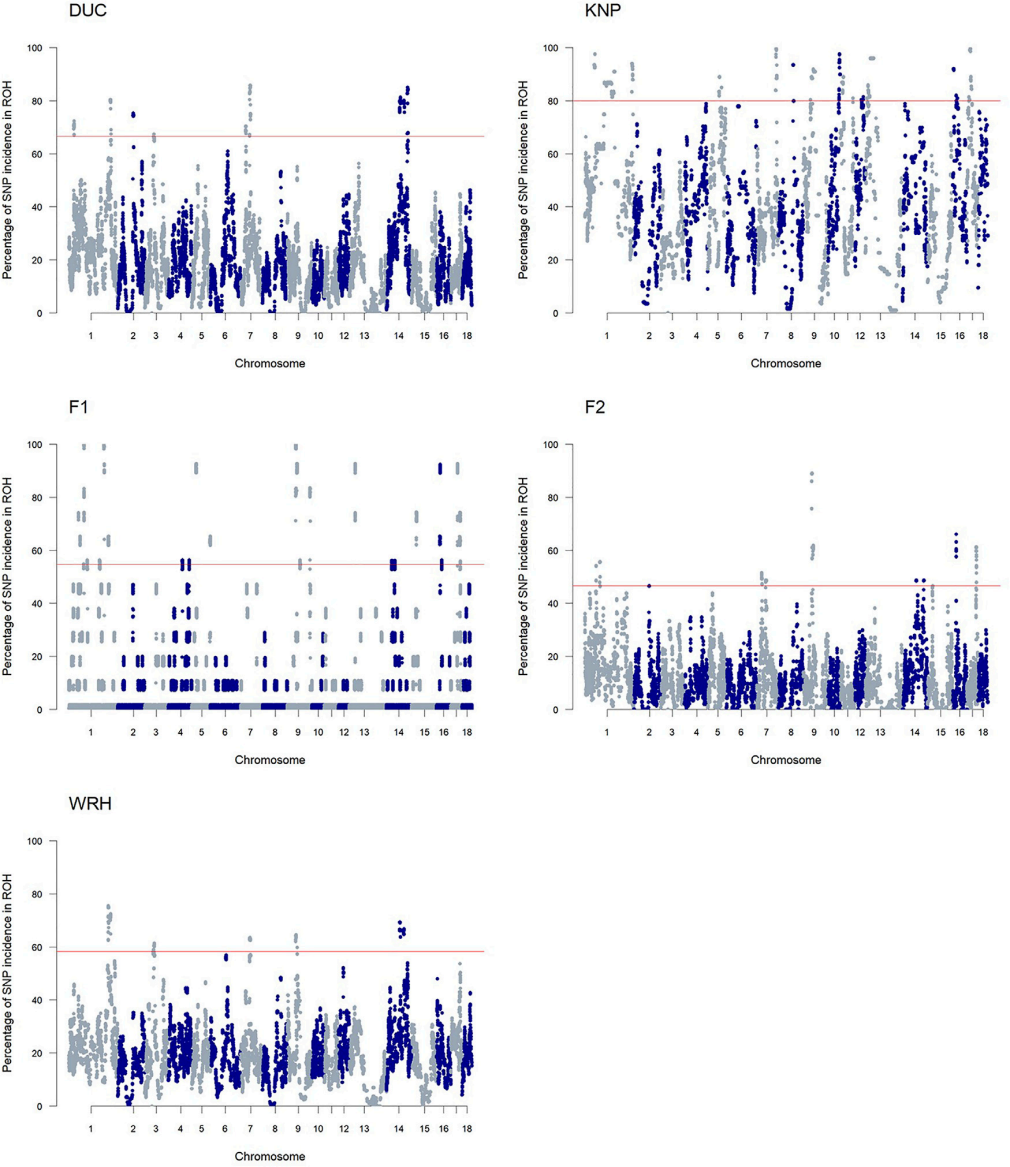
Manhattan plot of SNP frequency in ROH islands. The horizontal line (red) indicates the threshold for ROH islands for each population. DUC, Korean Duroc; KNP, Korean native pig; F1, DUC × KNP; F2, F1 × DUC; WRH, Woori-Heukdon (F1 × F2).

As shown in Figure 5 and Supplementary Table 5, the ROH islands, which were defined as regions where SNPs in ROH had *p*-values higher than a threshold for each population, were observed for parental (DUC and KNP) and crossbred (F1, F2, and WRH) populations. We identified a total of 365 ROH islands of 1-Mb bins throughout the autosomal regions (Supplementary Table 6). DUC had ROH islands on SSC1–3, SSC7, and SSC14, whereas KNP had ROH islands on 11 autosomes, excluding SSC2, SSC3, SSC4, SSC6, SSC14, SSC15, and SSC18. Crossbred populations, especially F1 and F2, had similar occurrence patterns of ROH islands, with ROH islands on SSC1, SSC9, and SSC14–17 in both populations. Additional islands only in F1 were on SSC4, SSC5, SSC13, and SSC18, and those only in F2 were on SSC2 and SSC7. For WRH, SSC1, SSC3, SSC7, SSC9, and SSC14 had ROH islands. For all 1-Mb bin of ROH islands, we annotated 2,165 genes and 11 QTL information and are listed in Supplementary Table 6.

## Discussion

To investigate the population structure of DUC, KNP, and their crossbred populations (F1, F2, and WRH), we conducted PCA and ADMIXTURE analyses (Figure 2 and Figure 3). The most distant genetic relationship was observed between DUC and KNP based on PCA (Figure 2). In addition, KNP separated from DUC at K = 2 and had 100% distinct ancestry (Figure 3; Supplementary Table 2). The results are consistent with those of previous reports that showed clear genetic difference based on population structure and F_ST_ analyses (Edea et al., 2014; Kim Y.-M. et al., 2020; Lee et al., 2020). For the crossbred populations (F1, F2, and WRH) used in this study, all three populations were located between KNP and DUC in the PCA (Figure 2). In particular, F1 was located in the middle of KNP and DUC, which was also shown in other F1 populations generated from purebred parental pig breeds (Grossi et al., 2017) and cattle (Gobena et al., 2018). The ADMIXTURE analysis of K = 2 also revealed that F1 had 48.9% DUC and 51.1% KNP ancestry (Supplementary Table 2). The genetic distance of F2 and WRH crossbred populations was closer to DUC than that to KNP, which can be explained by the higher genetic composition of DUC than that of KNP in the crossing scheme of F2 and WRH (Figure 1). At K = 2 in the ADMIXTURE analysis, F2 had approximately 74.1% DUC and 25.9% of KNP ancestry. In addition, WRH had approximately 65.9% DUC and 34.1% KNP ancestry. This better fits the theoretical genomic composition of DUC (62.5%) and KNP (37.5%) than a previous study that reported the genomic composition of DUC (74.8%) and KNP (25.2%) in WRH (Kim Y.-M. et al., 2020). This study only used parental breeds (DUC and KNP) and crossbred populations (F1 and F2) that were used to develop WRH, even though the previous study used additional Chinese and commercial breeds. Therefore, we inferred that a better estimation of ancestry for WRH was obtained in this study.

However, WRH was shown to have a somewhat dispersed cluster in PCA (Figure 2A). This could be explained by newly generated breeding group of WRH using parental breeds (DUC and KNP) and crossbred populations (F1 and F2). The first national project to develop a Korean composite pig breed (WRH) started in 2008 by generating F1 and F2 populations. Subsequently, the first founder population of WRH was developed in 2010. Since then, the initial founder stock of WRH was used for breeding projects as a closed population until 2018. Because of difficulties maintaining a sufficiently large effective population size with a limited population size, they were subject to inbreeding (Dickerson, 1973). To decrease the level of inbreeding of this population, a recent project was initiated to construct new breeding group of WRH population since 2018. Therefore, recent introduction of new breeding group to the previous one might cause the somewhat widely distributed cluster shown in population structure analyses.

The effect of heterosis is difficult to quantify; however, heterozygosity can be used as an indicator of heterosis (Iversen et al., 2019). Genetic diversity levels were assessed by mean expected heterozygosity rate of five pig populations. The expected heterozygosity gradually increased according to crossing stages used in this study (F1, F2, and WRH) (Table 1), and those values were higher than in the parental breeds. Similar to this result, a previous study reported that crossbred pigs generated between Dutch Landrace and Dutch Large White had higher heterozygosity levels compared with their parental breeds (Iversen et al., 2019). Among the five pig populations, the lowest degree of genetic diversity was observed in KNP, and this result is concordant with those of previous studies that reported the genetic diversity of KNP relative to most of studied other indigenous and commercial pig breeds using genomic datasets (Kim Y.-M. et al., 2020; Lee et al., 2020). Other studies that assessed genetic diversity of indigenous pigs from other countries also revealed loss of genetic diversity in indigenous pig breeds due to conservation status (Diao et al., 2019; Munoz et al., 2019). Although KNP has not undergone systematic artificial selection during conservation breeding, such loss of genetic diversity could be explained by a small effective population size and the founder effect or a population bottleneck (Munoz et al., 2019). In terms of breeding history, the KNP restoration project started in 1988 using only nine individuals (four males and five females) as a founder population (Kim et al., 2016); simultaneously, the preservation program was conducted as a closed population (Kim Y.-M. et al., 2020). We suggest that those characteristics of the breeding system might have caused a high level of inbreeding, as KNP had the greatest F_ROH_ value (.409) (Table 2).

In ROH analyses, we found a clear difference in ROH patterns between parental breeds and their crossbred populations (F1 and F2). Among parental breeds, DUC had 24.4% short and 12.7% long ROH segments (Figure 4). Additionally, KNP had abundant medium (56.5%) and long (41.9%) ROH segments. The short ROH segments may indicate evolutionary events from old inbreeding or selection, whereas long ROH may reflect recent inbreeding (Keller et al., 2011; Mastrangelo et al., 2017; Xu L. et al., 2019). DUC was shown to have more old inbreeding than recent inbreeding or selection pressure due to intensive selection programs (Bovo et al., 2020), whereas KNP underwent recent inbreeding of small populations (Valluzzi et al., 2021); this was also confirmed by the genetic diversity and inbreeding results in this study. Of the crossbred populations, F1 has the lowest number of total ROH segments (N = 438) among populations used in this study (Supplementary Table 3), and most of them are short ROHs (90.2%) and no long ROHs. These ROH patterns in F1 also caused the short length of concatenated ROH regions compared to F2 and WRH (Supplementary Table 4), which might be caused by the increase of heterozygous SNPs in the genome due to the hybridization of parental breeds. This is also supported by the highest value of observed heterozygosity in F1 (Table 1). However, a caution is required to interpret the ROH results derived from F1, because there was highly likely to be an underestimation in ROH numbers and size due to a low sample size (N = 11). Furthermore, we identified a total of 8,810 and 36,635 ROHs for F2 and WRH, respectively (Supplementary Table 3). In both populations, short ROH segments gradually decreased, whereas medium and long ROH segments increased; these changes also increased the inbreeding coefficient based on ROH (Table 2) and the length of concatenated ROH region (Supplementary Table 4) in those populations. A previous study also revealed that, in a 3-way crossbred population [(Pietrain × Large White) × Duroc)], G0 had smaller homozygous segments than their parental populations, and ROH size was increased in the G1 population, which were the offspring of G0 (Ganteil et al., 2020). Consequently, WRH showed a similar proportion of size-classified ROH to DUC, but WRH had a higher proportion of long ROH than DUC; this indicated recent inbreeding events, as discussed earlier in the population structure analyses. We also observed parental ROHs in crossbred populations using coordinates of concatenated ROH regions for each of the populations (Supplementary Table 4). Most ROH regions in crossbreds (>98.8%) were considered to be from the ROH regions shared between parental breeds (DUC and KNP). In particular, the proportions of the ROH regions over the initial total length of ROH region overlapped between parental breeds were 91.55 and 99.55% for F2 and WRH, respectively (data not shown). We suggest that such increased ROH regions in F2 and WRH might be in part due to the inbreeding.

The discovery of ROH islands revealed numerous homozygous regions over five pig populations used in this study (Figure 5; Supplementary Table 6). Furthermore, we found ROH islands in the parental breeds that were shared with their crossbred populations on SSC1–3, SSC7, SSC9, SSC13, SSC14, and SSC16, which indicated inheritance of homozygous regions (Supplementary Table 5). Most of those regions in crossbred populations were shorter than those in parental populations, which might be explained by ROH degeneration due to an increase of heterozygous SNPs or recombination (Bosse et al., 2012). The breakage of the ROHs is supported by the proportion of parental ROH regions in crossbred populations (Supplementary Table 4), which indicated that most of ROH regions from the crossbreds (F1, F2 and WRH) belongs to shared ROH regions between parental breeds. Previous studies also revealed ROH persistence in several crossbred pigs: Landrace × Large White (Landrace × Large White) × Duroc, and (Pietrain × Large White) × Duroc (Howard et al., 2016; Gomez-Raya et al., 2019; Ganteil et al., 2020).

In this study, we annotated genes to ROH islands to identify inheritance of homozygous regions that might be potential selection signatures from parental breeds to WRH, which is the last stage of the crossbreeding scheme. First, we found some candidate genes in ROH islands that overlapped between KNP and WRH. We found an ROH island on SSC9 (49–50 Mb) harboring the Cytotoxic And Regulatory T Cell Molecule (*CRTAM*) gene. This gene was reported to have an association with adaptive immune response in cattle (Ben-Jemaa et al., 2021). As KNP is an indigenous pig breed that has been adapted to the local environment of South Korea for a long period, we suggest that *CRTAM* might be a candidate gene that is associated with local adaptation of KNP and WRH populations. At the same location (SSC9; 49–50 Mb) of a ROH island, we found the Heat Shock Protein Family A Member 8 (*HSPA8*) gene, which is also known as Hsp70. *HSPA8* is known to be associated with pork tenderness because this gene was down-regulated in tender samples (Hamill et al., 2012).

We also retrieved ROH islands that were shared between DUC and WRH. We found the ADAMTS Like 3 (*ADAMTSL3*) gene at 52–53 Mb on SSC7. *ADAMTSL3* is a member of the ADAMTS superfamily of proteins, and this gene was previously reported as a candidate gene for body length in Large White pigs (Li et al., 2017) and height in humans (Weedon et al., 2008). In addition, we located the Cytoplasmic Polyadenylation Element Binding Protein 1 (*CPEB1*) gene at 52–53 Mb on SSC7. The *CPEB1* is an RNA-binding protein that regulates mRNA translation by controlling the poly(A) tail length (Nagaoka et al., 2016). *CPEB1* was reported to increase the rate of meiotic resumption and expression of cyclin B when mRNA was injected into immature oocytes (Nishimura et al., 2010). The purpose of using DUC in the crossing scheme includes complementing the body size and reproductive traits of KNP; thus, we suggest that *ADAMTSL3* and *CPEB1*, which are located in ROH islands of DUC and WRH, might be associated with production and reproductive traits in both populations.

## Conclusion

This study has shown genomic characteristics of crossbred pig populations derived from Korean Duroc and Korean native pigs as founder breeds. Population structure analysis showed genetic influence of founder breeds to crossbred populations. We also observed that WRH had two distinct subgroups due to newly introduced breeding group. For crossbred populations, the genetic diversity was gradually increased according to their crossbreeding stage (F1, F2 and WRH). In ROH analyses, short ROHs were decreased, while medium and long ROHs were increased from F1 to WRH, suggesting that recent inbreeding is ongoing in WRH. In this study, there is a partial limitation to conclude on F1 due to its small samples size (N = 11). Furthermore, we identified shared ROH islands which contain candidate genes (*CRTAM*, *HSPA8*, *ADAMTSL3* and *CPEB1* genes) between WRH and founder breeds, suggesting inheritance of homozygous region that might be potential signatures of selection.

## Data Availability

The SNP dataset for DUC used in this study is deposited in FigShare, https://doi.org/10.6084/m9.figshare.18318584.v1. The datasets for other populations are available from the corresponding author upon request.

## References

[B1] Alexander D. H. Lange K. (2011). Enhancements to the ADMIXTURE Algorithm for Individual Ancestry Estimation. BMC Bioinformatics 12, 246. 10.1186/1471-2105-12-246 21682921 PMC3146885

[B2] Baas T. J. Christian L. L. Rothschild M. F. (1992). Heterosis and Recombination Effects in Hampshire and Landrace Swine: I. Maternal Traits. J. Anim. Sci. 70 (1), 89–98. 10.2527/1992.70189x 1582925

[B3] Ben-Jemaa S. Senczuk G. Ciani E. Ciampolini R. Catillo G. Boussaha M. (2021). Genome-Wide Analysis Reveals Selection Signatures Involved in Meat Traits and Local Adaptation in Semi-feral Maremmana Cattle. Front. Genet. 12, 675569. 10.3389/fgene.2021.675569 33995500 PMC8113768

[B4] Bosse M. Megens H.-J. Madsen O. Paudel Y. Frantz L. A. F. Schook L. B. (2012). Regions of Homozygosity in the Porcine Genome: Consequence of Demography and the Recombination Landscape. Plos Genet. 8 (11), e1003100. 10.1371/journal.pgen.1003100 23209444 PMC3510040

[B5] Bovo S. Ribani A. Muñoz M. Alves E. Araujo J. P. Bozzi R. (2020). Whole-genome Sequencing of European Autochthonous and Commercial Pig Breeds Allows the Detection of Signatures of Selection for Adaptation of Genetic Resources to Different Breeding and Production Systems. Genet. Sel Evol. 52 (1), 33. 10.1186/s12711-020-00553-7 32591011 PMC7318759

[B6] Broman K. W. Weber J. L. (1999). Long Homozygous Chromosomal Segments in Reference Families from the Centre d'Étude du Polymorphisme Humain. Am. J. Hum. Genet. 65 (6), 1493–1500. 10.1086/302661 10577902 PMC1288359

[B7] Cassady J. P. Young L. D. Leymaster K. A. (2002a). Heterosis and Recombination Effects on Pig Growth and Carcass Traits. J. Anim. Sci. 80 (9), 2286–2302. 10.2527/2002.8092286x 12350006

[B8] Cassady J. P. Young L. D. Leymaster K. A. (2002b). Heterosis and Recombination Effects on Pig Reproductive Traits. J. Anim. Sci. 80 (9), 2303–2315. 10.2527/2002.8092303x 12350007

[B9] Chang C. C. Chow C. C. Tellier L. C. Vattikuti S. Purcell S. M. Lee J. J. (2015). Second-generation PLINK: Rising to the challenge of Larger and Richer Datasets. GigaSci 4, 7. 10.1186/s13742-015-0047-8 PMC4342193 25722852

[B10] Choi Y.-S. Lee J.-K. Jung J.-T. Jung Y.-C. Jung J.-H. Jung M.-O. (2016). Comparison of Meat Quality and Fatty Acid Composition of Longissimus Muscles from Purebred Pigs and Three-Way Crossbred LYD Pigs. Korean J. Food Sci. Anim. Resour. 36 (5), 689–696. 10.5851/kosfa.2016.36.5.689 27857546 PMC5112433

[B11] Diao S. Huang S. Xu Z. Ye S. Yuan X. Chen Z. (2019). Genetic Diversity of Indigenous Pigs from South China Area Revealed by SNP Array. Animals 9 (6), 361. 10.3390/ani9060361 PMC6616596 31208134

[B12] Dickerson G. E. (1973). Inbreeding and Heterosis in Animals. J. Anim. Sci. 1973 (Symposium), 54–77. 10.1093/ansci/1973.Symposium.54

[B13] Dixit S. P. Singh S. Ganguly I. Bhatia A. K. Sharma A. Kumar N. A. (2020). Genome-Wide Runs of Homozygosity Revealed Selection Signatures in Bos indicus. Front. Genet. 11, 92. 10.3389/fgene.2020.00092 32153647 PMC7046685

[B14] Dzomba E. F. Chimonyo M. Pierneef R. Muchadeyi F. C. (2021). Runs of Homozygosity Analysis of South African Sheep Breeds from Various Production Systems Investigated Using OvineSNP50k Data. BMC Genomics 22 (1), 7. 10.1186/s12864-020-07314-2 33407115 PMC7788743

[B15] Edea Z. Kim S.-W. Lee K.-T. Kim T. H. Kim K.-S. (2014). Genetic Structure of and Evidence for Admixture between Western and Korean Native Pig Breeds Revealed by Single Nucleotide Polymorphisms. Asian Australas. J. Anim. Sci. 27 (9), 1263–1269. 10.5713/ajas.2014.14096 25178369 PMC4150192

[B16] Falconer D. S. Mackay T. F. C. (1996). An Introduction to Quantitative Genetics. Essex, UK: Longman Group.

[B17] Ferenčaković M. Hamzić E. Gredler B. Solberg T. R. Klemetsdal G. Curik I. (2013). Estimates of Autozygosity Derived from Runs of Homozygosity: Empirical Evidence from Selected Cattle Populations. J. Anim. Breed. Genet. 130 (4), 286–293. 10.1111/jbg.12012 23855630

[B18] Ganteil A. Rodriguez-Ramilo S. T. Ligonesche B. Larzul C. (2020). Characterization of Autozygosity in Pigs in Three-Way Crossbreeding. Front. Genet. 11, 584556. 10.3389/fgene.2020.584556 33584790 PMC7876413

[B19] Gobena M. Elzo M. A. Mateescu R. G. (2018). Population Structure and Genomic Breed Composition in an Angus-Brahman Crossbred Cattle Population. Front. Genet. 9, 90. 10.3389/fgene.2018.00090 29636769 PMC5881247

[B20] Gomez-Raya L. Rauw W. M. Dunkelberger J. R. Dekkers J. C. M. (2019). Autozygosity and Genetic Differentiation of Landrace and Large White Pigs as Revealed by the Genetic Analyses of Crossbreds. Front. Genet. 10, 739. 10.3389/fgene.2019.00739 31543894 PMC6739446

[B21] Gorssen W. Meyermans R. Buys N. Janssens S. (2020). SNP Genotypes Reveal Breed Substructure, Selection Signatures and Highly Inbred Regions in Piétrain Pigs. Anim. Genet. 51 (1), 32–42. 10.1111/age.12888 31809557 PMC7003864

[B57] Gorssen W. Meyermans R. Janssens S. Buys N. (2021). A Publicly Available Repository of ROH Islands Reveals Signatures of Selection in Different Livestock and Pet Species. Genetics Selection Evolution 53 (1), 1–10. 10.1186/s12711-020-00599-7 PMC7784028 33397285

[B22] Grossi D. A. Jafarikia M. Brito L. F. Buzanskas M. E. Sargolzaei M. Schenkel F. S. (2017). Genetic Diversity, Extent of Linkage Disequilibrium and Persistence of Gametic Phase in Canadian Pigs. BMC Genet. 18 (1), 6. 10.1186/s12863-017-0473-y 28109261 PMC5251314

[B23] Hamill R. M. McBryan J. McGee C. Mullen A. M. Sweeney T. Talbot A. (2012). Functional Analysis of Muscle Gene Expression Profiles Associated with Tenderness and Intramuscular Fat Content in Pork. Meat Sci. 92 (4), 440–450. 10.1016/j.meatsci.2012.05.007 22688437

[B24] Howard J. T. Tiezzi F. Huang Y. Gray K. A. Maltecca C. (2016). Characterization and Management of Long Runs of Homozygosity in Parental Nucleus Lines and Their Associated Crossbred Progeny. Genet. Sel Evol. 48 (1), 91. 10.1186/s12711-016-0269-y 27884108 PMC5123398

[B25] Hur S. J. Jeong T. C. Kim G. D. Jeong J. Y. Cho I. C. Lim H. T. (2013). Comparison of Live Performance and Meat Quality Parameter of Cross Bred (Korean Native Black Pig and Landrace) Pigs with Different Coat Colors. Asian Australas. J. Anim. Sci. 26 (7), 1047–1053. 10.5713/ajas.2013.13005 25049884 PMC4093497

[B26] Iversen M. W. Nordbø Ø. Gjerlaug-Enger E. Grindflek E. Lopes M. S. Meuwissen T. (2019). Effects of Heterozygosity on Performance of Purebred and Crossbred Pigs. Genet. Sel Evol. 51 (1), 8. 10.1186/s12711-019-0450-1 30819106 PMC6396501

[B27] Jin S. Kim I. Hur S. Kim S. Jeong K. (2006). The Influence of Pig Breeds on Qualities of Loin. J. Anim. Sci. Techn 48 (5), 747–758. 10.5187/JAST.2006.48.5.747

[B28] Johnson R. K. (1981). Crossbreeding in Swine: Experimental Results. J. Anim. Sci. 52 (4), 906–923. 10.2527/jas1981.524906x

[B29] Keller M. C. Visscher P. M. Goddard M. E. (2011). Quantification of Inbreeding Due to Distant Ancestors and its Detection Using Dense Single Nucleotide Polymorphism Data. Genetics 189 (1), 237–249. 10.1534/genetics.111.130922 21705750 PMC3176119

[B30] Kim S. G. Bae H. H. Son J. Y. Shin J. S. Ha G. H. (2020a). The Trends of Consumption of Pork Meat in Korean 2019 NIAS, RDA, Republic of Korea. Wanju, South Korea: NIAS.

[B31] Kim Y.-M. Seong H.-S. Lee J.-J. Son D.-H. Kim J.-S. Sa S.-J. (2020b). Genome-wide Investigation of a Korean Synthetic Breed, Woori-Heukdon Using the Illumina PorcineSNP60K BeadChip. Genes Genom 42 (12), 1443–1453. 10.1007/s13258-020-01008-5 33145727

[B32] Kim Y. M. Cho G. H. Park J. C. Kim D. W. Hong J. K. Sa S. J. (2016). Preparing Method of Synthetic Pig by a Cross between Duroc and Korean Native Pig, and Synthetic Pig Using the Same. Republic of Korea patent application.

[B33] Lee S. H. Seo D. W. Cho E. S. Choi B. H. Kim Y. M. Hong J. K. (2020). Genetic Diversity and Ancestral Study for Korean Native Pigs Using 60K SNP Chip. Animals 10 (5), 760. 10.3390/ani10050760 PMC7277343 32349346

[B34] Lencz T. Lambert C. DeRosse P. Burdick K. E. Morgan T. V. Kane J. M. (2007). Runs of Homozygosity Reveal Highly Penetrant Recessive Loci in Schizophrenia. Proc. Natl. Acad. Sci. 104 (50), 19942–19947. 10.1073/pnas.0710021104 18077426 PMC2148402

[B35] Li X. Yang S. Dong K. Tang Z. Li K. Fan B. (2017). Identification of Positive Selection Signatures in Pigs by Comparing Linkage Disequilibrium Variances. Anim. Genet. 48 (5), 600–605. 10.1111/age.12574 28736898

[B36] Lopes M. S. Silva F. F. Harlizius B. Duijvesteijn N. Lopes P. S. Guimarães S. E. (2013). Improved Estimation of Inbreeding and Kinship in Pigs Using Optimized SNP Panels. BMC Genet. 14, 92. 10.1186/1471-2156-14-92 24063757 PMC3849284

[B37] Marras G. Gaspa G. Sorbolini S. Dimauro C. Ajmone-Marsan P. Valentini A. (2015). Analysis of Runs of Homozygosity and Their Relationship with Inbreeding in Five Cattle Breeds Farmed in Italy. Anim. Genet. 46 (2), 110–121. 10.1111/age.12259 25530322

[B38] Mastrangelo S. Tolone M. Sardina M. T. Sottile G. Sutera A. M. Di Gerlando R. (2017). Genome-wide scan for runs of homozygosity identifies potential candidate genes associated with local adaptation in Valle del Belice sheep. Genet. Sel Evol. 49 (1), 84. 10.1186/s12711-017-0360-z 29137622 PMC5684758

[B58] McQuillan R. Leutenegger A.-L. Abdel-Rahman R. Franklin C. S. Pericic M. Barac-Lauc L. (2021). Runs of Homozygosity in European Populations. The American Journal of Human Genetics 83 (3), 359–372. 10.1016/j.ajhg.2008.08.007 PMC2556426 18760389

[B39] Meyermans R. Gorssen W. Buys N. Janssens S. (2020). How to Study Runs of Homozygosity Using PLINK? A Guide for Analyzing Medium Density SNP Data in Livestock and Pet Species. BMC Genomics 21 (1), 94. 10.1186/s12864-020-6463-x 31996125 PMC6990544

[B40] Muñoz M. Bozzi R. García-Casco J. Núñez Y. Ribani A. Franci O. (2019). Genomic Diversity, Linkage Disequilibrium and Selection Signatures in European Local Pig Breeds Assessed with a High Density SNP Chip. Sci. Rep. 9 (1), 13546. 10.1038/s41598-019-49830-6 31537860 PMC6753209

[B41] Nagaoka K. Fujii K. Zhang H. Usuda K. Watanabe G. Ivshina M. (2016). CPEB1 Mediates Epithelial-To-Mesenchyme Transition and Breast Cancer Metastasis. Oncogene 35 (22), 2893–2901. 10.1038/onc.2015.350 26411364 PMC4809797

[B42] Nishimura Y. Kano K. Naito K. (2010). Porcine CPEB1 Is Involved in Cyclin B Translation and Meiotic Resumption in Porcine Oocytes. Anim. Sci. J. 81 (4), 444–452. 10.1111/j.1740-0929.2010.00755.x 20662813

[B43] Park B. Y. Kim N. K. Lee C. S. Hwang I. H. (2007). Effect of Fiber Type on Postmortem Proteolysis in Longissimus Muscle of Landrace and Korean Native Black Pigs. Meat Sci. 77 (4), 482–491. 10.1016/j.meatsci.2007.04.022 22061932

[B44] Peripolli E. Metzger J. de Lemos M. V. A. Stafuzza N. B. Kluska S. Olivieri B. F. (2018). Autozygosity Islands and ROH Patterns in Nellore Lineages: Evidence of Selection for Functionally Important Traits. BMC Genomics 19 (1), 680. 10.1186/s12864-018-5060-8 30223795 PMC6142381

[B45] Purfield D. C. Berry D. P. McParland S. Bradley D. G. (2012). Runs of Homozygosity and Population History in Cattle. BMC Genet. 13, 70. 10.1186/1471-2156-13-70 22888858 PMC3502433

[B46] Purfield D. C. McParland S. Wall E. Berry D. P. (2017). The Distribution of Runs of Homozygosity and Selection Signatures in Six Commercial Meat Sheep Breeds. PLoS One 12 (5), e0176780. 10.1371/journal.pone.0176780 28463982 PMC5413029

[B47] Schachler K. Distl O. Metzger J. (2020). Tracing Selection Signatures in the Pig Genome Gives Evidence for Selective Pressures on a Unique Curly Hair Phenotype in Mangalitza. Sci. Rep. 10 (1), 22142. 10.1038/s41598-020-79037-z 33335158 PMC7747725

[B48] Schiavo G. Bovo S. Bertolini F. Dall'Olio S. Costa L. N. Tinarelli S. (2020). Runs of Homozygosity Islands in Italian Cosmopolitan and Autochthonous Pig Breeds Identify Selection Signatures in the Porcine Genome. Livestock Sci. 240, 104219. 10.1016/j.livsci.2020.104219

[B49] Schiavo G. Bovo S. Muñoz M. Ribani A. Alves E. Araújo J. P. (2021). Runs of Homozygosity Provide a Genome Landscape Picture of Inbreeding and Genetic History of European Autochthonous and Commercial Pig Breeds. Anim. Genet. 52 (2), 155–170. 10.1111/age.13045 33544919

[B50] Valluzzi C. Rando A. Macciotta N. P. P. Gaspa G. Di Gregorio P. (2021). The Nero Lucano Pig Breed: Recovery and Variability. Animals 11 (5), 1331. 10.3390/ani11051331 34067067 PMC8150585

[B51] Wang L. Mu Y. Xu L. Li K. Han J. Wu T. (2019). Genomic Analysis Reveals Specific Patterns of Homozygosity and Heterozygosity in Inbred Pigs. Animals 9 (6), 314. 10.3390/ani9060314 PMC6617223 31159442

[B52] Weedon M. N. Lango H. Lango H. Lindgren C. M. Wallace C. Evans D. M. (2008). Genome-wide Association Analysis Identifies 20 Loci that Influence Adult Height. Nat. Genet. 40 (5), 575–583. 10.1038/ng.121 18391952 PMC2681221

[B53] Wu F. Sun H. Lu S. Gou X. Yan D. Xu Z. (2020). Genetic Diversity and Selection Signatures within Diannan Small-Ear Pigs Revealed by Next-Generation Sequencing. Front. Genet. 11, 733. 10.3389/fgene.2020.00733 32849777 PMC7406676

[B54] Xu L. Zhao G. Yang L. Zhu B. Chen Y. Zhang L. (2019a). Genomic Patterns of Homozygosity in Chinese Local Cattle. Sci. Rep. 9 (1), 16977. 10.1038/s41598-019-53274-3 31740716 PMC6861314

[B55] Xu Z. Sun H. Zhang Z. Zhao Q. Olasege B. S. Li Q. (2019b). Assessment of Autozygosity Derived from Runs of Homozygosity in Jinhua Pigs Disclosed by Sequencing Data. Front. Genet. 10, 274. 10.3389/fgene.2019.00274 30984245 PMC6448551

[B56] Zhang Q. Guldbrandtsen B. Bosse M. Lund M. S. Sahana G. (2015). Runs of Homozygosity and Distribution of Functional Variants in the Cattle Genome. BMC Genomics 16, 542. 10.1186/s12864-015-1715-x 26198692 PMC4508970

