# Supplementary material for: Genome-Wide Assessment of a Korean Composite Pig Breed, Woori-Heukdon
Source: Front Genet. 2022 Feb 2;13:779152. doi: 10.3389/fgene.2022.779152 (PMC8847790; doi:10.3389/fgene.2022.779152)
Supplement: Supplementary file 1 [file DataSheet1.PDF]

Supplementary Table 1. The difference of SNP distribution for each Sus scrofa chromosome (SSC) between S.scrofa 10.2 and 11.1 genome assemblies.

SSC	Based on S.scrofa 10.2		Based on S.scrofa 11.1	
	Number of SNPs	Mean SNP interval (kb)	Number of SNPs	Mean SNP interval (kb)
0	7,849	0	1,091	0
1	6,512	48.4	7,183	38.2
2	3,392	47.9	3,767	40.3
3	2,805	51.6	3,263	40.6
4	3,551	40.4	3,902	33.5
5	2,361	47.1	2,740	38.1
6	3,216	49.1	4,122	41.5
7	3,348	40.2	3,610	33.7
8	2,777	53.2	3,178	43.7
9	3,213	47.8	3,528	39.5
10	1,796	43.9	1,954	35.3
11	1,890	46.4	2,151	36.7
12	1,562	40.6	1,803	34.1
13	4,090	53.3	4,604	45.2
14	3,906	39.3	4,267	33.2
15	2,906	54.2	3,303	42.5
16	1,877	46.3	2,086	38.2
17	1,720	40.4	1,916	33.1
18	1,336	45.6	1,487	37.6
X	1,449	99.4	1,426	88.1
Y	9	156.7	58	734.6
XY	-	-	126	50.8
total	61,565	42.1	61,565	39.6

Supplementary Table 2. Proportion of inferred ancestry at K = 2.

Pop	Inferred population (%)	
	K = 2	
	1	2
DUC	99.3	0.7
KNP	0.0	100
F1	48.9	51.1
F2	74.1	25.9
WRH	65.9	34.1

Supplementary Table 3. Overall number of ROHs identified among 5 pig populations according to size-classification (1–3 Mb, 3–10 Mb, or >10 Mb).

Pop	No. of ROHs			
	1-3 Mb	3-10 Mb	>10 Mb	Total
DUC	17,075	44,038	8,921	70,034
KNP	203	7,313	5,436	12,952
F1	395	43	0	438
F2	4,839	3,477	494	8,810
WRH	9,691	20,557	6,387	36,635

Pop, population; No., number; DUC, Korean Duroc; KNP, Korean native pig; F1, DUC × KNP; F2, F1 × DUC; WRH, Woori-Heukdon (F1 × F2).

Supplementary Table 4. The classification of parental ROH region in crossbred populations (F1, F2 and WRH) using coordinates of concatenated ROH region.

**From**	**Length of ROH region (kb)**		
	**F1**	**F2**	**WRH**
DUC-R[1]	-	19	1,046
KNP-R[2]	-	1,026	24,572
DK-R[3]	304,847	2,029,402	2,207,976
Novel-R[4]	-	-	-
Total	304,847	2,030,447	2,233,594

[1], ROH region derived from only DUC; [2], ROH region derived from only KNP; [3], ROH region derived from shared ROH region between DUC and KNP; [4], Novel ROH region not included in DUC or KNP; DUC, Korean Duroc; KNP, Korean native pig; F1, DUC × KNP; F2, F1 × DUC; WRH, Woori-Heukdon (F1 × F2).

Supplementary Table 5. Location of ROH islands over 18 autosomal chromosomes.

Population	SSC1	SSC2	SSC3	SSC4	SSC5	SSC6	SSC7	SSC8	SSC9	SSC10	SSC11	SSC12	SSC13	SSC14	SSC15	SSC16	SSC17	SSC18
**DUC**	27-30; 232-237	86-89	50-53				26-30; 50-56							74-81; 91-102; 117-121				
**KNP**	52-57; 109-169; 263-270				63-68; 77-79		105-112	81-85	40-41; 46-52; 55-68	61-70	9-18; 70-72	38-41; 48-49	8-12; 15-18; 22-24; 27-45			3-7; 18-21; 25-26	10-11; 13-23; 25-27	
**F1**	54-56; 53-65; 83-87; 102-105; 173-175; 197-199; 220-223			78-80; 116-118	24-26; 102-105				47-51; 54-58; 57-58; 71-74; 127-129				32-34	30-33; 44-48	27-29	18-20; 27-30	37-39; 50-54	
**F2**	62-65; 83-87	86-87					26-30; 50-54		47-51; 54-58					75-78; 118-121	27-28	18-20	51-55	
**WRH**	220-224; 232-236		47-50; 52-55				50-54		47-51; 57-58					74-79; 91-99				

DUC, Korean Duroc; KNP, Korean native pig; F1, DUC × KNP; F2, F1 × DUC; WRH, Woori-Heukdon (F1 × F2).

Supplementary Table 6. ROH islands discovered among five pig populations, and annotated genes and QTLs to ROH islands.

SSC	SNP	SNP location	No. animals	ROH island begin	ROH island end	Population	Z score	p value	Genes	QTL
1	MARC0096577	27904779	736	27	28	DUC	3.275437	0.999473	BCLAF1,IFNGR1,IL20RA,IL22RA2,MAP3K5,MAP7,MTFR2,PDE7B,PEX7,SLC35D3	Exterior_QTL,Meat_and_Carcass_QTL,Meat_and_Carcass_eQTL,Production_Association,Exterior_Association,Health_Association,Reproduction_QTL,Meat_and_Carcass_Association,Health_QTL,Production_QTL,Reproduction_Association
1	ASGA0001911	28772718	752	28	29	DUC	3.375179	0.999631	AHI1,HBS1L,MYB,PDE7B	Exterior_QTL,Meat_and_Carcass_QTL,Meat_and_Carcass_eQTL,Production_Association,Exterior_Association,Health_Association,Reproduction_QTL,Meat_and_Carcass_Association,Health_QTL,Production_QTL,Reproduction_Association
1	ALGA0104675	29881873	769	29	30	DUC	3.480708	0.99975	ALDH8A1,HBS1L,SGK1,SLC2A12,TBPL1,TCF21	Exterior_QTL,Meat_and_Carcass_QTL,Meat_and_Carcass_eQTL,Production_Association,Exterior_Association,Health_Association,Reproduction_QTL,Meat_and_Carcass_Association,Health_QTL,Production_QTL,Reproduction_Association

1	ALGA0008440	232870019	849	232	233	DUC	3.978278	0.999965	TLE4	Meat_and_Carcass_QTL,Meat_and_Carcass_eQTL,Exterior_QTL,Health_Association,Meat_and_Carcass_Association,Health_QTL,Production_QTL,Reproduction_QTL
1	ALGA0008446	233490587	854	233	234	DUC	4.009403	0.99997	NPAP1	Meat_and_Carcass_QTL,Meat_and_Carcass_eQTL,Exterior_QTL,Health_QTL,Meat_and_Carcass_Association,Health_Association,Production_QTL,Reproduction_QTL
1	MARC0007004	234306334	854	234	235	DUC	4.009479	0.99997	LOC100524475,LOC102164346,SPATA31D1	Meat_and_Carcass_QTL,Meat_and_Carcass_eQTL,Exterior_QTL,Health_QTL,Meat_and_Carcass_Association,Health_Association,Production_QTL,Reproduction_QTL
1	MARC0006827	235008916	852	235	236	DUC	3.996524	0.999968	C1H9orf131,DNAJB5,FAM205C,FAM214B,FANCG,LOC100525024,LOC110255297,PHF24,PIGO,STOML2,UNC13B,VCP	Meat_and_Carcass_QTL,Meat_and_Carcass_eQTL,Reproduction_QTL,Meat_and_Carcass_Association,Health_QTL,Production_QTL,Exterior_QTL

1	ASGA0006128	236430981	734	236	237	DUC	3.26281	0.999448	ARHGEF39,CA9,CCDC107,CCIN,CD72,CLTA,CREB3,FAM166B,FAM221B,GBA2,GLIPR2,GNE,HINT2,HRCT1,LOC100152003,LOC100156421,LOC100157239,LOC100513390,LOC100514941,LOC100518591,LOC100519461,LOC110257045,LOC110257046,LOC110257048,MSMP,NPR2,RECK,RGP1,RUSC2,SIT1,SPAG8,TESK1,TLN1,TMEM8B,TPM2,UNC13B	Meat_and_Carcass_QTL,Meat_and_Carcass_eQTL,Reproduction_QTL,Meat_and_Carcass_Association,Health_QTL,Production_QTL,Exterior_QTL

2	H3GA0007030	86817792	799	86	87	DUC	3.66728	0.999877	AP3B1,OTP,PDE8B,TBCA,WDR41	Exterior_QTL,Meat_and_Carcass_eQTL,Meat_and_Carcass_QTL,Production_Association,Reproduction_QTL,Health_Association,Meat_and_Carcass_Association,Health_QTL,Production_QTL,Reproduction_Association,Exterior_Association
2	ASGA0010767	87610569	800	87	88	DUC	3.672956	0.99988	AP3B1,ARSB,BHMT,BHMT2,DMGDH,LHFPL2,SCAMP1	Meat_and_Carcass_eQTL,Meat_and_Carcass_QTL,Production_Association,Exterior_Association,Health_Association,Reproduction_QTL,Meat_and_Carcass_Association,Health_QTL,Production_QTL,Reproduction_Association,Exterior_QTL
2	ALGA0104357	88614116	794	88	89	DUC	3.636363	0.999862	CMYA5,HOMER1,JMY,MTX3,PAPD4,SERINC5,THBS4	Meat_and_Carcass_eQTL,Meat_and_Carcass_QTL,Production_Association,Exterior_Association,Health_Association,Reproduction_QTL,Meat_and_Carcass_Association,Health_QTL,Production_QTL,Reproduction_Association,Exterior_QTL
3	MARC0066148	50822015	707	50	51	DUC	3.093678	0.999012	-	Meat_and_Carcass_eQTL,Meat_and_Carcass_QTL,Production_Association,Exterior_QTL,Health_Association,Reproduction_QTL,Meat_and_Carcass_Association,Health_QTL,Production_QTL,Reproduction_Association,Exterior_Association

3	ALGA0018983	51768737	716	51	52	DUC	3.150221	0.999184	IL18R1,IL18RAP,IL1RL1,IL1RL2,MFSD9,SLC9A2,SLC9A4,TMEM182	Meat_and_Carcass_eQTL,Meat_and_Carcass_QTL,Production_Association,Exterior_QTL,Health_Association,Reproduction_QTL,Meat_and_Carcass_Association,Health_QTL,Production_QTL,Reproduction_Association,Exterior_Association
3	CASI0006548	52067601	716	52	53	DUC	3.150935	0.999186	CREG2,IL1R1,IL1R2,IL1RL2,MAP4K4,RFX8	Meat_and_Carcass_eQTL,Meat_and_Carcass_QTL,Production_Association,Exterior_QTL,Health_Association,Reproduction_QTL,Meat_and_Carcass_Association,Health_QTL,Production_QTL,Reproduction_Association,Exterior_Association
7	ALGA0122542	26976864	728	26	27	DUC	3.22429	0.999369	FAM83B,LRRC1,MLIP,TINAG	Exterior_QTL,Meat_and_Carcass_eQTL,Meat_and_Carcass_QTL,Production_Association,Exterior_Association,Health_Association,Reproduction_QTL,Meat_and_Carcass_Association,Health_QTL,Production_QTL,Reproduction_Association
7	MARC0043689	27826207	730	27	28	DUC	3.238349	0.999399	GCLC,KHDRBS2,KLHL31	Exterior_QTL,Meat_and_Carcass_QTL,Meat_and_Carcass_eQTL,Production_Association,Exterior_Association,Health_Association,Reproduction_QTL,Meat_and_Carcass_Association,Health_QTL,Production_QTL,Reproduction_Association

7	M1GA0009853	28212506	747	28	29	DUC	3.344132	0.999587	BAG2,BEND6,DST,PRIM2,RAB23,ZNF451	Exterior_QTL,Meat_and_Carcass_QTL,Meat_and_Carcass_eQTL,Production_Association,Exterior_Association,Health_Association,Reproduction_QTL,Meat_and_Carcass_Association,Health_QTL,Production_QTL,Reproduction_Association
7	ALGA0109063	29005809	731	29	30	DUC	3.243804	0.99941	B3GALT4,BAK1,C7H6orf125,COL21A1,CUTA,DAXX,DST,IP6K3,ITPR3,KIFC1,LEMD2,LOC100156231,PFDN6,PHF1,RGL2,RPS18,SYNGAP1,TAPBP,VPS52,WDR46,ZBTB22,ZBTB9	Exterior_QTL,Meat_and_Carcass_QTL,Meat_and_Carcass_eQTL,Production_Association,Exterior_Association,Health_Association,Reproduction_QTL,Meat_and_Carcass_Association,Health_QTL,Production_QTL,Reproduction_Association
7	H3GA0021764	50728561	882	50	51	DUC	4.183903	0.999986	EFL1,IL16,LOC102160759,MEX3B,SAXO2,STARD5,TMC3	Meat_and_Carcass_eQTL,Meat_and_Carcass_QTL,Production_Association,Exterior_QTL,Health_Association,Reproduction_QTL,Meat_and_Carcass_Association,Health_QTL,Production_QTL,Reproduction_Association,Exterior_Association

7	H3GA0021805	51999459	901	51	52	DUC	4.300169	0.999991	ADAMTSL3,BNC1,BTBD1,C7H15orf40,FAM103A1,HDGFL3,SH3GL3,TM6SF1	Meat_and_Carcass_eQTL,Meat_and_Carcass_QTL,Production_Association,Exterior_QTL,Health_Association,Reproduction_QTL,Meat_and_Carcass_Association,Health_QTL,Production_QTL,Reproduction_Association,Exterior_Association
7	MARC0074121	52994211	911	52	53	DUC	4.363014	0.999994	ALPK3,AP3B2,CPEB1,FSD2,HOMER2,NMB,PDE8A,RPS17,SEC11A,SLC28A1,WDR73,WHAMM,ZNF592,ZNF774,ZSCAN2	Meat_and_Carcass_eQTL,Meat_and_Carcass_QTL,Production_Association,Exterior_QTL,Health_Association,Reproduction_QTL,Meat_and_Carcass_Association,Health_QTL,Production_QTL,Reproduction_Association,Exterior_Association
7	M1GA0010426	53498685	911	53	54	DUC	4.364285	0.999994	ALDH1L1,BLM,CFAP100,CRTC3,FES,FURIN,HDDC3,IQGAP1,KLF15,MAN2A2,RCCD1,SLC41A3,UNC45A,UROC1,ZXDC	Meat_and_Carcass_eQTL,Meat_and_Carcass_QTL,Production_Association,Exterior_Association,Health_Association,Reproduction_QTL,Meat_and_Carcass_Association,Health_QTL,Production_QTL,Reproduction_Association,Exterior_QTL

7	H3GA0021869	54398347	834	54	55	DUC	3.885028	0.999949	ABHD2,ACAN,FANCI,HAPLN3,LOC100152264,LOC100153863,LOC100155865,LOC100156674,LOC100520032,LOC100736822,LOC100736871,LOC100737016,LOC100737631,LOC106504436,LOC106510141,LOC110261552,LOC110261553,MFGE8,POLG,RLBP1	Meat_and_Carcass_eQTL,Meat_and_Carcass_QTL,Production_Association,Exterior_Association,Health_Association,Reproduction_QTL,Meat_and_Carcass_Association,Health_QTL,Production_QTL,Reproduction_Association,Exterior_QTL
7	ASGA0034202	55128573	796	55	56	DUC	3.648915	0.999868	ANPEP,AP3S2,ARPIN,CIB1,GDPGP1,IDH2,KIF7,LOC100625251,LOC106510141,MESP1,MESP2,NGRN,PEX11A,PLIN1,PRC1,RHCG,SEMA4B,TICRR,VPS33B,WDR93,ZNF710	Meat_and_Carcass_eQTL,Meat_and_Carcass_QTL,Production_Association,Exterior_Association,Health_Association,Reproduction_QTL,Meat_and_Carcass_Association,Health_QTL,Production_QTL,Reproduction_Association,Exterior_QTL

14	H3GA0041018	74948147	847	74	75	DUC	3.965669	0.999963	ASCC1,C14H10orf105,CDH23,CHST3,LOC106506033,LOC110256823,PSAP,SLC29A3,SPOCK2,UNC5B,VSIR	Exterior_QTL,Meat_and_Carcass_eQTL,Meat_and_Carcass_QTL,Production_Association,Exterior_Association,Health_Association,Meat_and_Carcass_Association,Health_QTL,Production_QTL,Reproduction_Association,Reproduction_QTL
14	ASGA0064562	75712624	851	75	76	DUC	3.990504	0.999967	ANAPC16,ASCC1,DDIT4,DNAJB12,ECD,FAM149B1,MCU,MICU1,NUDT13,OIT3,P4HA1,PLA2G12B	Exterior_QTL,Meat_and_Carcass_eQTL,Meat_and_Carcass_QTL,Production_Association,Exterior_Association,Health_Association,Meat_and_Carcass_Association,Health_QTL,Production_QTL,Reproduction_Association,Reproduction_QTL
14	INRA0045033	76797619	851	76	77	DUC	3.990999	0.999967	ADK,ANXA7,AP3M1,CAMK2G,CFAP70,CHCHD1,DNAJC9,FAM149B1,FUT11,LOC102160869,LOC106506035,MRPS16,MSS51,MYOZ1,NDST2,PLAU,PPP3CB,SEC24C,SYNPO2L,USP54,VCL,ZSWIM8	Exterior_QTL,Meat_and_Carcass_eQTL,Meat_and_Carcass_QTL,Production_Association,Exterior_Association,Health_Association,Meat_and_Carcass_Association,Health_QTL,Production_QTL,Reproduction_Association,Reproduction_QTL

14	H3GA0041080	77991106	862	77	78	DUC	4.059031	0.999975	ADK,COMTD1,DUPD1,DUSP13,KAT6B,LOC106507128,SAMD8,VDAC2,ZNF503	Exterior_QTL,Meat_and_Carcass_eQTL,Meat_and_Carcass_QTL,Production_Association,Exterior_Association,Health_Association,Meat_and_Carcass_Association,Health_QTL,Production_QTL,Reproduction_Association,Reproduction_QTL
14	INRA0045123	78969498	864	78	79	DUC	4.07159	0.999977	-	Exterior_QTL,Meat_and_Carcass_eQTL,Meat_and_Carcass_QTL,Production_Association,Exterior_Association,Health_Association,Meat_and_Carcass_Association,Health_QTL,Production_QTL,Reproduction_Association,Reproduction_QTL
14	BGIS0006306	79383783	864	79	80	DUC	4.071671	0.999977	KCNMA1	Exterior_QTL,Meat_and_Carcass_eQTL,Meat_and_Carcass_QTL,Production_Association,Exterior_Association,Health_Association,Meat_and_Carcass_Association,Health_QTL,Production_QTL,Reproduction_Association,Reproduction_QTL
14	ASGA0064701	80059618	847	80	81	DUC	3.965229	0.999963	DLG5,KCNMA1,POLR3A,RPS24	Exterior_QTL,Meat_and_Carcass_eQTL,Meat_and_Carcass_QTL,Production_Association,Exterior_Association,Health_Association,Meat_and_Carcass_Association,Health_QTL,Production_QTL,Reproduction_Association,Reproduction_QTL

14	INRA0045780	91911925	840	91	92	DUC	3.922467	0.999956	C14H10orf10,CXCL12,LOC100157415,RASSF4,TMEM72,ZNF22	Exterior_QTL,Meat_and_Carcass_eQTL,Meat_and_Carcass_QTL,Production_Association,Reproduction_QTL,Health_Association,Meat_and_Carcass_Association,Health_QTL,Production_QTL,Reproduction_Association,Exterior_Association
14	ALGA0080009	92935518	848	92	93	DUC	3.971997	0.999964	CISD1,IPMK,LOC100524282,TFAM,UBE2D1,ZNF239,ZNF32	Exterior_QTL,Meat_and_Carcass_eQTL,Meat_and_Carcass_QTL,Production_Association,Exterior_Association,Health_Association,Reproduction_QTL,Meat_and_Carcass_Association,Health_QTL,Production_QTL,Reproduction_Association
14	ALGA0080040	93892965	848	93	94	DUC	3.972428	0.999964	-	Exterior_QTL,Meat_and_Carcass_eQTL,Meat_and_Carcass_QTL,Production_Association,Reproduction_QTL,Health_Association,Meat_and_Carcass_Association,Health_QTL,Production_QTL,Reproduction_Association,Exterior_Association
14	ASGA0065434	94846149	848	94	95	DUC	3.97242	0.999964	LOC106506082,ZWINT	Exterior_QTL,Meat_and_Carcass_eQTL,Meat_and_Carcass_QTL,Production_Association,Reproduction_QTL,Health_Association,Meat_and_Carcass_Association,Health_QTL,Production_QTL,Reproduction_Association,Exterior_Association

14	ALGA0080090	95280664	848	95	96	DUC	3.971859	0.999964	PCDH15	Exterior_QTL,Meat_and_Carcass_eQTL,Meat_and_Carcass_QTL,Production_Association,Reproduction_QTL,Health_Association,Meat_and_Carcass_Association,Health_QTL,Production_QTL,Reproduction_Association,Exterior_Association
14	MARC0041529	96845762	849	96	97	DUC	3.97808	0.999965	LOC102157770,LOC110256792,PCDH15	Exterior_QTL,Meat_and_Carcass_eQTL,Meat_and_Carcass_QTL,Production_Association,Reproduction_QTL,Health_Association,Meat_and_Carcass_Association,Health_QTL,Production_QTL,Reproduction_Association,Exterior_Association
14	ALGA0080189	97412967	849	97	98	DUC	3.978542	0.999965	DKK1,MBL2,PRKG1	Exterior_QTL,Meat_and_Carcass_eQTL,Meat_and_Carcass_QTL,Production_Association,Reproduction_QTL,Health_Association,Meat_and_Carcass_Association,Health_QTL,Production_QTL,Reproduction_Association,Exterior_Association
14	H3GA0041643	98536822	850	98	99	DUC	3.984857	0.999966	A1CF,CSTF2T,PRKG1	Exterior_QTL,Meat_and_Carcass_eQTL,Meat_and_Carcass_QTL,Production_Association,Reproduction_QTL,Health_Association,Meat_and_Carcass_Association,Health_QTL,Production_QTL,Reproduction_Association,Exterior_Association

14	ALGA0080315	99014725	843	99	100	DUC	3.940667	0.999959	ASAH2,ATAD1,KLLN,MINPP1,PAPSS2,PTEN,SGMS1	Exterior_QTL,Meat_and_Carcass_eQTL,Meat_and_Carcass_QTL,Production_Association,Reproduction_QTL,Health_Association,Meat_and_Carcass_Association,Health_QTL,Production_QTL,Reproduction_Association,Exterior_Association
14	ALGA0080371	100011521	840	100	101	DUC	3.921128	0.999956	ACTA2,ANKRD22,FAS,LIPM,LOC100156277,LOC100157486,PTEN,RNLS,STAMBPL1	Exterior_QTL,Meat_and_Carcass_eQTL,Meat_and_Carcass_QTL,Production_Association,Reproduction_QTL,Health_Association,Meat_and_Carcass_Association,Health_QTL,Production_QTL,Reproduction_Association,Exterior_Association
14	ASGA0065631	101133523	825	101	102	DUC	3.828352	0.999935	CH25H,IFIT1,IFIT2,IFIT3,KIF20B,LIPA,LOC100151816,PANK1,SLC16A12	Exterior_QTL,Meat_and_Carcass_eQTL,Meat_and_Carcass_QTL,Production_Association,Exterior_Association,Health_Association,Meat_and_Carcass_Association,Health_QTL,Production_QTL,Reproduction_Association,Reproduction_QTL
14	MARC0026567	117825278	879	117	118	DUC	4.164984	0.999984	SORCS1	Exterior_QTL,Meat_and_Carcass_eQTL,Meat_and_Carcass_QTL,Production_Association,Exterior_Association,Health_Association,Meat_and_Carcass_Association,Health_QTL,Production_QTL,Reproduction_Association,Reproduction_QTL

14	H3GA0042201	118110675	904	118	119	DUC	4.319529	0.999992	LOC102162193,SORCS1	Exterior_QTL,Meat_and_Carcass_eQTL,Meat_and_Carcass_QTL,Production_Association,Reproduction_QTL,Health_Association,Meat_and_Carcass_Association,Health_QTL,Production_QTL,Reproduction_Association,Exterior_Association
14	MARC0029634	119371055	894	119	120	DUC	4.258202	0.99999	-	Exterior_QTL,Meat_and_Carcass_eQTL,Meat_and_Carcass_QTL,Production_Association,Exterior_Association,Health_Association,Meat_and_Carcass_Association,Health_QTL,Production_QTL,Reproduction_Association,Reproduction_QTL
14	INRA0047112	120375842	897	120	121	DUC	4.274977	0.99999	ADD3,DUSP5,MXI1,SMC3,SMNDC1,XPNPEP1	Meat_and_Carcass_eQTL,Meat_and_Carcass_QTL,Production_Association,Exterior_Association,Health_Association,Reproduction_QTL,Meat_and_Carcass_Association,Health_QTL,Production_QTL,Reproduction_Association,Exterior_QTL
1	ASGA0087818	54910237	8	54	55	F1	4.597807	0.999998	NT5E,SNX14,SYNCRIP	Meat_and_Carcass_eQTL,Meat_and_Carcass_QTL,Production_Association,Exterior_Association,Health_Association,Reproduction_QTL,Meat_and_Carcass_Association,Health_QTL,Production_QTL,Reproduction_Association,Exterior_QTL

1	ASGA0002948	55226608	8	55	56	F1	4.681942	0.999999	C1H6orf163,CFAP206,CGA,GJB7,HTR1E,LOC100151959,ORC3,RARS2,SLC35A1,SMIM8,ZNF292	Meat_and_Carcass_eQTL,Meat_and_Carcass_QTL,Production_Association,Exterior_Association,Health_Association,Reproduction_QTL,Meat_and_Carcass_Association,Health_QTL,Production_QTL,Reproduction_Association,Exterior_QTL
1	ASGA0097034	63785647	7	63	64	F1	4.070645	0.999977	FHL5,GPR63,UFL1	Exterior_QTL,Meat_and_Carcass_eQTL,Meat_and_Carcass_QTL,Production_Association,Reproduction_QTL,Health_Association,Meat_and_Carcass_Association,Health_QTL,Production_QTL,Reproduction_Association,Exterior_Association
1	INRA0002704	64278197	7	64	65	F1	4.071137	0.999977	KLHL32,MMS22L,NDUFAF4	Exterior_QTL,Meat_and_Carcass_eQTL,Meat_and_Carcass_QTL,Production_Association,Exterior_Association,Health_Association,Meat_and_Carcass_Association,Health_QTL,Production_QTL,Reproduction_Association,Reproduction_QTL
1	ALGA0004703	83829017	6	83	84	F1	3.34703	0.999592	DOPEY1,IBTK,PGM3,RWDD2A,TPBG,UBE3D	Exterior_QTL,Meat_and_Carcass_eQTL,Meat_and_Carcass_QTL,Production_Association,Exterior_Association,Health_Association,Meat_and_Carcass_Association,Health_QTL,Production_QTL,Reproduction_Association,Reproduction_QTL

1	ALGA0004729	84854279	11	84	85	F1	6.589073	1	FAM46A,IBTK,RPL18	Exterior_QTL,Meat_and_Carcass_eQTL,Meat_and_Carcass_QTL,Production_Association,Exterior_Association,Health_Association,Meat_and_Carcass_Association,Health_QTL,Production_QTL,Reproduction_Association,Reproduction_QTL
1	INRA0003188	85631812	11	85	86	F1	6.605837	1	BCKDHB	Exterior_QTL,Meat_and_Carcass_eQTL,Meat_and_Carcass_QTL,Production_Association,Exterior_Association,Health_Association,Meat_and_Carcass_Association,Health_QTL,Production_QTL,Reproduction_Association,Reproduction_QTL
1	ALGA0113211	86042306	9	86	87	F1	5.200135	1	BCKDHB,ELOVL4,HMGN3,LCA5,SH3BGRL2,TTK	Exterior_QTL,Meat_and_Carcass_eQTL,Meat_and_Carcass_QTL,Production_Association,Reproduction_QTL,Health_Association,Meat_and_Carcass_Association,Health_QTL,Production_QTL,Reproduction_Association,Exterior_Association
1	CASI0004348	102841851	6	102	103	F1	3.437544	0.999706	DCC	Exterior_QTL,Meat_and_Carcass_eQTL,Meat_and_Carcass_QTL,Production_Association,Reproduction_QTL,Health_Association,Meat_and_Carcass_Association,Health_QTL,Production_QTL,Reproduction_Association,Exterior_Association

1	DRGA0001422	103186451	6	103	104	F1	3.44642	0.999716	C1H18orf54,DCC,LOC102160380,MBD2,POLI,STARD6	Exterior_QTL,Meat_and_Carcass_eQTL,Meat_and_Carcass_QTL,Production_Association,Reproduction_QTL,Health_Association,Meat_and_Carcass_Association,Health_QTL,Production_QTL,Reproduction_Association,Exterior_Association
1	ALGA0005325	104496423	6	104	105	F1	3.440907	0.99971	CCDC68,LOC106508962,LOC110259490,RAB27B,TCF4	Exterior_QTL,Meat_and_Carcass_eQTL,Meat_and_Carcass_QTL,Production_Association,Reproduction_QTL,Health_Association,Meat_and_Carcass_Association,Health_QTL,Production_QTL,Reproduction_Association,Exterior_Association
1	DRGA0001676	173146830	6	173	174	F1	3.448868	0.999719	-	Meat_and_Carcass_QTL,Meat_and_Carcass_eQTL,Production_Association,Exterior_QTL,Health_QTL,Reproduction_QTL,Meat_and_Carcass_Association,Health_Association,Production_QTL,Reproduction_Association
1	ASGA0005318	174192907	6	174	175	F1	3.404206	0.999668	FSCB	Meat_and_Carcass_QTL,Meat_and_Carcass_eQTL,Production_Association,Exterior_QTL,Exterior_Association,Health_Association,Reproduction_QTL,Health_QTL,Production_QTL,Reproduction_Association

1	H3GA0003526	197229674	11	197	198	F1	6.603161	1	LOC110259585,TUSC1	Meat_and_Carcass_QTL,Meat_and_Carcass_eQTL,Exterior_QTL,Exterior_Association,Health_Association,Health_QTL,Production_QTL,Reproduction_QTL
1	ALGA0007544	198154616	11	198	199	F1	6.594488	1	ELAVL2,IZUMO3	Meat_and_Carcass_QTL,Meat_and_Carcass_eQTL,Exterior_QTL,Health_Association,Meat_and_Carcass_Association,Health_QTL,Production_QTL,Reproduction_Association,Reproduction_QTL
1	INRA0006084	220967018	7	220	221	F1	3.997069	0.999968	DMRT1,DMRT2,DMRT3	Meat_and_Carcass_QTL,Meat_and_Carcass_eQTL,Exterior_QTL,Exterior_Association,Health_Association,Health_QTL,Production_QTL,Reproduction_QTL
1	DRGA0001990	221439097	7	221	222	F1	4.06771	0.999976	C1H9orf66,DOCK8,KANK1,LOC100155087,LOC100156293,PGM5,TMEM252	Meat_and_Carcass_QTL,Meat_and_Carcass_eQTL,Exterior_QTL,Health_Association,Meat_and_Carcass_Association,Health_QTL,Production_QTL,Reproduction_QTL
1	DRGA0002004	222663189	7	222	223	F1	4.075809	0.999977	APBA1,FAM122A,FAM189A2,FXN,PIP5K1B,TJP2	Meat_and_Carcass_QTL,Meat_and_Carcass_eQTL,Exterior_QTL,Health_Association,Meat_and_Carcass_Association,Health_QTL,Production_QTL,Reproduction_Association,Reproduction_QTL

4	MARC0055526	78889604	6	78	79	F1	3.444541	0.999714	PCMTD1,SNTG1,ST18	Exterior_QTL,Meat_and_Carcass_eQTL,Meat_and_Carcass_QTL,Production_Association,Exterior_Association,Health_Association,Meat_and_Carcass_Association,Health_QTL,Production_QTL,Reproduction_Association,Reproduction_QTL
4	ALGA0026197	79142579	6	79	80	F1	3.448327	0.999718	CEBPD,EFCAB1,MCM4,PRKDC,SNAI2,SPIDR,UBE2V2	Exterior_QTL,Meat_and_Carcass_eQTL,Meat_and_Carcass_QTL,Production_Association,Exterior_Association,Health_Association,Meat_and_Carcass_Association,Health_QTL,Production_QTL,Reproduction_Association,Reproduction_QTL
4	M1GA0006491	116963398	6	116	117	F1	3.412475	0.999678	OLFM3	Exterior_QTL,Meat_and_Carcass_eQTL,Meat_and_Carcass_QTL,Production_Association,Exterior_Association,Health_Association,Meat_and_Carcass_Association,Health_QTL,Production_QTL,Reproduction_Association,Reproduction_QTL
4	MARC0072124	117108324	6	117	118	F1	3.445499	0.999715	CDC14A,DBT,DPH5,EXTL2,GPR88,LRRC39,RTCA,S1PR1,SASS6,SLC30A7,TRMT13,VCAM1	Exterior_QTL,Meat_and_Carcass_eQTL,Meat_and_Carcass_QTL,Production_Association,Exterior_Association,Health_Association,Meat_and_Carcass_Association,Health_QTL,Production_QTL,Reproduction_Association,Reproduction_QTL

5	ALGA0031362	24871192	10	24	25	F1	5.966219	1	LOC102166013,LRIG3,SLC16A7	Exterior_QTL,Meat_and_Carcass_QTL,Meat_and_Carcass_eQTL,Production_Association,Exterior_Association,Health_Association,Reproduction_QTL,Meat_and_Carcass_Association,Health_QTL,Production_QTL,Reproduction_Association
5	ASGA0025173	25905765	10	25	26	F1	5.97325	1	-	Exterior_QTL,Meat_and_Carcass_QTL,Meat_and_Carcass_eQTL,Production_Association,Exterior_Association,Health_Association,Meat_and_Carcass_Association,Health_QTL,Production_QTL,Reproduction_Association,Reproduction_QTL
5	ALGA0034265	102962961	7	102	103	F1	3.967043	0.999964	SYT1	Exterior_QTL,Meat_and_Carcass_eQTL,Meat_and_Carcass_QTL,Production_Association,Reproduction_QTL,Health_Association,Meat_and_Carcass_Association,Health_QTL,Production_QTL,Reproduction_Association,Exterior_Association
5	ASGA0027314	103693706	7	103	104	F1	4.071626	0.999977	NAV3	Exterior_QTL,Meat_and_Carcass_eQTL,Meat_and_Carcass_QTL,Production_Association,Reproduction_QTL,Health_Association,Meat_and_Carcass_Association,Health_QTL,Production_QTL,Reproduction_Association,Exterior_Association

5	ALGA0034219	104117970	7	104	105	F1	4.070338	0.999977	CSRP2,E2F7,LOC110260809,ZDHHC17	Exterior_QTL,Meat_and_Carcass_eQTL,Meat_and_Carcass_QTL,Production_Association,Reproduction_QTL,Health_Association,Meat_and_Carcass_Association,Health_QTL,Production_QTL,Reproduction_Association,Exterior_Association
9	INRA0031791	47798392	11	47	48	F1	6.578337	1	ARHGEF12,GRIK4,OAF,POU2F3,TMEM136,TRIM29	Meat_and_Carcass_eQTL,Meat_and_Carcass_QTL,Production_Association,Exterior_QTL,Health_Association,Reproduction_QTL,Meat_and_Carcass_Association,Health_QTL,Production_QTL,Reproduction_Association,Exterior_Association
9	H3GA0027334	48396618	11	48	49	F1	6.597328	1	GRIK4,LOC100523684,SC5D,SORL1	Meat_and_Carcass_eQTL,Meat_and_Carcass_QTL,Production_Association,Exterior_QTL,Health_Association,Reproduction_QTL,Meat_and_Carcass_Association,Health_QTL,Production_QTL,Reproduction_Association,Exterior_Association
9	ASGA0043094	49681169	11	49	50	F1	6.59575	1	BSX,C9H11orf63,CLMP,CRTAM,HSPA8,LOC102164776,UBASH3B	Meat_and_Carcass_eQTL,Meat_and_Carcass_QTL,Production_Association,Exterior_QTL,Health_Association,Reproduction_QTL,Meat_and_Carcass_Association,Health_QTL,Production_QTL,Reproduction_Association,Exterior_Association

9	ASGA0093525	50162529	9	50	51	F1	5.249735	1	CLMP,GRAMD1B,LOC100512247,LOC100513369,LOC100513559,LOC100515746,LOC100518267,LOC100518623,LOC100518799,LOC100518982,LOC100524388,LOC100524576,LOC100524757,LOC100524947,LOC100525119,LOC100525298,LOC100525476,LOC106504899,LOC106504900,LOC106504901,LOC106504905,LOC106504908,LOC106504911,LOC106504912,LOC110262281,LOC110262293,SCN3B,TMEM225,ZNF202	Meat_and_Carcass_eQTL,Meat_and_Carcass_QTL,Production_Association,Exterior_QTL,Health_Association,Reproduction_QTL,Meat_and_Carcass_Association,Health_QTL,Production_QTL,Reproduction_Association,Exterior_Association

9	H3GA0027478	54928486	10	54	55	F1	5.951482	1	KIRREL3	Meat_and_Carcass_eQTL,Meat_and_Carcass_QTL,Production_Association,Exterior_Association,Health_Association,Reproduction_QTL,Meat_and_Carcass_Association,Health_QTL,Production_QTL,Reproduction_Association,Exterior_QTL
9	ALGA0053182	55387693	10	55	56	F1	5.975909	1	ARHGAP32,ETS1,FLI1,KCNJ1,KCNJ5,LOC100516820	Meat_and_Carcass_eQTL,Meat_and_Carcass_QTL,Production_Association,Exterior_Association,Health_Association,Reproduction_QTL,Meat_and_Carcass_Association,Health_QTL,Production_QTL,Reproduction_Association,Exterior_QTL
9	ASGA0090595	56541164	10	56	57	F1	5.953199	1	APLP2,ARHGAP32,BARX2,LOC100516455,NFRKB,PRDM10,ST14,TMEM45B,ZBTB44	Meat_and_Carcass_eQTL,Meat_and_Carcass_QTL,Production_Association,Exterior_Association,Health_Association,Reproduction_QTL,Meat_and_Carcass_Association,Health_QTL,Production_QTL,Reproduction_Association,Exterior_QTL
9	ASGA0085584	57093003	10	57	58	F1	5.820192	1	ADAMTS15,ADAMTS8,LOC102165987,NTM,SNX19,ZBTB44	Meat_and_Carcass_eQTL,Meat_and_Carcass_QTL,Production_Association,Exterior_Association,Health_Association,Reproduction_QTL,Meat_and_Carcass_Association,Health_QTL,Production_QTL,Reproduction_Association,Exterior_QTL

9	DRGA0009459	71970976	6	71	72	F1	3.447945	0.999718	AKAP9,CDK14,FZD1,MTERF1	Exterior_QTL,Meat_and_Carcass_eQTL,Meat_and_Carcass_QTL,Production_Association,Exterior_Association,Health_Association,Meat_and_Carcass_Association,Health_QTL,Production_QTL,Reproduction_Association,Reproduction_QTL
9	MARC0111137	72808605	6	72	73	F1	3.445904	0.999715	AKAP9,ANKIB1,CYP51,FAM133B,GATAD1,KRIT1,LOC100519098,LOC100519264,LOC110255562,LRRD1,PEX1,RBM48	Exterior_QTL,Meat_and_Carcass_eQTL,Meat_and_Carcass_QTL,Production_Association,Exterior_Association,Health_Association,Meat_and_Carcass_Association,Health_QTL,Production_QTL,Reproduction_Association,Reproduction_QTL
9	ISU10000749	73292979	6	73	74	F1	3.351499	0.999598	BET1,CALCR,GNG11,GNGT1,HEPACAM2,TFPI2,VPS50	Exterior_QTL,Meat_and_Carcass_eQTL,Meat_and_Carcass_QTL,Production_Association,Reproduction_QTL,Health_Association,Meat_and_Carcass_Association,Health_QTL,Production_QTL,Reproduction_Association,Exterior_Association
9	DRGA0009883	127647102	9	127	128	F1	5.342364	1	C9H1orf27,HMCN1,PDC,PLA2G4A,PRG4,PTGS2,TPR	Meat_and_Carcass_QTL,Meat_and_Carcass_eQTL,Production_Association,Reproduction_QTL,Exterior_Association,Health_Association,Meat_and_Carcass_Association,Health_QTL,Production_QTL,Reproduction_Association,Exterior_QTL

9	ASGA0044843	128220123	9	128	129	F1	5.324813	1	CENPF,KCNK2,PLA2G4A	Meat_and_Carcass_QTL,Meat_and_Carcass_eQTL,Production_Association,Exterior_QTL,Exterior_Association,Health_Association,Meat_and_Carcass_Association,Health_QTL,Production_QTL,Reproduction_Association,Reproduction_QTL
13	ALGA0069467	32901267	10	32	33	F1	5.961592	1	AMT,APEH,BSN,CACNA2D2,CAMKV,CDHR4,DAG1,FAM212A,GMPPB,GNAI2,GNAT1,HYAL1,HYAL2,HYAL3,IFRD2,IP6K1,LOC100517372,LSMEM2,MON1A,MST1,MST1R,NAT6,NICN1,NPRL2,RASSF1,RBM5,RBM6,RNF123,SEMA3B,SEMA3F,SLC38A3,TMEM115,TRAIP,TUSC2,UBA7,ZMYND10	Exterior_QTL,Meat_and_Carcass_QTL,Meat_and_Carcass_eQTL,Production_Association,Exterior_Association,Health_Association,Reproduction_QTL,Meat_and_Carcass_Association,Health_QTL,Production_QTL,Reproduction_Association

13	H3GA0036200	33248240	10	33	34	F1	5.976576	1	C13H3orf18,CISH,DCAF1,DOCK3,GRM2,HEMK1,MANF,MAPKAPK3,RAD54L2,RBM15B,TEX264	Exterior_QTL,Meat_and_Carcass_QTL,Meat_and_Carcass_eQTL,Production_Association,Exterior_Association,Health_Association,Reproduction_QTL,Meat_and_Carcass_Association,Health_QTL,Production_QTL,Reproduction_Association
14	H3GA0039661	30997622	6	30	31	F1	3.441969	0.999711	B3GNT4,BCL7A,CCDC62,CLIP1,DENR,DIABLO,HCAR1,HCAR2,HIP1R,HPD,IL31,KNTC1,LRRC43,MLXIP,MORN3,PSMD9,RHOF,RSRC2,SETD1B,TMEM120B,VPS33A,WDR66,ZCCHC8	Exterior_QTL,Meat_and_Carcass_eQTL,Meat_and_Carcass_QTL,Production_Association,Exterior_Association,Health_Association,Reproduction_QTL,Meat_and_Carcass_Association,Health_QTL,Production_QTL,Reproduction_Association
14	DIAS0001236	31911126	6	31	32	F1	3.438886	0.999708	ANAPC5,ANAPC7,ARPC3,ATP2A2,CAMKK2,FAM216A,GPN3,IFT81,KDM2B,MORN3,ORAI1,P2RX4,P2RX7,PPTC7,RAD9B,RNF34,TCTN1,VPS29	Exterior_QTL,Meat_and_Carcass_eQTL,Meat_and_Carcass_QTL,Production_Association,Reproduction_QTL,Health_Association,Meat_and_Carcass_Association,Health_QTL,Production_QTL,Reproduction_Association,Exterior_Association

14	ASGA0062561	32037651	6	32	33	F1	3.355996	0.999605	ACAD10,ATXN2,BRAP,CCDC63,CUX2,FAM109A,HVCN1,MYL2,PPP1CC,SH2B3,TCTN1	Exterior_QTL,Meat_and_Carcass_QTL,Meat_and_Carcass_eQTL,Production_Association,Exterior_Association,Health_Association,Reproduction_QTL,Meat_and_Carcass_Association,Health_QTL,Production_QTL,Reproduction_Association
14	ASGA0063175	44986228	6	44	45	F1	3.338421	0.999579	ASPHD2,CRYBA4,CRYBB1,HPS4,SEZ6L,SRRD,TFIP11,TPST2	Meat_and_Carcass_eQTL,Meat_and_Carcass_QTL,Production_Association,Exterior_QTL,Health_Association,Reproduction_QTL,Meat_and_Carcass_Association,Health_QTL,Production_QTL,Reproduction_Association,Exterior_Association
14	ALGA0077352	45167309	6	45	46	F1	3.424428	0.999692	CCDC117,CHEK2,HSCB,LOC100155267,MN1,PITPNB,TTC28	Meat_and_Carcass_eQTL,Meat_and_Carcass_QTL,Production_Association,Exterior_QTL,Health_Association,Reproduction_QTL,Meat_and_Carcass_Association,Health_QTL,Production_QTL,Reproduction_Association,Exterior_Association

14	ASGA0063232	46912685	6	46	47	F1	3.412974	0.999679	AP1B1,ASCC2,C14H22orf31,CABP7,CCDC117,EMID1,EWSR1,GAS2L1,KREMEN1,MTMR3,NEFH,NF2,NIPSNAP1,RASL10A,RHBDD3,UQCR10,XBP1,ZMAT5,ZNRF3	Meat_and_Carcass_eQTL,Meat_and_Carcass_QTL,Production_Association,Exterior_QTL,Health_Association,Reproduction_QTL,Meat_and_Carcass_Association,Health_QTL,Production_QTL,Reproduction_Association,Exterior_Association
14	MARC0024032	47712987	6	47	48	F1	3.444482	0.999714	C14H5orf52,CASTOR1,CCDC157,DUSP18,GAL3ST1,INPP5J,LIF,LIMK2,LOC100156470,MORC2,MTFP1,MTMR3,OSBP2,OSM,PES1,PLA2G3,RNF185,RNF215,SEC14L2,SELENOM,SF3A1,SLC35E4,SMTN,TBC1D10A,TCN2	Meat_and_Carcass_eQTL,Meat_and_Carcass_QTL,Production_Association,Exterior_QTL,Health_Association,Reproduction_QTL,Meat_and_Carcass_Association,Health_QTL,Production_QTL,Reproduction_Association,Exterior_Association

15	ASGA0069085	27223506	8	27	28	F1	4.704688	0.999999	CNTNAP5	Exterior_QTL,Meat_and_Carcass_QTL,Meat_and_Carcass_eQTL,Production_Association,Exterior_Association,Health_Association,Reproduction_QTL,Meat_and_Carcass_Association,Health_QTL,Production_QTL,Reproduction_Association
15	INRA0049035	28400201	8	28	29	F1	4.700887	0.999999	-	Exterior_QTL,Meat_and_Carcass_QTL,Meat_and_Carcass_eQTL,Production_Association,Exterior_Association,Health_Association,Reproduction_QTL,Meat_and_Carcass_Association,Health_QTL,Production_QTL,Reproduction_Association
16	MARC0054728	18818606	10	18	19	F1	5.923512	1	LOC110257335,MTMR12,NPR3,PDZD2,SUB1,ZFR	Exterior_QTL,Meat_and_Carcass_QTL,Meat_and_Carcass_eQTL,Production_Association,Exterior_Association,Health_Association,Meat_and_Carcass_Association,Health_QTL,Production_QTL,Reproduction_Association,Reproduction_QTL
16	ASGA0072472	19319756	10	19	20	F1	5.951212	1	ADAMTS12,RXFP3,SLC45A2,TARS	Exterior_QTL,Meat_and_Carcass_eQTL,Meat_and_Carcass_QTL,Production_Association,Exterior_Association,Health_Association,Reproduction_QTL,Meat_and_Carcass_Association,Health_QTL,Production_QTL,Reproduction_Association

16	ALGA0120801	27608306	6	27	28	F1	3.395971	0.999658	ANXA2R,CCDC152,CCL28,GHR,HMGCS1,LOC102162127,LOC102162264,LOC110257336,LOC110257337, LOC110257338,NIM1K, SELENOP,ZNF131	Exterior_QTL,Meat_and_Carcass_QTL,Meat_and_Carcass_eQTL,Production_Association,Exterior_Association,Health_Association,Reproduction_QTL,Meat_and_Carcass_Association,Health_QTL,Production_QTL,Reproduction_Association
16	H3GA0046366	28441774	6	28	29	F1	3.443949	0.999713	C16H5orf34,CCL28,FGF10,NNT,PAIP1,TMEM267	Exterior_QTL,Meat_and_Carcass_QTL,Meat_and_Carcass_eQTL,Production_Association,Exterior_Association,Health_Association,Reproduction_QTL,Meat_and_Carcass_Association,Health_QTL,Production_QTL,Reproduction_Association
16	H3GA0046372	29076271	6	29	30	F1	3.450354	0.99972	HCN1,MRPS30	Exterior_QTL,Meat_and_Carcass_QTL,Meat_and_Carcass_eQTL,Production_Association,Exterior_Association,Health_Association,Reproduction_QTL,Meat_and_Carcass_Association,Health_QTL,Production_QTL,Reproduction_Association

17	ALGA0094877	37341393	10	37	38	F1	5.969296	1	ACTL10,AHCY,ASIP,CBFA2T2,CHMP4B,DYNLRB1,E2F1,EIF2S2,MAP1LC3A,NECAB3,PIGU,PXMP4,RALY,ZNF341	Meat_and_Carcass_eQTL,Meat_and_Carcass_QTL,Production_Association,Exterior_QTL,Health_Association,Reproduction_QTL,Meat_and_Carcass_Association,Health_QTL,Production_QTL,Reproduction_Association,Exterior_Association
17	ASGA0076852	38331769	10	38	39	F1	5.973593	1	ACSS2,C17H20orf173,CEP250,CPNE1,EDEM2,EIF6,ERGIC3,FAM83C,FER1L4,GDF5,GGT7,GSS,MMP24,MYH7B,NCOA6,PIGU,PROCR,RBM12,SPAG4,TP53INP2,TRPC4AP,UQCC	Meat_and_Carcass_eQTL,Meat_and_Carcass_QTL,Production_Association,Exterior_Association,Health_Association,Reproduction_QTL,Meat_and_Carcass_Association,Health_QTL,Production_QTL,Reproduction_Association,Exterior_QTL
17	H3GA0049350	50498118	8	50	51	F1	4.695511	0.999999	ARFGEF2,CSE1L,DDX27,PREX1,STAU1,ZNFX1	Meat_and_Carcass_eQTL,Meat_and_Carcass_QTL,Production_Association,Exterior_QTL,Health_Association,Reproduction_QTL,Meat_and_Carcass_Association,Health_QTL,Production_QTL,Reproduction_Association,Exterior_Association

17	ALGA0095686	51218943	8	51	52	F1	4.671005	0.999999	B4GALT5,CEBPB,IL29,KCNB1,LOC100524118,PTGIS,RNF114,SLC9A8,SNAI1,SPATA2,TMEM189,UBE2V1	Meat_and_Carcass_eQTL,Meat_and_Carcass_QTL,Production_Association,Exterior_QTL,Health_Association,Reproduction_QTL,Meat_and_Carcass_Association,Health_QTL,Production_QTL,Reproduction_Association,Exterior_Association
17	H3GA0049430	52143266	8	52	53	F1	4.680665	0.999999	ADNP,ATP9A,BCAS4,DPM1,KCNG1,MOCS3,NFATC2,PARD6B,PTPN1,RIPOR3	Meat_and_Carcass_eQTL,Meat_and_Carcass_QTL,Production_Association,Exterior_QTL,Health_Association,Reproduction_QTL,Meat_and_Carcass_Association,Health_QTL,Production_QTL,Reproduction_Association,Exterior_Association
17	ASGA0077672	53168652	8	53	54	F1	4.683921	0.999999	ATP9A,SALL4,ZFP64	Meat_and_Carcass_eQTL,Meat_and_Carcass_QTL,Production_Association,Exterior_Association,Health_Association,Reproduction_QTL,Meat_and_Carcass_Association,Health_QTL,Production_QTL,Reproduction_Association,Exterior_QTL
1	ASGA0003194	62811729	70	62	63	F2	3.289779	0.999499	MANEA	Exterior_QTL,Meat_and_Carcass_eQTL,Meat_and_Carcass_QTL,Production_Association,Exterior_Association,Health_Association,Meat_and_Carcass_Association,Health_QTL,Production_QTL,Reproduction_Association,Reproduction_QTL

1	DRGA0001092	63726056	78	63	64	F2	3.793217	0.999926	FHL5,GPR63,UFL1	Exterior_QTL,Meat_and_Carcass_eQTL,Meat_and_Carcass_QTL,Production_Association,Reproduction_QTL,Health_Association,Meat_and_Carcass_Association,Health_QTL,Production_QTL,Reproduction_Association,Exterior_Association
1	ASGA0003216	64079226	78	64	65	F2	3.793105	0.999926	KLHL32,MMS22L,NDUFAF4	Exterior_QTL,Meat_and_Carcass_eQTL,Meat_and_Carcass_QTL,Production_Association,Exterior_Association,Health_Association,Meat_and_Carcass_Association,Health_QTL,Production_QTL,Reproduction_Association,Reproduction_QTL
1	ALGA0004705	83951904	69	83	84	F2	3.215966	0.99935	DOPEY1,IBTK,PGM3,RWDD2A,TPBG,UBE3D	Exterior_QTL,Meat_and_Carcass_eQTL,Meat_and_Carcass_QTL,Production_Association,Exterior_Association,Health_Association,Meat_and_Carcass_Association,Health_QTL,Production_QTL,Reproduction_Association,Reproduction_QTL
1	ASGA0003713	84827908	80	84	85	F2	3.926129	0.999957	FAM46A,IBTK,RPL18	Exterior_QTL,Meat_and_Carcass_eQTL,Meat_and_Carcass_QTL,Production_Association,Exterior_Association,Health_Association,Meat_and_Carcass_Association,Health_QTL,Production_QTL,Reproduction_Association,Reproduction_QTL

1	DRGA0001285	85105028	80	85	86	F2	3.921542	0.999956	BCKDHB	Exterior_QTL,Meat_and_Carcass_eQTL,Meat_and_Carcass_QTL,Production_Association,Exterior_Association,Health_Association,Meat_and_Carcass_Association,Health_QTL,Production_QTL,Reproduction_Association,Reproduction_QTL
1	ALGA0113211	86042306	72	86	87	F2	3.410844	0.999676	BCKDHB,ELOVL4,HMGN3,LCA5,SH3BGRL2,TTK	Exterior_QTL,Meat_and_Carcass_eQTL,Meat_and_Carcass_QTL,Production_Association,Reproduction_QTL,Health_Association,Meat_and_Carcass_Association,Health_QTL,Production_QTL,Reproduction_Association,Exterior_Association
2	ALGA0014189	86542827	67	86	87	F2	3.096659	0.999021	AP3B1,OTP,PDE8B,TBCA,WDR41	Exterior_QTL,Meat_and_Carcass_eQTL,Meat_and_Carcass_QTL,Production_Association,Reproduction_QTL,Health_Association,Meat_and_Carcass_Association,Health_QTL,Production_QTL,Reproduction_Association,Exterior_Association
7	ALGA0122542	26976864	73	26	27	F2	3.470267	0.99974	FAM83B,LRRC1,MLIP,TINAG	Exterior_QTL,Meat_and_Carcass_eQTL,Meat_and_Carcass_QTL,Production_Association,Exterior_Association,Health_Association,Reproduction_QTL,Meat_and_Carcass_Association,Health_QTL,Production_QTL,Reproduction_Association

7	ALGA0040000	27707775	74	27	28	F2	3.542629	0.999802	GCLC,KHDRBS2,KLHL31	Exterior_QTL,Meat_and_Carcass_QTL,Meat_and_Carcass_eQTL,Production_Association,Exterior_Association,Health_Association,Reproduction_QTL,Meat_and_Carcass_Association,Health_QTL,Production_QTL,Reproduction_Association
7	H3GA0020623	28383120	71	28	29	F2	3.353231	0.999601	BAG2,BEND6,DST,PRIM2,RAB23,ZNF451	Exterior_QTL,Meat_and_Carcass_QTL,Meat_and_Carcass_eQTL,Production_Association,Exterior_Association,Health_Association,Reproduction_QTL,Meat_and_Carcass_Association,Health_QTL,Production_QTL,Reproduction_Association
7	ASGA0032336	29060950	68	29	30	F2	3.159495	0.99921	B3GALT4,BAK1,C7H6orf125,COL21A1,CUTA,DAXX,DST,IP6K3,ITPR3,KIFC1,LEMD2,LOC100156231,PFDN6,PHF1,RGL2,RPS18,SYNGAP1,TAPBP,VPS52,WDR46,ZBTB22,ZBTB9	Exterior_QTL,Meat_and_Carcass_QTL,Meat_and_Carcass_eQTL,Production_Association,Exterior_Association,Health_Association,Reproduction_QTL,Meat_and_Carcass_Association,Health_QTL,Production_QTL,Reproduction_Association

7	ALGA0041977	50570021	70	50	51	F2	3.289815	0.999499	EFL1,IL16,LOC102160759,MEX3B,SAXO2,STARD5,TMC3	Meat_and_Carcass_eQTL,Meat_and_Carcass_QTL,Production_Association,Exterior_QTL,Health_Association,Reproduction_QTL,Meat_and_Carcass_Association,Health_QTL,Production_QTL,Reproduction_Association,Exterior_Association
7	ASGA0034088	51328974	70	51	52	F2	3.289789	0.999499	ADAMTSL3,BNC1,BTBD1,C7H15orf40,FAM103A1,HDGFL3,SH3GL3,TM6SF1	Meat_and_Carcass_eQTL,Meat_and_Carcass_QTL,Production_Association,Exterior_QTL,Health_Association,Reproduction_QTL,Meat_and_Carcass_Association,Health_QTL,Production_QTL,Reproduction_Association,Exterior_Association
7	ALGA0115326	52709566	70	52	53	F2	3.289828	0.999499	ALPK3,AP3B2,CPEB1,FSD2,HOMER2,NMB,PDE8A,RPS17,SEC11A,SLC28A1,WDR73,WHAMM,ZNF592,ZNF774,ZSCAN2	Meat_and_Carcass_eQTL,Meat_and_Carcass_QTL,Production_Association,Exterior_QTL,Health_Association,Reproduction_QTL,Meat_and_Carcass_Association,Health_QTL,Production_QTL,Reproduction_Association,Exterior_Association
7	ALGA0114757	53234774	70	53	54	F2	3.289452	0.999498	ALDH1L1,BLM,CFAP100,CRTC3,FES,FURIN,HDDC3,IQGAP1,KLF15,MAN2A2,RCCD1,SLC41A3,UNC45A,UROC1	Meat_and_Carcass_eQTL,Meat_and_Carcass_QTL,Production_Association,Exterior_Association,Health_Association,Reproduction_QTL,Meat_and_Carcass_Association,Health_QTL,Production_QTL,Reproduction_Association,Exterior_QTL

									,ZXDC	
9	INRA0031809	47919084	109	47	48	F2	5.769847	1	ARHGEF12,GRIK4,OAF,POU2F3,TMEM136,TRIM29	Meat_and_Carcass_eQTL,Meat_and_Carcass_QTL,Production_Association,Exterior_QTL,Health_Association,Reproduction_QTL,Meat_and_Carcass_Association,Health_QTL,Production_QTL,Reproduction_Association,Exterior_Association
9	MARC0032676	48802206	128	48	49	F2	6.979606	1	GRIK4,LOC100523684,SC5D,SORL1	Meat_and_Carcass_eQTL,Meat_and_Carcass_QTL,Production_Association,Exterior_QTL,Health_Association,Reproduction_QTL,Meat_and_Carcass_Association,Health_QTL,Production_QTL,Reproduction_Association,Exterior_Association
9	ALGA0113380	49590691	128	49	50	F2	6.978203	1	BSX,C9H11orf63,CLMP,CRTAM,HSPA8,LOC102164776,UBASH3B	Meat_and_Carcass_eQTL,Meat_and_Carcass_QTL,Production_Association,Exterior_QTL,Health_Association,Reproduction_QTL,Meat_and_Carcass_Association,Health_QTL,Production_QTL,Reproduction_Association,Exterior_Association

9	ASGA0093525	50162529	82	50	51	F2	4.046456	0.999974	CLMP,GRAMD1B,LOC100512247,LOC100513369,LOC100513559,LOC100515746,LOC100518267,LOC100518623,LOC100518799,LOC100518982,LOC100524388,LOC100524576,LOC100524757,LOC100524947,LOC100525119,LOC100525298,LOC100525476,LOC106504899,LOC106504900,LOC106504901,LOC106504905,LOC106504908,LOC106504911,LOC106504912,LOC110262281,LOC110262293,SCN3B,TMEM225,ZNF202	Meat_and_Carcass_eQTL,Meat_and_Carcass_QTL,Production_Association,Exterior_QTL,Health_Association,Reproduction_QTL,Meat_and_Carcass_Association,Health_QTL,Production_QTL,Reproduction_Association,Exterior_Association

9	ALGA0053212	54910556	84	54	55	F2	4.179668	0.999985	KIRREL3	Meat_and_Carcass_eQTL,Meat_and_Carcass_QTL,Production_Association,Exterior_Association,Health_Association,Reproduction_QTL,Meat_and_Carcass_Association,Health_QTL,Production_QTL,Reproduction_Association,Exterior_QTL
9	MARC0097514	55489578	88	55	56	F2	4.43259	0.999995	ARHGAP32,ETS1,FLI1,KCNJ1,KCNJ5,LOC100516820	Meat_and_Carcass_eQTL,Meat_and_Carcass_QTL,Production_Association,Exterior_Association,Health_Association,Reproduction_QTL,Meat_and_Carcass_Association,Health_QTL,Production_QTL,Reproduction_Association,Exterior_QTL
9	MARC0085499	56626224	89	56	57	F2	4.495752	0.999997	APLP2,ARHGAP32,BARX2,LOC100516455,NFRKB,PRDM10,ST14,TMEM45B,ZBTB44	Meat_and_Carcass_eQTL,Meat_and_Carcass_QTL,Production_Association,Exterior_Association,Health_Association,Reproduction_QTL,Meat_and_Carcass_Association,Health_QTL,Production_QTL,Reproduction_Association,Exterior_QTL
9	ASGA0085584	57093003	89	57	58	F2	4.491663	0.999996	ADAMTS15,ADAMTS8,LOC102165987,NTM,SNX19,ZBTB44	Meat_and_Carcass_eQTL,Meat_and_Carcass_QTL,Production_Association,Exterior_Association,Health_Association,Reproduction_QTL,Meat_and_Carcass_Association,Health_QTL,Production_QTL,Reproduction_Association,Exterior_QTL

14	ALGA0078993	75627113	70	75	76	F2	3.287093	0.999494	ANAPC16, ASCC1,DDIT4,DNAJB12,ECD,FAM149B1,MCU,MICU1,NUDT13,OIT3,P4HA1,PLA2G12B	Exterior_QTL,Meat_and_Carcass_eQTL,Meat_and_Carcass_QTL,Production_Association,Exterior_Association,Health_Association,Meat_and_Carcass_Association,Health_QTL,Production_QTL,Reproduction_Association,Reproduction_QTL
14	DIAS0004346	76070198	70	76	77	F2	3.289458	0.999498	ADK,ANXA7,AP3M1,CAMK2G,CFAP70,CHCHD1,DNAJC9,FAM149B1,FUT11,LOC102160869,LOC106506035,MRPS16,MSS51,MYOZ1,NDST2,PLAU,PPP3CB,SEC24C,SYNPO2L,USP54,VCL,ZSWIM8	Exterior_QTL,Meat_and_Carcass_eQTL,Meat_and_Carcass_QTL,Production_Association,Exterior_Association,Health_Association,Meat_and_Carcass_Association,Health_QTL,Production_QTL,Reproduction_Association,Reproduction_QTL
14	INRA0045050	77399991	70	77	78	F2	3.289914	0.999499	ADK,COMTD1,DUPD1,DUSP13,KAT6B,LOC106507128,SAMD8,VDAC2,ZNF503	Exterior_QTL,Meat_and_Carcass_eQTL,Meat_and_Carcass_QTL,Production_Association,Exterior_Association,Health_Association,Meat_and_Carcass_Association,Health_QTL,Production_QTL,Reproduction_Association,Reproduction_QTL

14	DRGA0014542	118707682	70	118	119	F2	3.288221	0.999496	LOC102162193,SORCS1	Exterior_QTL,Meat_and_Carcass_eQTL,Meat_and_Carcass_QTL,Production_Association,Reproduction_QTL,Health_Association,Meat_and_Carcass_Association,Health_QTL,Production_QTL,Reproduction_Association,Exterior_Association
14	H3GA0042206	119052026	70	119	120	F2	3.289991	0.999499	-	Exterior_QTL,Meat_and_Carcass_eQTL,Meat_and_Carcass_QTL,Production_Association,Exterior_Association,Health_Association,Meat_and_Carcass_Association,Health_QTL,Production_QTL,Reproduction_Association,Reproduction_QTL
14	ASGA0066386	120177202	70	120	121	F2	3.286693	0.999493	ADD3,DUSP5,MXI1,SMC3,SMNDC1,XPNPEP1	Meat_and_Carcass_eQTL,Meat_and_Carcass_QTL,Production_Association,Exterior_Association,Health_Association,Reproduction_QTL,Meat_and_Carcass_Association,Health_QTL,Production_QTL,Reproduction_Association,Exterior_QTL
15	ASGA0104227	27531806	67	27	28	F2	3.098096	0.999026	CNTNAP5	Exterior_QTL,Meat_and_Carcass_QTL,Meat_and_Carcass_eQTL,Production_Association,Exterior_Association,Health_Association,Reproduction_QTL,Meat_and_Carcass_Association,Health_QTL,Production_QTL,Reproduction_Association

16	MARC0043672	18843178	95	18	19	F2	4.879044	0.999999	LOC110257335,MTMR12,NPR3,PDZD2,SUB1,ZFR	Exterior_QTL,Meat_and_Carcass_QTL,Meat_and_Carcass_eQTL,Production_Association,Exterior_Association,Health_Association,Meat_and_Carcass_Association,Health_QTL,Production_QTL,Reproduction_Association,Reproduction_QTL
16	ASGA0100023	19591018	87	19	20	F2	4.371365	0.999994	ADAMTS12,RXFP3,SLC45A2,TARS	Exterior_QTL,Meat_and_Carcass_eQTL,Meat_and_Carcass_QTL,Production_Association,Exterior_Association,Health_Association,Reproduction_QTL,Meat_and_Carcass_Association,Health_QTL,Production_QTL,Reproduction_Association
17	INRA0054314	51956954	88	51	52	F2	4.426038	0.999995	B4GALT5,CEBPB,IL29,KCNB1,LOC100524118,PTGIS,RNF114,SLC9A8,SNAI1,SPATA2,TMEM189,UBE2V1	Meat_and_Carcass_eQTL,Meat_and_Carcass_QTL,Production_Association,Exterior_QTL,Health_Association,Reproduction_QTL,Meat_and_Carcass_Association,Health_QTL,Production_QTL,Reproduction_Association,Exterior_Association
17	H3GA0049440	52287400	88	52	53	F2	4.434547	0.999995	ADNP,ATP9A,BCAS4,DPM1,KCNG1,MOCS3,NFATC2,PARD6B,PTPN1,RIPOR3	Meat_and_Carcass_eQTL,Meat_and_Carcass_QTL,Production_Association,Exterior_QTL,Health_Association,Reproduction_QTL,Meat_and_Carcass_Association,Health_QTL,Production_QTL,Reproduction_Association,Exterior_Association

17	ASGA0077694	53375707	88	53	54	F2	4.434184	0.999995	ATP9A,SALL4,ZFP64	Meat_and_Carcass_eQTL,Meat_and_Carcass_QTL,Production_Association,Exterior_Association,Health_Association,Reproduction_QTL,Meat_and_Carcass_Association,Health_QTL,Production_QTL,Reproduction_Association,Exterior_QTL
17	ALGA0095920	54045554	83	54	55	F2	4.116774	0.999981	BCAS1,TSHZ2,ZNF217	Meat_and_Carcass_eQTL,Meat_and_Carcass_QTL,Production_Association,Exterior_Association,Health_Association,Reproduction_QTL,Meat_and_Carcass_Association,Health_QTL,Production_QTL,Reproduction_Association,Exterior_QTL
1	ALGA0003456	52954585	186	52	53	KNP	2.224625	0.986947	DPPA5,KCNQ5,KHDC3L,LOC100511846,RIMS1	Meat_and_Carcass_eQTL,Meat_and_Carcass_QTL,Production_Association,Exterior_QTL,Health_Association,Reproduction_QTL,Meat_and_Carcass_Association,Health_QTL,Production_QTL,Reproduction_Association,Exterior_Association
1	ASGA0002888	53705976	186	53	54	KNP	2.225822	0.986987	CEP162,DDX43,LOC100622980,MRAP2,OOEP,TBX18	Meat_and_Carcass_eQTL,Meat_and_Carcass_QTL,Production_Association,Exterior_Association,Health_Association,Reproduction_QTL,Meat_and_Carcass_Association,Health_QTL,Production_QTL,Reproduction_Association,Exterior_QTL

1	H3GA0001764	54289787	186	54	55	KNP	2.225361	0.986972	NT5E,SNX14,SYNCRIP	Meat_and_Carcass_eQTL,Meat_and_Carcass_QTL,Production_Association,Exterior_Association,Health_Association,Reproduction_QTL,Meat_and_Carcass_Association,Health_QTL,Production_QTL,Reproduction_Association,Exterior_QTL
1	ALGA0003583	55536765	185	55	56	KNP	2.202758	0.986194	C1H6orf163,CFAP206,CGA,GJB7,HTR1E,LOC100151959,ORC3,RARS2,SLC35A1,SMIM8,ZNF292	Meat_and_Carcass_eQTL,Meat_and_Carcass_QTL,Production_Association,Exterior_Association,Health_Association,Reproduction_QTL,Meat_and_Carcass_Association,Health_QTL,Production_QTL,Reproduction_Association,Exterior_QTL
1	MARC0060278	56780388	194	56	57	KNP	2.40207	0.991849	AKIRIN2,CNR1,LOC100739087,ORC3,RNGTT,SPACA1	Meat_and_Carcass_eQTL,Meat_and_Carcass_QTL,Production_Association,Exterior_Association,Health_Association,Reproduction_QTL,Meat_and_Carcass_Association,Health_QTL,Production_QTL,Reproduction_Association,Exterior_QTL
1	DIAS0001261	109357021	173	109	110	KNP	1.93635	0.973588	C2CD4B,LOC100152206,LOC102164011,TLN2,TPM1,VPS13C	Exterior_QTL,Meat_and_Carcass_eQTL,Meat_and_Carcass_QTL,Production_Association,Reproduction_QTL,Exterior_Association,Health_Association,Meat_and_Carcass_Association,Health_QTL,Production_QTL,Reproduction_Association

1	H3GA0002548	110850133	172	110	111	KNP	1.916082	0.972323	RORA,VPS13C	Exterior_QTL,Meat_and_Carcass_eQTL,Meat_and_Carcass_QTL,Production_Association,Exterior_Association,Health_Association,Meat_and_Carcass_Association,Health_QTL,Production_QTL,Reproduction_Association,Reproduction_QTL
1	ALGA0005558	111905972	172	111	112	KNP	1.915931	0.972313	ANXA2,FOXB1,ICE2,RORA	Exterior_QTL,Meat_and_Carcass_eQTL,Meat_and_Carcass_QTL,Production_Association,Exterior_Association,Health_Association,Meat_and_Carcass_Association,Health_QTL,Production_QTL,Reproduction_Association,Reproduction_QTL
1	MARC0086944	112395309	172	112	113	KNP	1.915672	0.972297	BNIP2,CCNB2,FAM81A,FOXB1,GCNT3,GTF2A2,MYO1E,RNF111	Exterior_QTL,Meat_and_Carcass_eQTL,Meat_and_Carcass_QTL,Production_Association,Exterior_Association,Health_Association,Meat_and_Carcass_Association,Health_QTL,Production_QTL,Reproduction_Association,Reproduction_QTL
1	ALGA0005599	113131546	172	113	114	KNP	1.915777	0.972303	ADAM10,ALDH1A2,AQP9,LIPC,MINDY2,RNF111,SLTM	Exterior_QTL,Meat_and_Carcass_eQTL,Meat_and_Carcass_QTL,Production_Association,Reproduction_QTL,Exterior_Association,Health_Association,Meat_and_Carcass_Association,Health_QTL,Production_QTL,Reproduction_Association

1	H3GA0002618	114628022	172	114	115	KNP	1.915649	0.972295	ALDH1A2,CGNL1,MYZAP,POLR2M,TCF12	Exterior_QTL,Meat_and_Carcass_eQTL,Meat_and_Carcass_QTL,Production_Association,Exterior_Association,Health_Association,Meat_and_Carcass_Association,Health_QTL,Production_QTL,Reproduction_Association,Reproduction_QTL
1	DRGA0001464	115920314	172	115	116	KNP	1.915693	0.972298	MNS1,NEDD4,RFX7,TCF12,TEX9,ZNF280D	Exterior_QTL,Meat_and_Carcass_eQTL,Meat_and_Carcass_QTL,Production_Association,Exterior_Association,Health_Association,Meat_and_Carcass_Association,Health_QTL,Production_QTL,Reproduction_Association,Reproduction_QTL
1	H3GA0002627	116564862	172	116	117	KNP	1.91591	0.972312	C1H15orf65,CCPG1,DNAAF4,NEDD4,PIGB,PIGBOS1,PRTG,PYGO1,RAB27A,RSL24D1	Exterior_QTL,Meat_and_Carcass_eQTL,Meat_and_Carcass_QTL,Production_Association,Exterior_Association,Health_Association,Meat_and_Carcass_Association,Health_QTL,Production_QTL,Reproduction_Association,Reproduction_QTL
1	DRGA0001468	117528483	172	117	118	KNP	1.915593	0.972292	LOC100516308,UNC13C,WDR72	Exterior_QTL,Meat_and_Carcass_eQTL,Meat_and_Carcass_QTL,Production_Association,Exterior_Association,Health_Association,Meat_and_Carcass_Association,Health_QTL,Production_QTL,Reproduction_Association,Reproduction_QTL

1	ALGA0005740	118778245	172	118	119	KNP	1.91627	0.972335	FAM214A,ONECUT1,WDR72	Exterior_QTL,Meat_and_Carcass_eQTL,Meat_and_Carcass_QTL,Production_Association,Reproduction_QTL,Health_Association,Meat_and_Carcass_Association,Health_QTL,Production_QTL,Reproduction_Association,Exterior_Association
1	BGIS0004747	119087100	172	119	120	KNP	1.91609	0.972323	ARPP19,BCL2L10,FAM214A,GNB5,LEO1,LOC102158569,MAPK6,MYO5A,MYO5C,TMOD2,TMOD3	Exterior_QTL,Meat_and_Carcass_eQTL,Meat_and_Carcass_QTL,Production_Association,Exterior_Association,Health_Association,Meat_and_Carcass_Association,Health_QTL,Production_QTL,Reproduction_Association,Reproduction_QTL
1	MARC0028871	120648462	172	120	121	KNP	1.916035	0.97232	AP4E1,CYP19A1,CYP19A2,CYP19A3,DMXL2,GLDN,LOC106508998,LYSMD2,SCG3,TMOD2,TNFAIP8L3	Meat_and_Carcass_eQTL,Meat_and_Carcass_QTL,Production_Association,Exterior_Association,Health_Association,Reproduction_QTL,Meat_and_Carcass_Association,Health_QTL,Production_QTL,Reproduction_Association,Exterior_QTL
1	DRGA0001484	121134328	172	121	122	KNP	1.915625	0.972294	ATP8B4,GABPB1,HDC,SLC27A2,SPPL2A,TRPM7,USP50,USP8	Meat_and_Carcass_eQTL,Meat_and_Carcass_QTL,Production_Association,Exterior_Association,Health_Association,Reproduction_QTL,Meat_and_Carcass_Association,Health_QTL,Production_QTL,Reproduction_Association,Exterior_QTL

1	MARC0065135	122263624	172	122	123	KNP	1.914979	0.972252	CEP152,COPS2,DTWD1,EID1,FAM227B,FGF7,GALK2,SECISBP2L,SHC4	Meat_and_Carcass_eQTL,Meat_and_Carcass_QTL,Production_Association,Exterior_Association,Health_Association,Reproduction_QTL,Meat_and_Carcass_Association,Health_QTL,Production_QTL,Reproduction_Association,Exterior_QTL
1	H3GA0002692	123762641	172	123	124	KNP	1.916158	0.972327	CTXN2,DUT,FBN1,MYEF2,SEMA6D,SLC12A1,SLC24A5	Meat_and_Carcass_eQTL,Meat_and_Carcass_QTL,Production_Association,Exterior_Association,Health_Association,Reproduction_QTL,Meat_and_Carcass_Association,Health_QTL,Production_QTL,Reproduction_Association,Exterior_QTL
1	MARC0031379	124859746	172	124	125	KNP	1.91631	0.972337	SEMA6D	Meat_and_Carcass_eQTL,Meat_and_Carcass_QTL,Production_Association,Exterior_Association,Health_Association,Reproduction_QTL,Meat_and_Carcass_Association,Health_QTL,Production_QTL,Reproduction_Association,Exterior_QTL
1	ASGA0004447	125577948	172	125	126	KNP	1.916178	0.972329	-	Meat_and_Carcass_eQTL,Meat_and_Carcass_QTL,Production_Association,Exterior_Association,Health_Association,Reproduction_QTL,Meat_and_Carcass_Association,Health_QTL,Production_QTL,Reproduction_Association,Exterior_QTL

1	CASI0011475	126252213	173	126	127	KNP	1.937937	0.973685	B2M,BLOC1S6,C1H15orf48,DUOX1,DUOX2,DUOXA1,DUOXA2,EIF3J,GATM,SHF,SLC28A2,SLC30A4,SORD,SPATA5L1,SPG11,SQOR,TERB2,TRIM69	Meat_and_Carcass_QTL,Meat_and_Carcass_eQTL,Production_Association,Reproduction_QTL,Health_Association,Meat_and_Carcass_Association,Health_QTL,Production_QTL,Reproduction_Association,Exterior_QTL
1	H3GA0002734	127890280	173	127	128	KNP	1.938327	0.973708	CASC4,CATSPER2,CKMT1A,CTDSPL2,EIF3J,ELL3,FRMD5,MAP1A,MFAP1,PDIA3,PPIP5K1,SERF2,SERINC4,STRC,TP53BP1,WDR76	Meat_and_Carcass_QTL,Meat_and_Carcass_eQTL,Production_Association,Reproduction_QTL,Exterior_Association,Health_Association,Meat_and_Carcass_Association,Health_QTL,Production_QTL,Reproduction_Association,Exterior_QTL
1	MARC0036466	128156575	173	128	129	KNP	1.938276	0.973705	ADAL,CAPN3,CCNDBP1,CDAN1,EPB42,HAUS2,LCMT2,LRRC57,SNAP23,STARD9,TGM5,TGM7,TMEM62,TP53BP1,TTBK2,TUBGCP4,UBR1,ZNF106,ZSCAN29	Meat_and_Carcass_QTL,Meat_and_Carcass_eQTL,Production_Association,Exterior_QTL,Exterior_Association,Health_Association,Meat_and_Carcass_Association,Health_QTL,Production_QTL,Reproduction_Association,Reproduction_QTL

1	MARC0003250	129217218	173	129	130	KNP	1.938323	0.973708	CAPN3,EHD4,GANC,ITPKA,JMJD7,LOC110256586,LTK,MAPKBP1,MGA,NDUFAF1,NUSAP1,PLA2G4B,PLA2G4D,PLA2G4E,PLA2G4F,RPAP1,RTF1,SPTBN5,TMEM87A,TYRO3,VPS39	Meat_and_Carcass_QTL,Meat_and_Carcass_eQTL,Production_Association,Exterior_QTL,Health_Association,Reproduction_QTL,Meat_and_Carcass_Association,Health_QTL,Production_QTL,Reproduction_Association
1	MARC0054383	130156265	173	130	131	KNP	1.938167	0.973699	BAHD1,C1H15orf52,C1H15orf57,C1H15orf62,CHAC1,CHP1,CHST14,DISP2,DLL4,DNAJC17,EXD1,GCHFR,INAFM2,INO80,IVD,KNL1,KNSTRN,NUSAP1,OIP5,PPP1R14D,RAD51,RHOV,RMDN3,RPUSD2,SPINT1,VPS18,ZFYVE19	Meat_and_Carcass_QTL,Meat_and_Carcass_eQTL,Production_Association,Exterior_QTL,Health_Association,Meat_and_Carcass_Association,Health_QTL,Production_QTL,Reproduction_Association,Reproduction_QTL

1	MARC0031936	131196781	173	131	132	KNP	1.938238	0.973703	ANKRD63,BMF,BUB1B,EIF2AK4,FSIP1,GPR176,PAK6,PLCB2,SRP14,THBS1	Meat_and_Carcass_QTL,Meat_and_Carcass_eQTL,Production_Association,Exterior_QTL,Exterior_Association,Health_Association,Meat_and_Carcass_Association,Health_QTL,Production_QTL,Reproduction_Association,Reproduction_QTL
1	ALGA0006043	132481475	173	132	133	KNP	1.938343	0.973709	FAM98B,LOC110259514,RASGRP1,SPRED1	Meat_and_Carcass_QTL,Meat_and_Carcass_eQTL,Production_Association,Exterior_QTL,Exterior_Association,Health_Association,Meat_and_Carcass_Association,Health_QTL,Production_QTL,Reproduction_Association,Reproduction_QTL
1	ASGA0004619	133936285	173	133	134	KNP	1.938302	0.973707	LOC106506784,SPRED1,TMCO5A	Meat_and_Carcass_QTL,Meat_and_Carcass_eQTL,Production_Association,Exterior_QTL,Health_Association,Meat_and_Carcass_Association,Health_QTL,Production_QTL,Reproduction_Association,Reproduction_QTL
1	INRA0004288	134594000	173	134	135	KNP	1.937997	0.973688	C1H15orf41,LOC110259520,MEIS2	Meat_and_Carcass_QTL,Meat_and_Carcass_eQTL,Production_Association,Reproduction_QTL,Exterior_Association,Health_Association,Meat_and_Carcass_Association,Health_QTL,Production_QTL,Reproduction_Association,Exterior_QTL

1	INRA0004304	135322151	173	135	136	KNP	1.937905	0.973683	DPH6	Meat_and_Carcass_QTL,Meat_and_Carcass_eQTL,Production_Association,Exterior_QTL,Exterior_Association,Health_Association,Meat_and_Carcass_Association,Health_QTL,Production_QTL,Reproduction_QTL
1	ALGA0006090	136457236	173	136	137	KNP	1.937904	0.973683	ACTC1,AQR,ARHGAP11A,DPH6,FMN1,GJD2,GREM1,SCG5,ZNF770	Meat_and_Carcass_QTL,Meat_and_Carcass_eQTL,Production_Association,Reproduction_QTL,Exterior_Association,Health_Association,Meat_and_Carcass_Association,Health_QTL,Production_QTL,Reproduction_Association,Exterior_QTL
1	MARC0098235	137550210	173	137	138	KNP	1.938404	0.973713	FAM169B,FMN1,IGF1R,LRRC28,PGPEP1L,SYNM,TTC23	Meat_and_Carcass_QTL,Meat_and_Carcass_eQTL,Production_Association,Reproduction_QTL,Exterior_Association,Health_Association,Meat_and_Carcass_Association,Health_QTL,Production_QTL,Reproduction_Association,Exterior_QTL
1	ASGA0085220	138837899	173	138	139	KNP	1.938107	0.973695	ADAMTS17,LRRC28,LYSMD4,MEF2A	Meat_and_Carcass_QTL,Meat_and_Carcass_eQTL,Production_Association,Exterior_QTL,Exterior_Association,Health_Association,Meat_and_Carcass_Association,Health_QTL,Production_QTL,Reproduction_Association,Reproduction_QTL

1	ALGA0006121	139207599	173	139	140	KNP	1.937907	0.973683	ADAMTS17,ALDH1A3,ASB7,CERS3,CHSY1,GABRG3,LINS1,LOC106509041,LRRK1,SELENOS	Meat_and_Carcass_eQTL,Meat_and_Carcass_QTL,Production_Association,Exterior_QTL,Exterior_Association,Health_Association,Reproduction_QTL,Meat_and_Carcass_Association,Health_QTL,Production_QTL,Reproduction_Association
1	ALGA0006191	140163320	173	140	141	KNP	1.938247	0.973703	GABRA5,GABRB3,GABRG3	Meat_and_Carcass_eQTL,Meat_and_Carcass_QTL,Production_Association,Exterior_QTL,Exterior_Association,Health_Association,Reproduction_QTL,Meat_and_Carcass_Association,Health_QTL,Production_QTL,Reproduction_Association
1	MARC0019893	141068209	173	141	142	KNP	1.938256	0.973704	ATP10A,UBE3A	Meat_and_Carcass_QTL,Meat_and_Carcass_eQTL,Production_Association,Exterior_QTL,Exterior_Association,Health_Association,Reproduction_QTL,Meat_and_Carcass_Association,Health_QTL,Production_QTL,Reproduction_Association
1	ALGA0006239	142426592	173	142	143	KNP	1.937519	0.973659	CHRNA7,LOC102163334,MAGEL2,MKRN3,NDN,UBE3A	Meat_and_Carcass_QTL,Meat_and_Carcass_eQTL,Production_Association,Reproduction_QTL,Exterior_Association,Health_Association,Meat_and_Carcass_Association,Health_QTL,Production_QTL,Reproduction_Association,Exterior_QTL

1	INRA0004493	143981641	173	143	144	KNP	1.938346	0.973709	FAN1,KLF13,LOC110259530,MCEE,MPHOSPH10,MTMR10,OTUD7A,TRPM1	Meat_and_Carcass_QTL,Meat_and_Carcass_eQTL,Production_Association,Reproduction_QTL,Exterior_Association,Health_Association,Meat_and_Carcass_Association,Health_QTL,Production_QTL,Reproduction_Association,Exterior_QTL
1	MARC0072630	144803760	173	144	145	KNP	1.937165	0.973637	APBA2,FAM189A1,MCEE,NSMCE3,TJP1	Meat_and_Carcass_eQTL,Meat_and_Carcass_QTL,Production_Association,Reproduction_QTL,Exterior_Association,Health_Association,Meat_and_Carcass_Association,Health_QTL,Production_QTL,Reproduction_Association,Exterior_QTL
1	INRA0007995	145741148	173	145	146	KNP	1.938012	0.973689	CTDP1,NFATC1,PCSK6,SEH1L,SNRPA1,TARSL2,TJP1,TM2D3	Meat_and_Carcass_QTL,Meat_and_Carcass_eQTL,Production_Association,Reproduction_Association,Exterior_Association,Health_Association,Reproduction_QTL,Meat_and_Carcass_Association,Health_QTL,Production_QTL,Exterior_QTL
1	MARC0073315	146384107	172	146	147	KNP	1.916178	0.972329	ATP9B,NFATC1,SALL3	Meat_and_Carcass_QTL,Meat_and_Carcass_eQTL,Production_Association,Reproduction_Association,Health_Association,Reproduction_QTL,Meat_and_Carcass_Association,Health_QTL,Production_QTL,Exterior_QTL

1	M1GA0001154	147569477	172	147	148	KNP	1.91625	0.972333	GALR1,MBP,ZNF236,ZNF516	Meat_and_Carcass_QTL,Meat_and_Carcass_eQTL,Production_Association,Reproduction_QTL,Exterior_Association,Health_Association,Meat_and_Carcass_Association,Health_QTL,Production_QTL,Exterior_QTL
1	ASGA0004881	148527478	172	148	149	KNP	1.9159	0.972311	TSHZ1,ZADH2,ZNF516	Meat_and_Carcass_QTL,Meat_and_Carcass_eQTL,Production_Association,Reproduction_QTL,Exterior_Association,Health_Association,Meat_and_Carcass_Association,Health_QTL,Production_QTL,Reproduction_Association,Exterior_QTL
1	ASGA0004902	149967322	172	149	150	KNP	1.915657	0.972296	C1H18orf63,CNDP1,CNDP2,CYB5A,FAM69C,FBXO15,TIMM21,ZNF407	Meat_and_Carcass_QTL,Meat_and_Carcass_eQTL,Production_Association,Reproduction_QTL,Exterior_Association,Health_Association,Meat_and_Carcass_Association,Health_QTL,Production_QTL,Reproduction_Association,Exterior_QTL
1	ASGA0101470	150051346	172	150	151	KNP	1.915786	0.972304	CBLN2,NETO1	Meat_and_Carcass_QTL,Meat_and_Carcass_eQTL,Production_Association,Reproduction_QTL,Exterior_Association,Health_Association,Meat_and_Carcass_Association,Health_QTL,Production_QTL,Exterior_QTL

1	ALGA0006467	151342109	164	151	152	KNP	1.739213	0.959001	-	Meat_and_Carcass_QTL,Meat_and_Carcass_eQTL,Production_Association,Exterior_QTL,Health_Association,Meat_and_Carcass_Association,Health_QTL,Production_QTL,Reproduction_Association,Reproduction_QTL
1	INRA0004759	152376522	164	152	153	KNP	1.738834	0.958968	CD226,DOK6,RTTN,SOCS6	Meat_and_Carcass_QTL,Meat_and_Carcass_eQTL,Production_Association,Reproduction_QTL,Health_Association,Meat_and_Carcass_Association,Health_QTL,Production_QTL,Exterior_QTL
1	ALGA0006496	153127121	164	153	154	KNP	1.738699	0.958956	CCDC102B,DOK6,TMX3	Meat_and_Carcass_QTL,Meat_and_Carcass_eQTL,Reproduction_QTL,Meat_and_Carcass_Association,Health_QTL,Production_QTL,Exterior_QTL
1	INRA0004795	154272344	162	154	155	KNP	1.694999	0.954962	DSEL,TMX3	Meat_and_Carcass_QTL,Meat_and_Carcass_eQTL,Production_Association,Reproduction_QTL,Exterior_Association,Meat_and_Carcass_Association,Health_QTL,Production_QTL,Exterior_QTL
1	ALGA0006539	155986286	165	155	156	KNP	1.759324	0.960739	CDH19	Meat_and_Carcass_QTL,Meat_and_Carcass_eQTL,Production_Association,Exterior_QTL,Meat_and_Carcass_Association,Health_QTL,Production_QTL,Reproduction_Association,Reproduction_QTL

1	ASGA0004946	156293498	165	156	157	KNP	1.76114	0.960893	CDH7	Meat_and_Carcass_QTL,Meat_and_Carcass_eQTL,Production_Association,Reproduction_QTL,Exterior_Association,Meat_and_Carcass_Association,Health_QTL,Production_QTL,Reproduction_Association,Exterior_QTL
1	ASGA0004953	157498487	165	157	158	KNP	1.761454	0.960919	LOC100153783,LOC100156248,SERPINB10,SERPINB2,SERPINB7	Meat_and_Carcass_QTL,Meat_and_Carcass_eQTL,Production_Association,Reproduction_QTL,Health_Association,Meat_and_Carcass_Association,Health_QTL,Production_QTL,Exterior_QTL
1	MARC0034873	158682904	165	158	159	KNP	1.76064	0.96085	BCL2,KDSR,LOC110261633,LOC110261636,LOC110261637,PHLPP1,SERPINB11,SERPINB12,SERPINB13,SERPINB5,VPS4B,ZCCHC2	Meat_and_Carcass_QTL,Meat_and_Carcass_eQTL,Production_Association,Exterior_QTL,Exterior_Association,Health_Association,Meat_and_Carcass_Association,Health_QTL,Production_QTL,Reproduction_QTL
1	ASGA0106369	159869511	165	159	160	KNP	1.759849	0.960783	CDH20,KIAA1468,PIGN,RNF152,TNFRSF11A,ZCCHC2	Meat_and_Carcass_QTL,Meat_and_Carcass_eQTL,Production_Association,Exterior_QTL,Exterior_Association,Health_Association,Meat_and_Carcass_Association,Health_QTL,Production_QTL,Reproduction_Association,Reproduction_QTL

1	ALGA0006628	160477079	165	160	161	KNP	1.761248	0.960902	CDH20,LOC110261667,MC4R	Meat_and_Carcass_eQTL,Meat_and_Carcass_QTL,Production_Association,Reproduction_Association,Exterior_Association,Health_Association,Reproduction_QTL,Meat_and_Carcass_Association,Health_QTL,Production_QTL,Exterior_QTL
1	DIAS0002061	161757995	166	161	162	KNP	1.783263	0.962728	CCBE1,CPLX4,GRP,LMAN1,LOC100627702,PMAIP1,RAX,SEC11C	Meat_and_Carcass_QTL,Meat_and_Carcass_eQTL,Production_Association,Exterior_QTL,Health_Association,Meat_and_Carcass_Association,Health_QTL,Production_QTL,Reproduction_QTL
1	MARC0093471	162041614	166	162	163	KNP	1.782598	0.962674	ALPK2,ATP8B1,MALT1,NEDD4L	Meat_and_Carcass_QTL,Meat_and_Carcass_eQTL,Production_Association,Exterior_QTL,Exterior_Association,Health_Association,Meat_and_Carcass_Association,Health_QTL,Production_QTL,Reproduction_QTL
1	MARC0081465	163855473	181	163	164	KNP	2.115223	0.982795	ATP8B1,CILP,CLPX,DENND4A,DPP8,HACD3,IGDCC3,IGDCC4,INTS14,KBTBD13,MEGF11,PARP16,PDCD7,RAB11A,RASL12,SLC24A1,SLC51B,UBAP1	Meat_and_Carcass_QTL,Meat_and_Carcass_eQTL,Production_Association,Exterior_QTL,Health_Association,Reproduction_QTL,Meat_and_Carcass_Association,Health_QTL,Production_QTL,Reproduction_Association,Exterior_Association

									L	
1	ASGA0005108	164023885	181	164	165	KNP	2.115347	0.9828	DIS3L,LCTL,MAP2K1,MEGF11,RPL4,SMAD3,SMAD6,SNAPC5,TIPIN,ZWILCH	Meat_and_Carcass_QTL,Meat_and_Carcass_eQTL,Exterior_QTL,Health_Association,Reproduction_QTL,Health_QTL,Production_QTL,Reproduction_Association,Exterior_Association
1	H3GA0003202	165756649	181	165	166	KNP	2.114794	0.982776	AAGAB,C1H15orf61,IQCH,MAP2K5,PIAS1,SKOR1,SMAD3	Meat_and_Carcass_QTL,Meat_and_Carcass_eQTL,Production_Association,Exterior_QTL,Exterior_Association,Health_Association,Meat_and_Carcass_Association,Health_QTL,Production_QTL,Reproduction_QTL
1	MARC0053979	166608531	181	166	167	KNP	2.115162	0.982792	ANP32A,CALML4,CLN6,CORO2B,FEM1B,ITGA11,NOX5,PIAS1,SPESP1	Meat_and_Carcass_QTL,Meat_and_Carcass_eQTL,Production_Association,Reproduction_Association,Exterior_Association,Health_Association,Reproduction_QTL,Meat_and_Carcass_Association,Health_QTL,Production_QTL,Exterior_QTL

1	ALGA0006886	167546126	181	167	168	KNP	2.115154	0.982792	GLCE,KIF23,PAQR5,RPLP1,TLE3	Meat_and_Carcass_QTL,Meat_and_Carcass_eQTL,Production_Association,Exterior_QTL,Exterior_Association,Health_Association,Reproduction_QTL,Meat_and_Carcass_Association,Health_QTL,Production_QTL,Reproduction_Association
1	H3GA0003311	168036981	181	168	169	KNP	2.115253	0.982796	LARP6,LRRC49,THSD4,UACA	Meat_and_Carcass_QTL,Meat_and_Carcass_eQTL,Production_Association,Exterior_QTL,Exterior_Association,Health_Association,Reproduction_QTL,Meat_and_Carcass_Association,Health_QTL,Production_QTL,Reproduction_Association

1	INRA0007578	263682954	187	263	264	KNP	2.246623	0.987668	LOC100520823,LOC100521356,LOC100626932, LOC100737820,LOC110257105,LOC110257108, LOC110257110,LOC110257111,LOC110257114,LOC110257118,LOC110259686,LOC110259694,LOC110259697,LOC110259699,LOC110259704,LOC110259713,LOC396787,PDCL,RABGAP1,RC3H2,ZBTB26,ZBTB6	Meat_and_Carcass_QTL,Meat_and_Carcass_eQTL,Reproduction_QTL,Meat_and_Carcass_Association,Health_QTL,Production_QTL,Reproduction_Association,Exterior_QTL
1	MARC0096868	264522320	187	264	265	KNP	2.24807	0.987714	CRB2,DENND1A,LHX2,RABGAP1,STRBP	Meat_and_Carcass_QTL,Meat_and_Carcass_eQTL,Reproduction_Association,Reproduction_QTL,Meat_and_Carcass_Association,Health_QTL,Production_QTL,Exterior_QTL

1	ASGA0007588	265031489	187	265	266	KNP	2.247552	0.987698	ADGRD2,ARPC5L,GAPVD1,GOLGA1,HSPA5,NEK6,NR5A1,NR6A1,OLFML2A,PPP6C,PSMB7,RABEPK,RPL35,SCAI,WDR38	Meat_and_Carcass_QTL,Meat_and_Carcass_eQTL,Production_Association,Exterior_QTL,Exterior_Association,Meat_and_Carcass_Association,Health_QTL,Production_QTL,Reproduction_QTL
1	MARC0114865	266074469	186	266	267	KNP	2.225765	0.986985	GAPVD1,LOC106507123,MAPKAP1,MVB12B,PBX3	Meat_and_Carcass_QTL,Meat_and_Carcass_eQTL,Reproduction_QTL,Exterior_Association,Health_QTL,Production_QTL,Exterior_QTL
1	INRA0007636	267549835	185	267	268	KNP	2.203174	0.986209	ANGPTL2,GARNL3,LMX1B,LOC102168098,LRSAM1,MVB12B,RALGPS1,RPL12,SLC2A8,ZBTB34,ZBTB43,ZNF79	Meat_and_Carcass_QTL,Meat_and_Carcass_eQTL,Production_Association,Exterior_QTL,Health_Association,Health_QTL,Production_QTL,Reproduction_QTL

1	M1GA0001651	268000680	185	268	269	KNP	2.201133	0.986137	AK1,C1H9orf16,CDK9,CERCAM,CFAP157,CIZ1,COQ4,DNM1,DPM2,ENG,FAM129B,FPGS,GLE1,GOLGA2,LCN2,LRSAM1,NAIF1,ODF2,PIP5KL1,PTGES2,PTRH1,RSC1A1,SH2D3C,SLC25A25,SLC27A4,SPTAN1,ST6GALNAC4,ST6GALNAC6,STXBP1,TOR2A,TRUB2,TTC16,URM1	Meat_and_Carcass_QTL,Meat_and_Carcass_eQTL,Production_Association,Exterior_QTL,Health_QTL,Production_QTL,Reproduction_Association,Reproduction_QTL

1	ASGA0007850	269264772	175	269	270	KNP	1.982352	0.97628	ASB6,C1H9orf50,C1H9orf78,CRAT,DOLK,DOLPP1,ENDOG,IER5L,KYAT1,LOC110256380,LRRC8A,MIGA2,NTMT1,NUP188,PHYHD1,PKN3,PRRX2,PTGES,PTPA,SET,SH3GLB2,SPOUT1,SPTAN1,TBC1D13,TOR1A,TOR1B,USP20,WDR34,ZDHHC12,ZER1	Meat_and_Carcass_QTL,Meat_and_Carcass_eQTL,Production_Association,Exterior_QTL,Exterior_Association,Health_Association,Health_QTL,Production_QTL,Reproduction_Association,Reproduction_QTL

5	ASGA0104918	63358816	166	63	64	KNP	1.782789	0.96269	ATN1,C1R,C1RL,C1S,C3AR1,C5H12orf57,CD163,CD163L1,CD4,CDCA3,CLEC4E,CLSTN3,COPS7A,EMG1,ENO2,FOXJ2,GNB3,GPR162,LAG3,LOC110260588,LOC110260589,LOC110260590,LOC110260747,LOC110260749,LPCAT3,LRRC23,MLF2,NECAP1,P3H3,PEX5,PHB2,PIANP,PTMS,PTPN6,RBP5,SLC2A3,SPSB2,TPI1,USP5	Exterior_QTL,Meat_and_Carcass_eQTL,Meat_and_Carcass_QTL,Production_Association,Reproduction_QTL,Health_Association,Meat_and_Carcass_Association,Health_QTL,Production_QTL,Reproduction_Association,Exterior_Association

5	MARC0056196	64489653	163	64	65	KNP	1.717027	0.957013	ACRBP,ANO2,CD27,CD9,CHD4,GAPDH,IFFO1,ING4,LPAR5,LTBR,MRPL51,NCAPD2,NOP2,PLEKHG6,SCNN1A,TAPBPL,TNFRSF1A,VAMP1,VWF,ZNF384	Exterior_QTL,Meat_and_Carcass_eQTL,Meat_and_Carcass_QTL,Production_Association,Exterior_Association,Health_Association,Meat_and_Carcass_Association,Health_QTL,Production_QTL,Reproduction_Association,Reproduction_QTL
5	ALGA0032465	65788866	177	65	66	KNP	2.026619	0.978649	AKAP3,ANO2,C5H12orf4,DYRK4,FGF6,GALNT8,KCNA1,KCNA5,KCNA6,NDUFA9,NTF3,RAD51AP1	Exterior_QTL,Meat_and_Carcass_eQTL,Meat_and_Carcass_QTL,Production_Association,Exterior_Association,Health_Association,Meat_and_Carcass_Association,Health_QTL,Production_QTL,Reproduction_Association,Reproduction_QTL
5	MARC0051602	66176510	177	66	67	KNP	2.026644	0.978651	CCND2,CRACR2A,FGF23,PARP11,PRMT8,TIGAR,TSPAN11,TSPAN9	Exterior_QTL,Meat_and_Carcass_eQTL,Meat_and_Carcass_QTL,Production_Association,Exterior_Association,Health_Association,Meat_and_Carcass_Association,Health_QTL,Production_QTL,Reproduction_Association,Reproduction_QTL

5	MARC0103524	67100119	177	67	68	KNP	2.026869	0.978662	B4GALNT3,CCDC77,DDX11,FKBP4,FOXM1,IQSEC3,ITFG2,KDM5A,LOC100515379,LOC110260591,NRIP2,RHNO1,SLC6A12,SLC6A13,TEAD4,TSPAN9,TULP3,WASHC1	Exterior_QTL,Meat_and_Carcass_eQTL,Meat_and_Carcass_QTL,Production_Association,Exterior_Association,Health_Association,Meat_and_Carcass_Association,Health_QTL,Production_QTL,Reproduction_Association,Reproduction_QTL
5	ALGA0103670	77926937	169	77	78	KNP	1.848339	0.967723	AMIGO2,LOC106510389,LOC110260812,RPAP3,SLC38A1,SLC38A2,SLC38A4	Exterior_QTL,Meat_and_Carcass_eQTL,Meat_and_Carcass_QTL,Production_Association,Exterior_Association,Health_Association,Meat_and_Carcass_Association,Health_QTL,Production_QTL,Reproduction_Association,Reproduction_QTL

5	MARC0009241	78029583	169	78	79	KNP	1.849874	0.967834	ASB8,C5H12orf54,CCDC184,COL2A1,ENDOU,HDAC7,LOC100155591,LOC100156793,LOC100516553,LOC100516735,LOC100516910,LOC100517094,LOC100517469,LOC100621423,LOC102161787,LOC102167685,LOC106510393,LOC110260592,LOC110260593,PFKM,RAPGEF3,RPAP3,SENP1,SLC48A1,TMEM106C,VDR,ZNF641	Exterior_QTL,Meat_and_Carcass_eQTL,Meat_and_Carcass_QTL,Production_Association,Exterior_Association,Health_Association,Meat_and_Carcass_Association,Health_QTL,Production_QTL,Reproduction_Association,Reproduction_QTL
7	ALGA0044383	105954725	167	105	106	KNP	1.803812	0.96437	LOC100152218,LOC102165146	Exterior_QTL,Meat_and_Carcass_eQTL,Meat_and_Carcass_QTL,Production_Association,Reproduction_QTL,Health_Association,Meat_and_Carcass_Association,Health_QTL,Production_QTL,Reproduction_Association,Exterior_Association

7	ALGA0044414	106555323	194	106	107	KNP	2.396614	0.991726	-	Exterior_QTL,Meat_and_Carcass_eQTL,Meat_and_Carcass_QTL,Production_Association,Reproduction_QTL,Health_Association,Meat_and_Carcass_Association,Health_QTL,Production_QTL,Reproduction_Association,Exterior_Association
7	ALGA0044461	107628805	198	107	108	KNP	2.491175	0.993634	FLRT2	Exterior_QTL,Meat_and_Carcass_eQTL,Meat_and_Carcass_QTL,Production_Association,Reproduction_QTL,Health_Association,Meat_and_Carcass_Association,Health_QTL,Production_QTL,Reproduction_Association,Exterior_Association
7	ALGA0102373	108133393	198	108	109	KNP	2.491261	0.993635	-	Exterior_QTL,Meat_and_Carcass_eQTL,Meat_and_Carcass_QTL,Production_Association,Exterior_Association,Health_Association,Meat_and_Carcass_Association,Health_QTL,Production_QTL,Reproduction_Association,Reproduction_QTL
7	ALGA0044612	109748775	198	109	110	KNP	2.491426	0.993638	GALC,GPR65	Exterior_QTL,Meat_and_Carcass_eQTL,Meat_and_Carcass_QTL,Production_Association,Reproduction_QTL,Exterior_Association,Health_Association,Meat_and_Carcass_Association,Health_QTL,Production_QTL,Reproduction_Association

7	DRGA0008172	110734474	197	110	111	KNP	2.469307	0.993231	EML5,KCNK10,PTPN21,SPATA7,TTC8,ZC3H14	Exterior_QTL,Meat_and_Carcass_eQTL,Meat_and_Carcass_QTL,Production_Association,Exterior_Association,Health_Association,Meat_and_Carcass_Association,Health_QTL,Production_QTL,Reproduction_Association,Reproduction_QTL
7	ALGA0044742	111193274	186	111	112	KNP	2.220209	0.986798	EFCAB11,FOXN3,KCNK13,LOC106504536,TDP1	Exterior_QTL,Meat_and_Carcass_eQTL,Meat_and_Carcass_QTL,Production_Association,Exterior_Association,Health_Association,Meat_and_Carcass_Association,Health_QTL,Production_QTL,Reproduction_Association,Reproduction_QTL
8	ASGA0089759	81966981	186	81	82	KNP	2.221691	0.986848	ARHGAP10,EDNRA,PRMT9,TMEM184C,TTC29	Exterior_QTL,Meat_and_Carcass_eQTL,Meat_and_Carcass_QTL,Production_Association,Exterior_Association,Health_Association,Meat_and_Carcass_Association,Health_QTL,Production_QTL,Reproduction_Association,Reproduction_QTL
8	MARC0040474	82745550	186	82	83	KNP	2.225716	0.986983	LOC100523624,LOC102167861,LSM6,POU4F2,SLC10A7,SMAD1,ZNF827	Exterior_QTL,Meat_and_Carcass_eQTL,Meat_and_Carcass_QTL,Production_Association,Exterior_Association,Health_Association,Meat_and_Carcass_Association,Health_QTL,Production_QTL,Reproduction_Association,Reproduction_QTL

8	ALGA0109028	83231365	186	83	84	KNP	2.225519	0.986977	ABCE1,ANAPC10,HHIP,LOC100525237,OTUD4	Exterior_QTL,Meat_and_Carcass_eQTL,Meat_and_Carcass_QTL,Production_Association,Exterior_Association,Health_Association,Meat_and_Carcass_Association,Health_QTL,Production_QTL,Reproduction_Association,Reproduction_QTL
8	DBWU0000655	84101692	186	84	85	KNP	2.225311	0.98697	FREM3,GAB1,GYPA,INPP4B,SMARCA5,USP38	Exterior_QTL,Meat_and_Carcass_eQTL,Meat_and_Carcass_QTL,Production_Association,Exterior_Association,Health_Association,Meat_and_Carcass_Association,Health_QTL,Production_QTL,Reproduction_Association,Reproduction_QTL
9	DRGA0009348	40348548	160	40	41	KNP	1.649654	0.950493	NCAM1,TTC12	Meat_and_Carcass_eQTL,Meat_and_Carcass_QTL,Production_Association,Exterior_QTL,Health_Association,Reproduction_QTL,Meat_and_Carcass_Association,Health_QTL,Production_QTL,Reproduction_Association,Exterior_Association

9	ASGA0042984	46519348	177	46	47	KNP	2.026281	0.978632	ABCG4,BCL9L,C1QTNF5,C2CD2L,CBL,CCDC153,CCDC84,CXCR5,DDX6,DPAGT1,FOXR1,HINFP,HMBS,HYOU1,LOC100522201,LOC106504894,MCAM,MFRP,NECTIN1,NLRX1,PDZD3,RNF26,RPS25,SLC37A4,THY1,TRAPPC4,UPK2,USP2,VPS11	Meat_and_Carcass_eQTL,Meat_and_Carcass_QTL,Production_Association,Exterior_QTL,Health_Association,Reproduction_QTL,Meat_and_Carcass_Association,Health_QTL,Production_QTL,Reproduction_Association,Exterior_Association
9	ASGA0082366	47037744	177	47	48	KNP	2.025303	0.978582	ARHGEF12,GRIK4,OAF,POU2F3,TMEM136,TRIM29	Meat_and_Carcass_eQTL,Meat_and_Carcass_QTL,Production_Association,Exterior_QTL,Health_Association,Reproduction_QTL,Meat_and_Carcass_Association,Health_QTL,Production_QTL,Reproduction_Association,Exterior_Association

9	ALGA0052981	48459125	176	48	49	KNP	2.004714	0.977503	GRIK4,LOC100523684,SC5D,SORL1	Meat_and_Carcass_eQTL,Meat_and_Carcass_QTL,Production_Association,Exterior_QTL,Health_Association,Reproduction_QTL,Meat_and_Carcass_Association,Health_QTL,Production_QTL,Reproduction_Association,Exterior_Association
9	H3GA0027351	49043229	176	49	50	KNP	2.004462	0.97749	BSX,C9H11orf63,CLMP,CRTAM,HSPA8,LOC102164776,UBASH3B	Meat_and_Carcass_eQTL,Meat_and_Carcass_QTL,Production_Association,Exterior_QTL,Health_Association,Reproduction_QTL,Meat_and_Carcass_Association,Health_QTL,Production_QTL,Reproduction_Association,Exterior_Association

9	ASGA0102337	50457899	176	50	51	KNP	2.004721	0.977504	CLMP,GRAMD1B,LOC100512247,LOC100513369,LOC100513559,LOC100515746,LOC100518267,LOC100518623,LOC100518799,LOC100518982,LOC100524388,LOC100524576,LOC100524757,LOC100524947,LOC100525119,LOC100525298,LOC100525476,LOC106504899,LOC106504900,LOC106504901,LOC106504905,LOC106504908,LOC106504911,LOC106504912,LOC110262281,LOC110262293,SCN3B,TMEM225,ZNF202	Meat_and_Carcass_eQTL,Meat_and_Carcass_QTL,Production_Association,Exterior_QTL,Health_Association,Reproduction_QTL,Meat_and_Carcass_Association,Health_QTL,Production_QTL,Reproduction_Association,Exterior_Association

9	H3GA0027383	51077971	176	51	52	KNP	1.999074	0.9772	ESAM,LOC100511340,LOC100513751,LOC100513942,LOC100514309,LOC100514671,LOC100515044,LOC100516283,LOC100516454,LOC100516632,LOC100516819,LOC100516990,LOC100519165,LOC100519326,LOC100519506,LOC100519677,LOC100519859,LOC100520042,LOC100520398,LOC100520926,LOC100521109,LOC100521287,LOC100523744,LOC100523913,LOC100524098,LOC100524268,LOC100623188,LOC100625357,LOC100625454,LOC100625	Meat_and_Carcass_eQTL,Meat_and_Carcass_QTL,Production_Association,Exterior_QTL,Health_Association,Reproduction_QTL,Meat_and_Carcass_Association,Health_QTL,Production_QTL,Reproduction_Association,Exterior_Association

									762,LOC100625852,LOC100625943,LOC100737292,LOC100739156,LOC102157846,LOC106504915,LOC110255559,LOC110262283,LOC110262284,LOC110262285,LOC110262286,LOC110262287,LOC110262288,LOC110262289,MSANTD2,NRGN,PANX3,SIAE,SPA17,TBRG1,VSIG2,VWA5A	

9	H3GA0027462	55409686	183	55	56	KNP	2.157263	0.984507	ARHGAP32,ETS1,FLI1,KCNJ1,KCNJ5,LOC100516820	Meat_and_Carcass_eQTL,Meat_and_Carcass_QTL,Production_Association,Exterior_Association,Health_Association,Reproduction_QTL,Meat_and_Carcass_Association,Health_QTL,Production_QTL,Reproduction_Association,Exterior_QTL
9	ALGA0053271	56343466	182	56	57	KNP	2.137385	0.983717	APLP2,ARHGAP32,BARX2,LOC100516455,NFRKB,PRDM10,ST14,TMEM45B,ZBTB44	Meat_and_Carcass_eQTL,Meat_and_Carcass_QTL,Production_Association,Exterior_Association,Health_Association,Reproduction_QTL,Meat_and_Carcass_Association,Health_QTL,Production_QTL,Reproduction_Association,Exterior_QTL
9	H3GA0055699	57097854	182	57	58	KNP	2.136591	0.983684	ADAMTS15,ADAMTS8,LOC102165987,NTM,SNX19,ZBTB44	Meat_and_Carcass_eQTL,Meat_and_Carcass_QTL,Production_Association,Exterior_Association,Health_Association,Reproduction_QTL,Meat_and_Carcass_Association,Health_QTL,Production_QTL,Reproduction_Association,Exterior_QTL
9	ALGA0053362	58267901	181	58	59	KNP	2.114912	0.982781	NTM	Meat_and_Carcass_eQTL,Meat_and_Carcass_QTL,Production_Association,Exterior_Association,Health_Association,Reproduction_QTL,Meat_and_Carcass_Association,Health_QTL,Production_QTL,Reproduction_Association,Exterior_QTL

9	ASGA0105352	59332289	181	59	60	KNP	2.115204	0.982794	OPCML	Meat_and_Carcass_eQTL,Meat_and_Carcass_QTL,Production_Association,Exterior_Association,Health_Association,Reproduction_QTL,Meat_and_Carcass_Association,Health_QTL,Production_QTL,Reproduction_Association,Exterior_QTL
9	ASGA0084646	60623732	181	60	61	KNP	2.113839	0.982735	ACAD8,B3GAT1,GLB1L3,IGSF9B,JAM3,LOC100520582,LOC110255463,NCAPD3,OPCML,SPATA19,THYN1,VPS26B	Exterior_QTL,Meat_and_Carcass_eQTL,Meat_and_Carcass_QTL,Production_Association,Exterior_Association,Health_Association,Meat_and_Carcass_Association,Health_QTL,Production_QTL,Reproduction_Association,Reproduction_QTL
9	ASGA0092311	61133228	181	61	62	KNP	2.112698	0.982687	B3GAT1	Exterior_QTL,Meat_and_Carcass_eQTL,Meat_and_Carcass_QTL,Production_Association,Exterior_Association,Health_Association,Meat_and_Carcass_Association,Health_QTL,Production_QTL,Reproduction_Association,Reproduction_QTL
9	ASGA0043556	62166031	181	62	63	KNP	2.114681	0.982771	GUCY1A2	Exterior_QTL,Meat_and_Carcass_eQTL,Meat_and_Carcass_QTL,Production_Association,Exterior_Association,Health_Association,Meat_and_Carcass_Association,Health_QTL,Production_QTL,Reproduction_Association,Reproduction_QTL

9	MARC0004454	63725198	181	63	64	KNP	2.115131	0.982791	FMO1,FMO2,FMO3,FMO4,LOC100523745,MROH9	Exterior_QTL,Meat_and_Carcass_eQTL,Meat_and_Carcass_QTL,Production_Association,Reproduction_QTL,Health_Association,Meat_and_Carcass_Association,Health_QTL,Production_QTL,Reproduction_Association,Exterior_Association
9	ASGA0043606	64837494	181	64	65	KNP	2.115013	0.982786	ATP2B4,BTG2,ETNK2,FMOD,GOLT1A,KISS1,LAX1,OPTC,PLEKHA6,PRELP,REN,SNRPE,SOX13,ZC3H11A	Exterior_QTL,Meat_and_Carcass_eQTL,Meat_and_Carcass_QTL,Production_Association,Exterior_Association,Health_Association,Meat_and_Carcass_Association,Health_QTL,Production_QTL,Reproduction_Association,Reproduction_QTL
9	ALGA0115869	65280663	181	65	66	KNP	2.115272	0.982797	CNTN2,DSTYK,LRRN2,MDM4,NFASC,NUAK2,PIK3C2B,PLEKHA6,PPP1R15B,RBBP5,TMCC2,TMEM81	Exterior_QTL,Meat_and_Carcass_eQTL,Meat_and_Carcass_QTL,Production_Association,Exterior_Association,Health_Association,Meat_and_Carcass_Association,Health_QTL,Production_QTL,Reproduction_Association,Reproduction_QTL

9	ASGA0093453	66461896	181	66	67	KNP	2.115138	0.982791	AVPR1B,C9H1orf186,CDK18,ELK4,FAM72A,KLHDC8A,LEMD1,LOC100627879,MFSD4A,NUAK2,NUCKS1,PM20D1,RAB29,RAB7B,SLC26A9,SLC41A1,SLC45A3,SRGAP2	Exterior_QTL,Meat_and_Carcass_eQTL,Meat_and_Carcass_QTL,Production_Association,Exterior_Association,Health_Association,Meat_and_Carcass_Association,Health_QTL,Production_QTL,Reproduction_Association,Reproduction_QTL
9	ASGA0045549	67249790	181	67	68	KNP	2.115148	0.982791	C4BPA,C4BPB,C9H1orf116,CD55,DYRK3,EIF2D,FCAMR,FCMR,IKBKE,IL10,IL19,IL20,IL24,MAPKAPK2,PFKFB2,PIGR,RASSF5,SRGAP2,YOD1	Exterior_QTL,Meat_and_Carcass_eQTL,Meat_and_Carcass_QTL,Production_Association,Exterior_Association,Health_Association,Meat_and_Carcass_Association,Health_QTL,Production_QTL,Reproduction_Association,Reproduction_QTL
10	SIRI0001003	61970753	168	61	62	KNP	1.821845	0.965761	LOC110255605	Exterior_QTL,Meat_and_Carcass_eQTL,Meat_and_Carcass_QTL,Production_Association,Exterior_Association,Health_Association,Meat_and_Carcass_Association,Health_QTL,Production_QTL,Reproduction_Association,Reproduction_QTL

10	ALGA0108722	62999619	188	62	63	KNP	2.261964	0.98815	-	Exterior_QTL,Meat_and_Carcass_eQTL,Meat_and_Carcass_QTL,Production_Association,Exterior_Association,Health_Association,Meat_and_Carcass_Association,Health_QTL,Production_QTL,Reproduction_Association,Reproduction_QTL
10	ASGA0048838	63315507	194	63	64	KNP	2.402501	0.991858	ATP5C1,GATA3,ITIH2,ITIH5,KIN,SFMBT2,TAF3	Exterior_QTL,Meat_and_Carcass_eQTL,Meat_and_Carcass_QTL,Production_Association,Reproduction_QTL,Health_Association,Meat_and_Carcass_Association,Health_QTL,Production_QTL,Reproduction_Association,Exterior_Association
10	MARC0036786	64650742	190	64	65	KNP	2.314353	0.989676	IL2RA,PFKFB3,PRKCQ,RBM17,SFMBT2	Exterior_QTL,Meat_and_Carcass_eQTL,Meat_and_Carcass_QTL,Production_Association,Exterior_Association,Health_Association,Meat_and_Carcass_Association,Health_QTL,Production_QTL,Reproduction_Association,Reproduction_QTL

10	ASGA0103853	65026803	190	65	66	KNP	2.313555	0.989654	AKR1C1,AKR1CL1,AKR1E2,ANKRD16,ASB13,CALML5,FAM208B,FBXO18,GDI2,IL15RA,IL2RA,LOC100738684,LOC106505208,LOC106505210,LOC106505211,LOC733635,NET1,TUBAL3,UCN3	Exterior_QTL,Meat_and_Carcass_eQTL,Meat_and_Carcass_QTL,Production_Association,Exterior_Association,Health_Association,Meat_and_Carcass_Association,Health_QTL,Production_QTL,Reproduction_Association,Reproduction_QTL
10	MARC0072301	66613339	179	66	67	KNP	2.071097	0.980825	KLF6,PITRM1	Exterior_QTL,Meat_and_Carcass_eQTL,Meat_and_Carcass_QTL,Production_Association,Exterior_Association,Health_Association,Meat_and_Carcass_Association,Health_QTL,Production_QTL,Reproduction_Association,Reproduction_QTL
10	ASGA0094130	67635924	179	67	68	KNP	2.070036	0.980776	PFKP,PITRM1	Exterior_QTL,Meat_and_Carcass_eQTL,Meat_and_Carcass_QTL,Production_Association,Exterior_Association,Health_Association,Meat_and_Carcass_Association,Health_QTL,Production_QTL,Reproduction_Association,Reproduction_QTL

10	MARC0059864	68856067	179	68	69	KNP	2.070642	0.980804	ADARB2,DIP2C,GTPBP4,LARP4B,LOC100625049,LOC100625534,LOC106505222,WDR37	Exterior_QTL,Meat_and_Carcass_eQTL,Meat_and_Carcass_QTL,Production_Association,Exterior_Association,Health_Association,Meat_and_Carcass_Association,Health_QTL,Production_QTL,Reproduction_Association,Reproduction_QTL
10	ALGA0104885	69100055	179	69	70	KNP	2.069037	0.980729	DIP2C,ZMYND11	Exterior_QTL,Meat_and_Carcass_eQTL,Meat_and_Carcass_QTL,Production_Association,Exterior_Association,Health_Association,Meat_and_Carcass_Association,Health_QTL,Production_QTL,Reproduction_Association,Reproduction_QTL
11	MARC0080552	9660071	173	9	10	KNP	1.938401	0.973713	KL,LOC106507939,PDS5B,STARD13	Meat_and_Carcass_eQTL,Meat_and_Carcass_QTL,Production_Association,Reproduction_Association,Exterior_Association,Health_Association,Reproduction_QTL,Meat_and_Carcass_Association,Health_QTL,Production_QTL,Exterior_QTL
11	ALGA0060733	10045631	173	10	11	KNP	1.938362	0.97371	LOC106507939,RFC3	Meat_and_Carcass_eQTL,Meat_and_Carcass_QTL,Production_Association,Reproduction_QTL,Exterior_Association,Health_Association,Meat_and_Carcass_Association,Health_QTL,Production_QTL,Reproduction_Association,Exterior_QTL

11	ALGA0060867	11961785	173	11	12	KNP	1.938171	0.973699	DCLK1,MAB21L1,NBEA	Meat_and_Carcass_eQTL,Meat_and_Carcass_QTL,Production_Association,Reproduction_QTL,Exterior_Association,Health_Association,Meat_and_Carcass_Association,Health_QTL,Production_QTL,Reproduction_Association,Exterior_QTL
11	H3GA0031340	12009041	173	12	13	KNP	1.9365	0.973597	ALG5,CCNA1,DCLK1,EXOSC8,LOC100624094,LOC106505265,RFXAP,SERTM1,SMAD9,SPG20,SUPT20H	Meat_and_Carcass_eQTL,Meat_and_Carcass_QTL,Production_Association,Reproduction_QTL,Exterior_Association,Health_Association,Meat_and_Carcass_Association,Health_QTL,Production_QTL,Reproduction_Association,Exterior_QTL
11	MARC0070993	13974981	173	13	14	KNP	1.935254	0.97352	FREM2,POSTN,TRPC4,UFM1	Meat_and_Carcass_eQTL,Meat_and_Carcass_QTL,Production_Association,Reproduction_QTL,Exterior_Association,Health_Association,Meat_and_Carcass_Association,Health_QTL,Production_QTL,Reproduction_Association,Exterior_QTL
11	ALGA0060969	14182936	173	14	15	KNP	1.937815	0.973677	COG6,FREM2,LHFP,PROSER1,STOML3	Meat_and_Carcass_eQTL,Meat_and_Carcass_QTL,Production_Association,Reproduction_QTL,Exterior_Association,Health_Association,Meat_and_Carcass_Association,Health_QTL,Production_QTL,Reproduction_Association,Exterior_QTL

11	ALGA0061057	15848668	172	15	16	KNP	1.915615	0.972293	ALG11,ATP7B,CCDC70,CKAP2,FOXO1,LOC100625564,LOC102163801,LOC106505276,MRPS31,NEK3,NEK5,SLC25A15,THSD1,VPS36	Exterior_QTL,Meat_and_Carcass_eQTL,Meat_and_Carcass_QTL,Production_Association,Exterior_Association,Health_Association,Meat_and_Carcass_Association,Health_QTL,Production_QTL,Reproduction_Association,Reproduction_QTL
11	ASGA0099950	16755311	177	16	17	KNP	2.026066	0.978621	DLEU7,FAM124A,INTS6,LOC100625149,LOC102163801,LOC106505279,RNASEH2B,SERPINE3,WDFY2	Meat_and_Carcass_eQTL,Meat_and_Carcass_QTL,Production_Association,Reproduction_QTL,Exterior_Association,Health_Association,Meat_and_Carcass_Association,Health_QTL,Production_QTL,Reproduction_Association,Exterior_QTL
11	MARC0111777	17633008	177	17	18	KNP	2.026705	0.978654	KCNRG,KPNA3,SPRYD7,TRIM13	Exterior_QTL,Meat_and_Carcass_QTL,Meat_and_Carcass_eQTL,Production_Association,Exterior_Association,Health_Association,Meat_and_Carcass_Association,Health_QTL,Production_QTL,Reproduction_Association,Reproduction_QTL

11	ASGA0051811	70965416	161	70	71	KNP	1.672885	0.952825	FGF14,ITGBL1,NALCN,TPP2	Exterior_QTL,Meat_and_Carcass_eQTL,Meat_and_Carcass_QTL,Production_Association,Exterior_Association,Health_Association,Meat_and_Carcass_Association,Health_QTL,Production_QTL,Reproduction_Association,Reproduction_QTL
11	ALGA0063685	71085698	161	71	72	KNP	1.672363	0.952774	BIVM,CCDC168,ERCC5,KDELC1,LOC102165734,METTL21C,SLC10A2,TEX30,TPP2	Exterior_QTL,Meat_and_Carcass_eQTL,Meat_and_Carcass_QTL,Production_Association,Exterior_Association,Health_Association,Meat_and_Carcass_Association,Health_QTL,Production_QTL,Reproduction_Association,Reproduction_QTL
12	ASGA0054449	38680957	160	38	39	KNP	1.650139	0.950543	AATF,ACACA,C12H17orf78,DHRS11,DUSP14,GGNBP2,LHX1,LOC110255902,MRM1,MYO19,PIGW,SYNRG,TADA2A,ZNHIT3	Meat_and_Carcass_eQTL,Meat_and_Carcass_QTL,Production_Association,Exterior_Association,Health_Association,Reproduction_QTL,Meat_and_Carcass_Association,Health_QTL,Production_QTL,Reproduction_Association,Exterior_QTL

12	ASGA0054460	39721723	160	39	40	KNP	1.648694	0.950395	AP2B1,C12H17orf50,CCL14,CCL16,CCL3L1,CCL4,CCL5,DDX52,GAS2L2,HEATR6,HEATR9,HNF1B,LOC100515857,LOC100516039,LOC110256043,LOC110256045,LOC110256053,MMP28,PEX12,RASL10B,SLFN11,SYNRG,TAF15,UNC45B	Exterior_QTL,Meat_and_Carcass_eQTL,Meat_and_Carcass_QTL,Production_Association,Exterior_Association,Health_Association,Reproduction_QTL,Meat_and_Carcass_Association,Health_QTL,Production_QTL,Reproduction_Association
12	ASGA0085898	40155934	160	40	41	KNP	1.648958	0.950422	CCL1,CCL11,CCL2,CCL8,CCT6B,FNDC8,LIG3,LOC100627824,LOC110256055,NLE1,RAD51D,RFFL,TMEM132E,UNC45B	Meat_and_Carcass_eQTL,Meat_and_Carcass_QTL,Production_Association,Exterior_QTL,Health_Association,Reproduction_QTL,Meat_and_Carcass_Association,Health_QTL,Production_QTL,Reproduction_Association,Exterior_Association

12	DRGA0011793	48942747	162	48	49	KNP	1.693238	0.954795	CLUH,DPH1,HIC1,LOC102165602,METTL16,MNT,OVCA2,PAFAH1B1,RAP1GAP2,RPA1,RTN4RL1,SGSM2,SMG6,SMYD4,SRR,TSR1	Meat_and_Carcass_eQTL,Meat_and_Carcass_QTL,Production_Association,Exterior_QTL,Health_Association,Reproduction_QTL,Meat_and_Carcass_Association,Health_QTL,Production_QTL,Reproduction_Association,Exterior_Association
13	MARC0065794	8966952	161	8	9	KNP	1.66916	0.952457	ZNF385D	Meat_and_Carcass_eQTL,Meat_and_Carcass_QTL,Production_Association,Reproduction_QTL,Exterior_Association,Health_Association,Meat_and_Carcass_Association,Health_QTL,Production_QTL,Reproduction_Association,Exterior_QTL
13	H3GA0035400	9980277	161	9	10	KNP	1.672979	0.952834	UBE2E2,ZNF385D	Meat_and_Carcass_eQTL,Meat_and_Carcass_QTL,Production_Association,Reproduction_Association,Exterior_Association,Health_Association,Reproduction_QTL,Meat_and_Carcass_Association,Health_QTL,Production_QTL,Exterior_QTL
13	ALGA0067932	10215894	161	10	11	KNP	1.672827	0.952819	LOC102160336,NKIRAS1,NR1D2,RPL15,THRB,UBE2E2	Meat_and_Carcass_eQTL,Meat_and_Carcass_QTL,Production_Association,Reproduction_QTL,Exterior_Association,Health_Association,Meat_and_Carcass_Association,Health_QTL,Production_QTL,Reproduction_Association,Exterior_QTL

13	ASGA0096967	11570366	161	11	12	KNP	1.672564	0.952793	THRB	Meat_and_Carcass_eQTL,Meat_and_Carcass_QTL,Production_Association,Reproduction_QTL,Exterior_Association,Health_Association,Meat_and_Carcass_Association,Health_QTL,Production_QTL,Reproduction_Association,Exterior_QTL
13	DRGA0012091	15494885	171	15	16	KNP	1.89369	0.970867	RBMS3	Exterior_QTL,Meat_and_Carcass_eQTL,Meat_and_Carcass_QTL,Production_Association,Exterior_Association,Health_Association,Meat_and_Carcass_Association,Health_QTL,Production_QTL,Reproduction_Association,Reproduction_QTL
13	ALGA0068504	16135337	167	16	17	KNP	1.805178	0.964477	GADL1,RBMS3,TGFBR2	Meat_and_Carcass_eQTL,Meat_and_Carcass_QTL,Production_Association,Reproduction_QTL,Exterior_Association,Health_Association,Meat_and_Carcass_Association,Health_QTL,Production_QTL,Reproduction_Association,Exterior_QTL
13	MARC0058770	17909752	166	17	18	KNP	1.783586	0.962754	GADL1,LOC100526184,OSBPL10,STT3B	Exterior_QTL,Meat_and_Carcass_QTL,Meat_and_Carcass_eQTL,Production_Association,Exterior_Association,Health_Association,Meat_and_Carcass_Association,Health_QTL,Production_QTL,Reproduction_Association,Reproduction_QTL

13	ASGA0098365	22824005	163	22	23	KNP	1.716083	0.956927	ACAA1,C13H3orf35,CTDSPL,DCLK3,DLEC1,GOLGA4,ITGA9,LOC110256264,MYD88,PLCD1,TRANK1,VILL	Exterior_QTL,Meat_and_Carcass_QTL,Meat_and_Carcass_eQTL,Production_Association,Exterior_Association,Health_Association,Meat_and_Carcass_Association,Health_QTL,Production_QTL,Reproduction_Association,Reproduction_QTL
13	ASGA0056663	23167386	163	23	24	KNP	1.7156	0.956882	ACAA1,ACVR2B,CCR8,CSRNP1,CX3CR1,EXOG,GORASP1,OXSR1,SCN10A,SCN11A,SCN5A,SLC22A13,SLC22A14,TTC21A,WDR48,XIRP1,XYLB	Exterior_QTL,Meat_and_Carcass_QTL,Meat_and_Carcass_eQTL,Production_Association,Exterior_Association,Health_Association,Reproduction_QTL,Meat_and_Carcass_Association,Health_QTL,Production_QTL,Reproduction_Association
13	MARC0045523	27937972	191	27	28	KNP	2.336396	0.990265	ABHD5,ANO10,KIAA1143,KIF15,LOC100626731,LOC100738134,LOC110256583,TCAIM,TOPAZ1,ZKSCAN7,ZNF197,ZNF35,ZNF445,ZNF660	Exterior_QTL,Meat_and_Carcass_QTL,Meat_and_Carcass_eQTL,Production_Association,Exterior_Association,Health_Association,Reproduction_QTL,Meat_and_Carcass_Association,Health_QTL,Production_QTL,Reproduction_Association

13	ASGA0057022	28681237	191	28	29	KNP	2.336553	0.990269	CDCP1,CLEC3B,EXOSC7,KIF15,LARS2,LIMD1,LZTFL1,SACM1L,SLC6A20,TMEM158,TMEM42,ZDHHC3	Exterior_QTL,Meat_and_Carcass_QTL,Meat_and_Carcass_eQTL,Production_Association,Exterior_Association,Health_Association,Reproduction_QTL,Meat_and_Carcass_Association,Health_QTL,Production_QTL,Reproduction_Association
13	M1GA0017486	29703245	191	29	30	KNP	2.335968	0.990254	ALS2CL,CCDC12,CCR1,CCR2,CCR3,CCR5,CCR9,CCRL2,CXCR6,FAM240A,FYCO1,LOC100515578,LRRC2,LTF,LZTFL1,MYL3,NBEAL2,PRSS46,PRSS50,PTH1R,RTP3,SETD2,TDGF1,XCR1	Exterior_QTL,Meat_and_Carcass_QTL,Meat_and_Carcass_eQTL,Production_Association,Exterior_Association,Health_Association,Reproduction_QTL,Meat_and_Carcass_Association,Health_QTL,Production_QTL,Reproduction_Association

13	H3GA0036111	30634018	191	30	31	KNP	2.335637	0.990245	CSPG5,DHX30,ELP6,KIF9,KLHL18,LOC100736951,LOC106505596,LOC110256441,MAP4,NPG1,NPG4,PMAP-23,PMAP-36,PR39,PTPN23,SCAP,SMARCC1	Exterior_QTL,Meat_and_Carcass_eQTL,Meat_and_Carcass_QTL,Production_Association,Exterior_Association,Health_Association,Reproduction_QTL,Meat_and_Carcass_Association,Health_QTL,Production_QTL,Reproduction_Association

13	ALGA0069410	31459654	191	31	32	KNP	2.336162	0.990259	AMT,ARIH2,ARIH2OS,ATRIP,C13H3orf62,C13H3orf84,CCDC36,CCDC51,CCDC71,CELSR3,COL7A1,DALRD3,GPX1,IMPDH2,IP6K2,KLHDC8B,LAMB2,LOC100156879,LOC110255221,NCKIPSD,NDUFAF3,NME6,P4HTM,PFKFB4,PLXNB1,PRKAR2A,QARS,QRICH1,RHOA,SHISA5,SLC25A20,SLC26A6,TCTA,TMEM89,TREX1,UCN2,USP19,USP4,WDR6	Exterior_QTL,Meat_and_Carcass_eQTL,Meat_and_Carcass_QTL,Production_Association,Reproduction_QTL,Health_Association,Meat_and_Carcass_Association,Health_QTL,Production_QTL,Reproduction_Association,Exterior_Association

13	ALGA0069439	32059847	191	32	33	KNP	2.336381	0.990264	AMT,APEH,BSN,CACNA2D2,CAMKV,CDHR4,DAG1,FAM212A,GMPPB,GNAI2,GNAT1,HYAL1,HYAL2,HYAL3,IFRD2,IP6K1,LOC100517372,LSMEM2,MON1A,MST1,MST1R,NAT6,NICN1,NPRL2,RASSF1,RBM5,RBM6,RNF123,SEMA3B,SEMA3F,SLC38A3,TMEM115,TRAIP,TUSC2,UBA7,ZMYND10	Exterior_QTL,Meat_and_Carcass_QTL,Meat_and_Carcass_eQTL,Production_Association,Exterior_Association,Health_Association,Reproduction_QTL,Meat_and_Carcass_Association,Health_QTL,Production_QTL,Reproduction_Association
13	ALGA0069484	33331986	191	33	34	KNP	2.336528	0.990268	C13H3orf18,CISH,DCAF1,DOCK3,GRM2,HEMK1,MANF,MAPKAPK3,RAD54L2,RBM15B,TEX264	Exterior_QTL,Meat_and_Carcass_QTL,Meat_and_Carcass_eQTL,Production_Association,Exterior_Association,Health_Association,Reproduction_QTL,Meat_and_Carcass_Association,Health_QTL,Production_QTL,Reproduction_Association

13	ALGA0069552	34702780	191	34	35	KNP	2.335687	0.990246	ABHD14A, ABHD14B, ACY1,BAP1,DNAH1,DUSP7,GLT8D1,GLYCTK,GNL3,GPR62,IQCF3,ITIH1,ITIH3,ITIH4,LOC100153598,LOC100620512,LOC100620649,LOC100738931,LOC100738971,LOC100739013,NEK4,NISCH,NT5DC2,PARP3,PBRM1,PCBP4,PHF7,POC1A,PPM1M,RPL29,RRP9,SEMA3G,SPCS1,STAB1,TLR9,TNNC1,TWF2,WDR82	Meat_and_Carcass_eQTL,Meat_and_Carcass_QTL,Production_Association,Exterior_QTL,Exterior_Association,Health_QTL,Meat_and_Carcass_Association,Health_Association,Production_QTL,Reproduction_Association,Reproduction_QTL
13	MARC0076574	35855230	191	35	36	KNP	2.336331	0.990263	ACTR8,CACNA1D,CHDH,DCP1A,IL17RB,LOC100153598,PRKCD,RFT1,SELENOK,SFMBT1,TKT	Meat_and_Carcass_eQTL,Meat_and_Carcass_QTL,Production_Association,Exterior_QTL,Exterior_Association,Health_Association,Meat_and_Carcass_Association,Health_QTL,Production_QTL,Reproduction_Association,Reproduction_QTL

13	ALGA0069653	36660315	191	36	37	KNP	2.335746	0.990248	CACNA2D3,LRTM1	Meat_and_Carcass_QTL,Meat_and_Carcass_eQTL,Production_Association,Exterior_QTL,Health_Association,Reproduction_QTL,Meat_and_Carcass_Association,Health_QTL,Production_QTL,Reproduction_Association,Exterior_Association
13	MARC0054503	37351056	191	37	38	KNP	2.33649	0.990267	CACNA2D3,ERC2,WNT5A	Meat_and_Carcass_eQTL,Meat_and_Carcass_QTL,Production_Association,Exterior_QTL,Health_Association,Reproduction_QTL,Meat_and_Carcass_Association,Health_QTL,Production_QTL,Reproduction_Association,Exterior_Association
13	ALGA0110165	38233628	191	38	39	KNP	2.336034	0.990255	ARHGEF3,CCDC66,ERC2,FAM208A,IL17RD	Meat_and_Carcass_eQTL,Meat_and_Carcass_QTL,Production_Association,Exterior_Association,Health_Association,Reproduction_QTL,Meat_and_Carcass_Association,Health_QTL,Production_QTL,Reproduction_Association,Exterior_QTL
13	ALGA0069724	39238572	191	39	40	KNP	2.336163	0.990259	ABHD6,APPL1,ARF4,ASB14,DENND6A,DNAH12,DNASE1L3,FLNB,HESX1,IL17RD,PDE12,SLMAP	Exterior_QTL,Meat_and_Carcass_eQTL,Meat_and_Carcass_QTL,Production_Association,Exterior_Association,Health_Association,Reproduction_QTL,Meat_and_Carcass_Association,Health_QTL,Production_QTL,Reproduction_Association

13	ALGA0102993	40330016	191	40	41	KNP	2.336101	0.990257	ABHD6,ACOX2,C13H3orf67,FAM107A,FAM3D,KCTD6,LOC100515579,PDHB,PXK,RPP14	Meat_and_Carcass_eQTL,Meat_and_Carcass_QTL,Production_Association,Exterior_QTL,Health_Association,Reproduction_QTL,Meat_and_Carcass_Association,Health_QTL,Production_QTL,Reproduction_Association,Exterior_Association
13	H3GA0036364	41704692	191	41	42	KNP	2.336246	0.990261	FHIT	Meat_and_Carcass_eQTL,Meat_and_Carcass_QTL,Production_Association,Exterior_QTL,Health_Association,Reproduction_QTL,Meat_and_Carcass_Association,Health_QTL,Production_QTL,Reproduction_Association,Exterior_Association
13	ALGA0069851	42601463	191	42	43	KNP	2.33635	0.990263	FHIT	Meat_and_Carcass_eQTL,Meat_and_Carcass_QTL,Production_Association,Exterior_Association,Health_Association,Reproduction_QTL,Meat_and_Carcass_Association,Health_QTL,Production_QTL,Reproduction_Association,Exterior_QTL
13	ALGA0069881	43693866	191	43	44	KNP	2.336574	0.990269	C13H3orf14,CADPS,FEZF2,PTPRG	Meat_and_Carcass_eQTL,Meat_and_Carcass_QTL,Production_Association,Exterior_QTL,Health_QTL,Reproduction_QTL,Meat_and_Carcass_Association,Health_Association,Production_QTL,Reproduction_Association,Exterior_Association

13	ALGA0069922	44714762	191	44	45	KNP	2.336329	0.990263	CADPS,SYNPR	Meat_and_Carcass_eQTL,Meat_and_Carcass_QTL,Production_Association,Exterior_QTL,Health_Association,Reproduction_QTL,Meat_and_Carcass_Association,Health_QTL,Production_QTL,Reproduction_Association,Exterior_Association
13	ALGA0114810	45196075	191	45	46	KNP	2.336267	0.990261	ATXN7,LOC106505631,PRICKLE2,PSMD6,SNTN,SYNPR,THOC7	Meat_and_Carcass_eQTL,Meat_and_Carcass_QTL,Production_Association,Exterior_QTL,Health_Association,Reproduction_QTL,Meat_and_Carcass_Association,Health_QTL,Production_QTL,Reproduction_Association,Exterior_Association
16	ALGA0088683	3917163	183	3	4	KNP	2.159549	0.984596	DNAH5,FAM105A,TRIO	Meat_and_Carcass_eQTL,Meat_and_Carcass_QTL,Production_Association,Reproduction_QTL,Exterior_Association,Health_Association,Meat_and_Carcass_Association,Health_QTL,Production_QTL,Reproduction_Association,Exterior_QTL
16	ALGA0122084	4191429	183	4	5	KNP	2.159018	0.984576	ANKH,FAM105A,FBXL7,OTULIN	Meat_and_Carcass_eQTL,Meat_and_Carcass_QTL,Production_Association,Reproduction_QTL,Exterior_Association,Health_Association,Meat_and_Carcass_Association,Health_QTL,Production_QTL,Reproduction_Association,Exterior_QTL

16	ASGA0072056	5754491	183	5	6	KNP	2.159442	0.984592	FBXL7,LOC100521410,MARCH11,MYO10,RETREG1,ZNF622	Meat_and_Carcass_eQTL,Meat_and_Carcass_QTL,Production_Association,Reproduction_QTL,Exterior_Association,Health_QTL,Meat_and_Carcass_Association,Health_Association,Production_QTL,Reproduction_Association,Exterior_QTL
16	ALGA0088840	6013623	183	6	7	KNP	2.157931	0.984533	BASP1,LOC102168105,MYO10	Meat_and_Carcass_eQTL,Meat_and_Carcass_QTL,Production_Association,Reproduction_QTL,Exterior_Association,Health_Association,Meat_and_Carcass_Association,Health_QTL,Production_QTL,Reproduction_Association,Exterior_QTL
16	MARC0054728	18818606	163	18	19	KNP	1.714649	0.956795	LOC110257335,MTMR12,NPR3,PDZD2,SUB1,ZFR	Exterior_QTL,Meat_and_Carcass_QTL,Meat_and_Carcass_eQTL,Production_Association,Exterior_Association,Health_Association,Meat_and_Carcass_Association,Health_QTL,Production_QTL,Reproduction_Association,Reproduction_QTL
16	ASGA0103355	19583421	163	19	20	KNP	1.717206	0.957029	ADAMTS12,RXFP3,SLC45A2,TARS	Exterior_QTL,Meat_and_Carcass_eQTL,Meat_and_Carcass_QTL,Production_Association,Exterior_Association,Health_Association,Reproduction_QTL,Meat_and_Carcass_Association,Health_QTL,Production_QTL,Reproduction_Association

16	ALGA0118313	20103323	163	20	21	KNP	1.716925	0.957004	AGXT2,AMACR,BRIX1,C1QTNF3,DNAJC21,PRLR,RAD1,RAI14,SLC45A2,TTC23L	Exterior_QTL,Meat_and_Carcass_QTL,Meat_and_Carcass_eQTL,Production_Association,Exterior_Association,Health_Association,Reproduction_QTL,Meat_and_Carcass_Association,Health_QTL,Production_QTL,Reproduction_Association
16	ALGA0089857	25403364	161	25	26	KNP	1.672776	0.952814	C7,CARD6,MROH2B,PRKAA1,PTGER4,RPL37,TTC33	Exterior_QTL,Meat_and_Carcass_QTL,Meat_and_Carcass_eQTL,Production_Association,Exterior_Association,Health_Association,Meat_and_Carcass_Association,Health_QTL,Production_QTL,Reproduction_Association,Reproduction_QTL
17	ALGA0107858	10373384	162	10	11	KNP	1.694753	0.954939	ANK1,GINS4,GOLGA7,GPAT4,NKX6-3,SFRP1,ZMAT4	Meat_and_Carcass_eQTL,Meat_and_Carcass_QTL,Production_Association,Reproduction_QTL,Exterior_Association,Health_Association,Meat_and_Carcass_Association,Health_QTL,Production_QTL,Reproduction_Association,Exterior_QTL
17	ALGA0123867	13717308	160	13	14	KNP	1.648084	0.950332	LOC100155289,LOC100511540,LOC100511905,PRND,PRNP,PSD3,RASSF2,SLC23A2	Meat_and_Carcass_eQTL,Meat_and_Carcass_QTL,Production_Association,Reproduction_QTL,Exterior_Association,Health_Association,Meat_and_Carcass_Association,Health_QTL,Production_QTL,Reproduction_Association,Exterior_QTL

17	CASI0011282	14802732	160	14	15	KNP	1.650315	0.950561	C17H20orf196,CDS2,CHGB,CRLS1,GPCPD1,LOC100513319,LRRN4,MCM8,PCNA,PROKR2,SLC23A2,TMEM230,TRMT6	Meat_and_Carcass_eQTL,Meat_and_Carcass_QTL,Production_Association,Reproduction_QTL,Exterior_Association,Health_Association,Meat_and_Carcass_Association,Health_QTL,Production_QTL,Reproduction_Association,Exterior_QTL
17	DRGA0016582	15949323	197	15	16	KNP	2.469125	0.993228	BMP2,FERMT1	Exterior_QTL,Meat_and_Carcass_eQTL,Meat_and_Carcass_QTL,Production_Association,Exterior_Association,Health_Association,Meat_and_Carcass_Association,Health_QTL,Production_QTL,Reproduction_Association,Reproduction_QTL
17	MARC0028591	16634316	197	16	17	KNP	2.46865	0.993219	HAO1,TMX4	Meat_and_Carcass_eQTL,Meat_and_Carcass_QTL,Production_Association,Reproduction_QTL,Exterior_Association,Health_Association,Meat_and_Carcass_Association,Health_QTL,Production_QTL,Reproduction_Association,Exterior_QTL
17	ALGA0093494	17194880	197	17	18	KNP	2.469065	0.993227	PLCB1,PLCB4	Exterior_QTL,Meat_and_Carcass_QTL,Meat_and_Carcass_eQTL,Production_Association,Exterior_Association,Health_Association,Meat_and_Carcass_Association,Health_QTL,Production_QTL,Reproduction_Association,Reproduction_QTL

17	MARC0016394	18910462	198	18	19	KNP	2.491212	0.993635	ANKEF1,LAMP5,PAK5,PLCB4	Exterior_QTL,Meat_and_Carcass_QTL,Meat_and_Carcass_eQTL,Production_Association,Exterior_Association,Health_Association,Meat_and_Carcass_Association,Health_QTL,Production_QTL,Reproduction_Association,Reproduction_QTL
17	ALGA0118100	19134134	198	19	20	KNP	2.49144	0.993639	JAG1,MKKS,SLX4IP,SNAP25	Exterior_QTL,Meat_and_Carcass_eQTL,Meat_and_Carcass_QTL,Production_Association,Exterior_Association,Health_Association,Reproduction_QTL,Meat_and_Carcass_Association,Health_QTL,Production_QTL,Reproduction_Association
17	ALGA0093681	20084543	198	20	21	KNP	2.490282	0.993618	BTBD3	Exterior_QTL,Meat_and_Carcass_QTL,Meat_and_Carcass_eQTL,Production_Association,Exterior_Association,Health_Association,Reproduction_QTL,Meat_and_Carcass_Association,Health_QTL,Production_QTL,Reproduction_Association
17	ALGA0093770	21990952	177	21	22	KNP	2.024354	0.978533	ISM1,SPTLC3,TASP1	Exterior_QTL,Meat_and_Carcass_QTL,Meat_and_Carcass_eQTL,Production_Association,Exterior_Association,Health_Association,Meat_and_Carcass_Association,Health_QTL,Production_QTL,Reproduction_Association,Reproduction_QTL

17	ALGA0093784	22236616	177	22	23	KNP	2.026117	0.978624	ESF1,FLRT3,ISM1,MACROD2,NDUFAF5,SEL1L2,TASP1	Exterior_QTL,Meat_and_Carcass_QTL,Meat_and_Carcass_eQTL,Production_Association,Exterior_Association,Health_Association,Meat_and_Carcass_Association,Health_QTL,Production_QTL,Reproduction_Association,Reproduction_QTL
17	MARC0115548	25624531	170	25	26	KNP	1.871756	0.96938	BFSP1,DSTN,KIF16B,OTOR,PCSK2,SNRPB2	Exterior_QTL,Meat_and_Carcass_QTL,Meat_and_Carcass_eQTL,Production_Association,Exterior_Association,Health_Association,Meat_and_Carcass_Association,Health_QTL,Production_QTL,Reproduction_Association,Reproduction_QTL
17	ASGA0075970	26901869	168	26	27	KNP	1.827764	0.966208	BANF2,DSTN,DTD1,DZANK1,KAT14,LOC102162195,MGME1,OVOL2,PET117,POLR3F,RBBP9,RRBP1,SCP2D1,SEC23B,SNX5,ZNF133	Exterior_QTL,Meat_and_Carcass_eQTL,Meat_and_Carcass_QTL,Production_Association,Exterior_Association,Health_Association,Reproduction_QTL,Meat_and_Carcass_Association,Health_QTL,Production_QTL,Reproduction_Association
17	ASGA0075984	27022887	168	27	28	KNP	1.827245	0.966169	RIN2,SLC24A3	Exterior_QTL,Meat_and_Carcass_QTL,Meat_and_Carcass_eQTL,Production_Association,Exterior_Association,Health_Association,Reproduction_QTL,Meat_and_Carcass_Association,Health_QTL,Production_QTL,Reproduction_Association

1	INRA0006085	220998409	409	220	221	WRH	3.698055	0.999891	DMRT1,DMRT2,DMRT3	Meat_and_Carcass_QTL,Meat_and_Carcass_eQTL,Exterior_QTL,Exterior_Association,Health_Association,Health_QTL,Production_QTL,Reproduction_QTL
1	ALGA0008078	221963949	471	221	222	WRH	4.510272	0.999997	C1H9orf66,DOCK8,KANK1,LOC100155087,LOC100156293,PGM5,TMEM252	Meat_and_Carcass_QTL,Meat_and_Carcass_eQTL,Exterior_QTL,Health_Association,Meat_and_Carcass_Association,Health_QTL,Production_QTL,Reproduction_QTL
1	ASGA0005841	222033049	471	222	223	WRH	4.510362	0.999997	APBA1,FAM122A,FAM189A2,FXN,PIP5K1B,TJP2	Meat_and_Carcass_QTL,Meat_and_Carcass_eQTL,Exterior_QTL,Health_Association,Meat_and_Carcass_Association,Health_QTL,Production_QTL,Reproduction_Association,Reproduction_QTL
1	INRA0006184	223132671	430	223	224	WRH	3.97328	0.999965	C1H9orf135,KLF9,MAMDC2,PTAR1,SMC5,TRPM3	Meat_and_Carcass_QTL,Meat_and_Carcass_eQTL,Exterior_QTL,Health_Association,Health_QTL,Production_QTL,Reproduction_QTL
1	INRA0006470	232800955	439	232	233	WRH	4.091079	0.999979	TLE4	Meat_and_Carcass_QTL,Meat_and_Carcass_eQTL,Exterior_QTL,Health_Association,Meat_and_Carcass_Association,Health_QTL,Production_QTL,Reproduction_QTL

1	MARC0047151	233901042	445	233	234	WRH	4.168897	0.999985	NPAP1	Meat_and_Carcass_QTL,Meat_and_Carcass_eQTL,Exterior_QTL,Health_QTL,Meat_and_Carcass_Association,Health_Association,Production_QTL,Reproduction_QTL
1	MARC0003821	234538395	447	234	235	WRH	4.19617	0.999986	LOC100524475,LOC102164346,SPATA31D1	Meat_and_Carcass_QTL,Meat_and_Carcass_eQTL,Exterior_QTL,Health_QTL,Meat_and_Carcass_Association,Health_Association,Production_QTL,Reproduction_QTL
1	ASGA0006103	235399725	452	235	236	WRH	4.260108	0.99999	C1H9orf131,DNAJB5,FAM205C,FAM214B,FANCG,LOC100525024,LOC110255297,PHF24,PIGO,STOML2,UNC13B,VCP	Meat_and_Carcass_QTL,Meat_and_Carcass_eQTL,Reproduction_QTL,Meat_and_Carcass_Association,Health_QTL,Production_QTL,Exterior_QTL
3	ASGA0014437	47274629	364	47	48	WRH	3.108824	0.999061	ASTL,CCDC138,DUSP2,EDAR,GCC2,LIMS1,LOC100511376,LOC100623441,LOC100623541,LOC100623656,LOC100624389,SH3RF3,SLC5A7,STARD7,TMEM127	Meat_and_Carcass_eQTL,Meat_and_Carcass_QTL,Production_Association,Exterior_QTL,Health_Association,Reproduction_QTL,Meat_and_Carcass_Association,Health_QTL,Production_QTL,Reproduction_Association,Exterior_Association

3	ALGA0115229	48969590	368	48	49	WRH	3.1601	0.999211	C3H2orf40,LOC102166005,SLC5A7,ST6GAL2,UXS1	Meat_and_Carcass_eQTL,Meat_and_Carcass_QTL,Production_Association,Exterior_QTL,Health_Association,Reproduction_QTL,Meat_and_Carcass_Association,Health_QTL,Production_QTL,Reproduction_Association,Exterior_Association
3	H3GA0055645	49490169	368	49	50	WRH	3.160316	0.999212	C3H2orf49,FHL2,GPR45,LOC110260119,MRPS9,NCK2,POU3F3,TGFBRAP1	Meat_and_Carcass_eQTL,Meat_and_Carcass_QTL,Production_Association,Exterior_QTL,Health_Association,Reproduction_QTL,Meat_and_Carcass_Association,Health_QTL,Production_QTL,Reproduction_Association,Exterior_Association
3	H3GA0009538	52967495	380	52	53	WRH	3.313344	0.999539	CREG2,IL1R1,IL1R2,IL1RL2,MAP4K4,RFX8	Meat_and_Carcass_eQTL,Meat_and_Carcass_QTL,Production_Association,Exterior_QTL,Health_Association,Reproduction_QTL,Meat_and_Carcass_Association,Health_QTL,Production_QTL,Reproduction_Association,Exterior_Association
3	M1GA0004379	53897527	383	53	54	WRH	3.357388	0.999607	CHST10,CNOT11,LOC102163417,LONRF2,NMS,NPAS2,PDCL3,RNF149,RPL31,TBC1D8	Meat_and_Carcass_eQTL,Meat_and_Carcass_QTL,Production_Association,Exterior_Association,Health_Association,Reproduction_QTL,Meat_and_Carcass_Association,Health_QTL,Production_QTL,Reproduction_Association,Exterior_QTL

3	INRA0010628	54225407	382	54	55	WRH	3.344498	0.999588	AFF3,EIF5B,LOC100522987,REV1,TXNDC9	Meat_and_Carcass_eQTL,Meat_and_Carcass_QTL,Production_Association,Exterior_Association,Health_Association,Reproduction_QTL,Meat_and_Carcass_Association,Health_QTL,Production_QTL,Reproduction_Association,Exterior_QTL
7	H3GA0021762	50716500	395	50	51	WRH	3.514787	0.99978	EFL1,IL16,LOC102160759,MEX3B,SAXO2,STARD5,TMC3	Meat_and_Carcass_eQTL,Meat_and_Carcass_QTL,Production_Association,Exterior_QTL,Health_Association,Reproduction_QTL,Meat_and_Carcass_Association,Health_QTL,Production_QTL,Reproduction_Association,Exterior_Association
7	ASGA0034088	51328974	396	51	52	WRH	3.528114	0.999791	ADAMTSL3,BNC1,BTBD1,C7H15orf40,FAM103A1,HDGFL3,SH3GL3,TM6SF1	Meat_and_Carcass_eQTL,Meat_and_Carcass_QTL,Production_Association,Exterior_QTL,Health_Association,Reproduction_QTL,Meat_and_Carcass_Association,Health_QTL,Production_QTL,Reproduction_Association,Exterior_Association
7	M1GA0010423	52192625	396	52	53	WRH	3.527407	0.99979	ALPK3,AP3B2,CPEB1,FSD2,HOMER2,NMB,PDE8A,RPS17,SEC11A,SLC28A1,WDR73,WHAMM,ZNF592,ZNF774,ZSCAN2	Meat_and_Carcass_eQTL,Meat_and_Carcass_QTL,Production_Association,Exterior_QTL,Health_Association,Reproduction_QTL,Meat_and_Carcass_Association,Health_QTL,Production_QTL,Reproduction_Association,Exterior_Association

7	H3GA0021846	53405107	393	53	54	WRH	3.488647	0.999757	ALDH1L1,BLM,CFAP100,CRTC3,FES,FURIN,HDDC3,IQGAP1,KLF15,MAN2A2,RCCD1,SLC41A3,UNC45A,UROC1,ZXDC	Meat_and_Carcass_eQTL,Meat_and_Carcass_QTL,Production_Association,Exterior_Association,Health_Association,Reproduction_QTL,Meat_and_Carcass_Association,Health_QTL,Production_QTL,Reproduction_Association,Exterior_QTL
9	H3GA0027331	47945255	392	47	48	WRH	3.471995	0.999742	ARHGEF12,GRIK4,OAF,POU2F3,TMEM136,TRIM29	Meat_and_Carcass_eQTL,Meat_and_Carcass_QTL,Production_Association,Exterior_QTL,Health_Association,Reproduction_QTL,Meat_and_Carcass_Association,Health_QTL,Production_QTL,Reproduction_Association,Exterior_Association
9	ASGA0043059	48630211	403	48	49	WRH	3.619297	0.999852	GRIK4,LOC100523684,SC5D,SORL1	Meat_and_Carcass_eQTL,Meat_and_Carcass_QTL,Production_Association,Exterior_QTL,Health_Association,Reproduction_QTL,Meat_and_Carcass_Association,Health_QTL,Production_QTL,Reproduction_Association,Exterior_Association
9	ALGA0053020	49378868	403	49	50	WRH	3.619287	0.999852	BSX,C9H11orf63,CLMP,CRTAM,HSPA8,LOC102164776,UBASH3B	Meat_and_Carcass_eQTL,Meat_and_Carcass_QTL,Production_Association,Exterior_QTL,Health_Association,Reproduction_QTL,Meat_and_Carcass_Association,Health_QTL,Production_QTL,Reproduction_Association,Exterior_Association

9	ASGA0095941	50067628	397	50	51	WRH	3.540842	0.999801	CLMP,GRAMD1B,LOC100512247,LOC100513369,LOC100513559,LOC100515746,LOC100518267,LOC100518623,LOC100518799,LOC100518982,LOC100524388,LOC100524576,LOC100524757,LOC100524947,LOC100525119,LOC100525298,LOC100525476,LOC106504899,LOC106504900,LOC106504901,LOC106504905,LOC106504908,LOC106504911,LOC106504912,LOC110262281,LOC110262293,SCN3B,TMEM225,ZNF202	Meat_and_Carcass_eQTL,Meat_and_Carcass_QTL,Production_Association,Exterior_QTL,Health_Association,Reproduction_QTL,Meat_and_Carcass_Association,Health_QTL,Production_QTL,Reproduction_Association,Exterior_Association

9	H3GA0055699	57097854	373	57	58	WRH	3.226561	0.999374	ADAMTS15,ADAMTS8,LOC102165987,NTM,SNX19,ZBTB44	Meat_and_Carcass_eQTL,Meat_and_Carcass_QTL,Production_Association,Exterior_Association,Health_Association,Reproduction_QTL,Meat_and_Carcass_Association,Health_QTL,Production_QTL,Reproduction_Association,Exterior_QTL
14	ALGA0078924	74502555	432	74	75	WRH	3.999779	0.999968	ASCC1,C14H10orf105,CDH23,CHST3,LOC106506033,LOC110256823,PSAP,SLC29A3,SPOCK2,UNC5B,VSIR	Exterior_QTL,Meat_and_Carcass_eQTL,Meat_and_Carcass_QTL,Production_Association,Exterior_Association,Health_Association,Meat_and_Carcass_Association,Health_QTL,Production_QTL,Reproduction_Association,Reproduction_QTL
14	ALGA0079011	75891232	433	75	76	WRH	4.012687	0.99997	ANAPC16,ASCC1,DDIT4,DNAJB12,ECD,FAM149B1,MCU,MICU1,NUDT13,OIT3,P4HA1,PLA2G12B	Exterior_QTL,Meat_and_Carcass_eQTL,Meat_and_Carcass_QTL,Production_Association,Exterior_Association,Health_Association,Meat_and_Carcass_Association,Health_QTL,Production_QTL,Reproduction_Association,Reproduction_QTL

14	CASI0011217	76745141	414	76	77	WRH	3.763082	0.999916	ADK,ANXA7,AP3M1,CAMK2G,CFAP70,CHCHD1,DNAJC9,FAM149B1,FUT11,LOC102160869,LOC106506035,MRPS16,MSS51,MYOZ1,NDST2,PLAU,PPP3CB,SEC24C,SYNPO2L,USP54,VCL,ZSWIM8	Exterior_QTL,Meat_and_Carcass_eQTL,Meat_and_Carcass_QTL,Production_Association,Exterior_Association,Health_Association,Meat_and_Carcass_Association,Health_QTL,Production_QTL,Reproduction_Association,Reproduction_QTL
14	H3GA0041058	77301532	415	77	78	WRH	3.776119	0.99992	ADK,COMTD1,DUPD1,DUSP13,KAT6B,LOC106507128,SAMD8,VDAC2,ZNF503	Exterior_QTL,Meat_and_Carcass_eQTL,Meat_and_Carcass_QTL,Production_Association,Exterior_Association,Health_Association,Meat_and_Carcass_Association,Health_QTL,Production_QTL,Reproduction_Association,Reproduction_QTL
14	H3GA0041083	78045217	398	78	79	WRH	3.553254	0.99981	-	Exterior_QTL,Meat_and_Carcass_eQTL,Meat_and_Carcass_QTL,Production_Association,Exterior_Association,Health_Association,Meat_and_Carcass_Association,Health_QTL,Production_QTL,Reproduction_Association,Reproduction_QTL

14	MARC0011936	91864792	413	91	92	WRH	3.750453	0.999912	C14H10orf10,CXCL12,LOC100157415,RASSF4,TMEM72,ZNF22	Exterior_QTL,Meat_and_Carcass_eQTL,Meat_and_Carcass_QTL,Production_Association,Reproduction_QTL,Health_Association,Meat_and_Carcass_Association,Health_QTL,Production_QTL,Reproduction_Association,Exterior_Association
14	CASI0009035	92806581	414	92	93	WRH	3.762948	0.999916	CISD1,IPMK,LOC100524282,TFAM,UBE2D1,ZNF239,ZNF32	Exterior_QTL,Meat_and_Carcass_eQTL,Meat_and_Carcass_QTL,Production_Association,Exterior_Association,Health_Association,Reproduction_QTL,Meat_and_Carcass_Association,Health_QTL,Production_QTL,Reproduction_Association
14	DRGA0014291	93216469	413	93	94	WRH	3.750537	0.999912	-	Exterior_QTL,Meat_and_Carcass_eQTL,Meat_and_Carcass_QTL,Production_Association,Reproduction_QTL,Health_Association,Meat_and_Carcass_Association,Health_QTL,Production_QTL,Reproduction_Association,Exterior_Association
14	ALGA0080043	94163676	413	94	95	WRH	3.749809	0.999912	LOC106506082,ZWINT	Exterior_QTL,Meat_and_Carcass_eQTL,Meat_and_Carcass_QTL,Production_Association,Reproduction_QTL,Health_Association,Meat_and_Carcass_Association,Health_QTL,Production_QTL,Reproduction_Association,Exterior_Association

14	INRA0045883	95117803	410	95	96	WRH	3.710051	0.999896	PCDH15	Exterior_QTL,Meat_and_Carcass_eQTL,Meat_and_Carcass_QTL,Production_Association,Reproduction_QTL,Health_Association,Meat_and_Carcass_Association,Health_QTL,Production_QTL,Reproduction_Association,Exterior_Association
14	H3GA0041609	96545993	407	96	97	WRH	3.670544	0.999879	LOC102157770,LOC110256792,PCDH15	Exterior_QTL,Meat_and_Carcass_eQTL,Meat_and_Carcass_QTL,Production_Association,Reproduction_QTL,Health_Association,Meat_and_Carcass_Association,Health_QTL,Production_QTL,Reproduction_Association,Exterior_Association
14	ALGA0080212	97858443	413	97	98	WRH	3.750148	0.999912	DKK1,MBL2,PRKG1	Exterior_QTL,Meat_and_Carcass_eQTL,Meat_and_Carcass_QTL,Production_Association,Reproduction_QTL,Health_Association,Meat_and_Carcass_Association,Health_QTL,Production_QTL,Reproduction_Association,Exterior_Association
14	ASGA0065523	98454996	417	98	99	WRH	3.802713	0.999928	A1CF,CSTF2T,PRKG1	Exterior_QTL,Meat_and_Carcass_eQTL,Meat_and_Carcass_QTL,Production_Association,Reproduction_QTL,Health_Association,Meat_and_Carcass_Association,Health_QTL,Production_QTL,Reproduction_Association,Exterior_Association

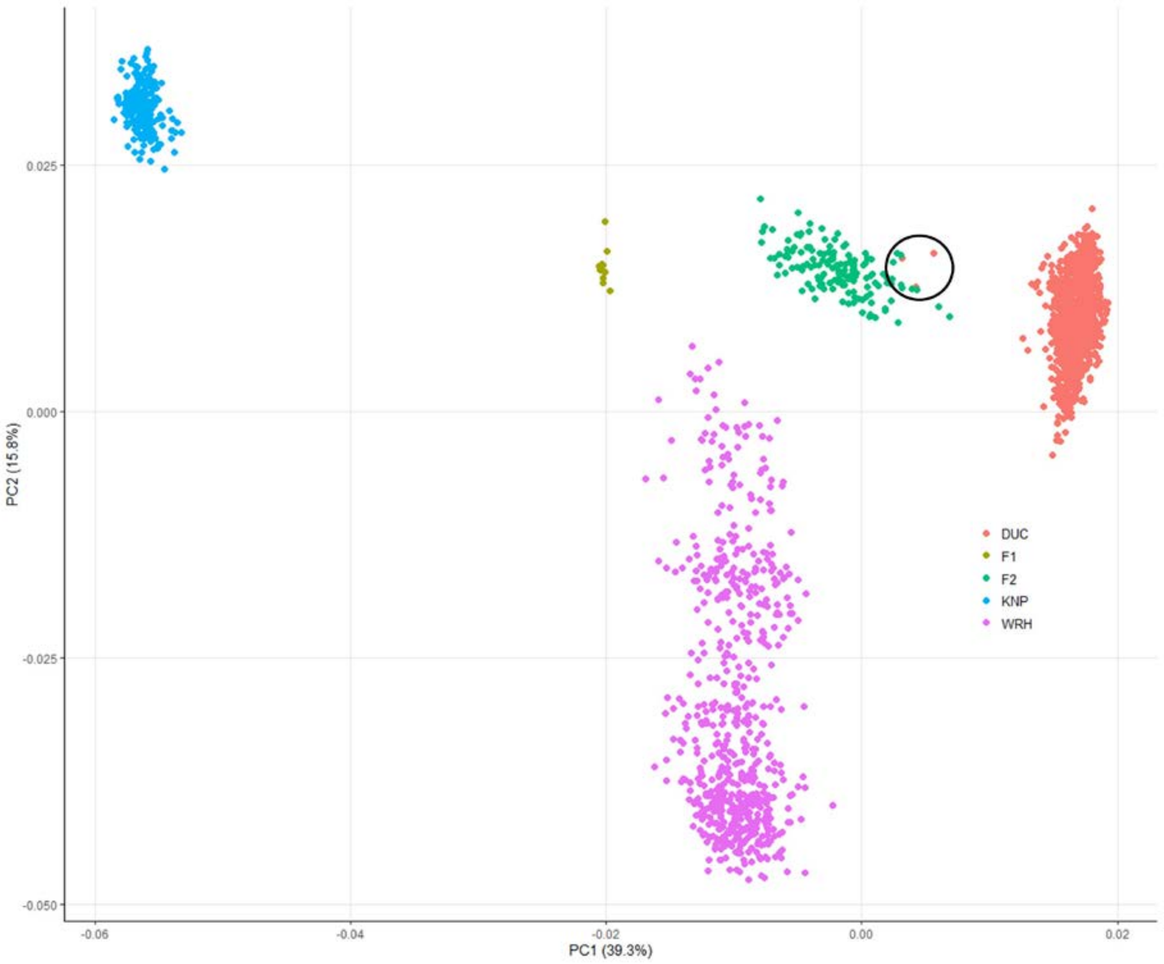

Supplementary Figure 1. PCA plot before excluding three DUC individuals that might be the potentially breed-misclassified. A circle indicates those three DUC individuals. DUC, Korean Duroc; KNP, Korean native pig; F1, DUC × KNP; F2, F1 × DUC; WRH, Woori-Heukdon (F1 × F2).

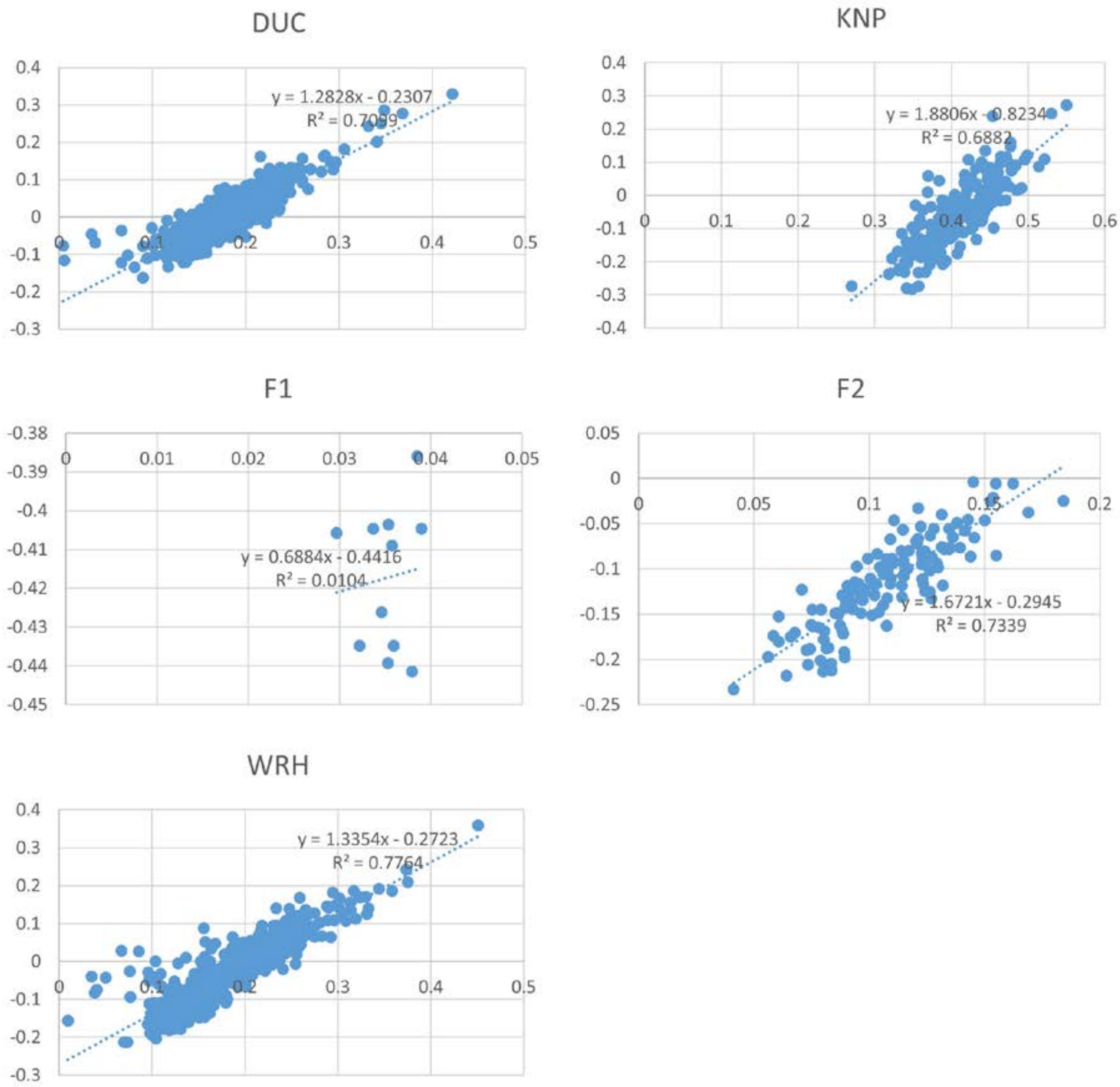

Supplementary Figure 2. Pearson correlation between F_{HOM} and F_{ROH} in five pig populations. DUC, Korean Duroc; KNP, Korean native pig; F1, DUC × KNP; F2, F1 × DUC; WRH, Woori-Heukdon (F1 × F2).
